# Habitat suitability maps for Australian flora and fauna under CMIP6 climate scenarios

**DOI:** 10.1093/gigascience/giae002

**Published:** 2024-03-05

**Authors:** Carla L Archibald, David M Summers, Erin M Graham, Brett A Bryan

**Affiliations:** School of Life and Environmental Sciences, Deakin University, 221 Burwood Hwy, Burwood, Victoria, Australia; UniSA Business, The University of South Australia, GPO Box 2471, Adelaide, Australia; eResearch Centre, James Cook University, James Cook Drive, Townsville, Australia; School of Life and Environmental Sciences, Deakin University, 221 Burwood Hwy, Burwood, Victoria, Australia

**Keywords:** Australia, Atlas of Living Australia, biodiverstiy, bioclimatic variables, climate change, CliMAS, species distribution, species range, MaxEnt, WorldClim

## Abstract

**Background:**

Spatial information about the location and suitability of areas for native plant and animal species under different climate futures is an important input to land use and conservation planning and management. Australia, renowned for its abundant species diversity and endemism, often relies on modeled data to assess species distributions due to the country’s vast size and the challenges associated with conducting on-ground surveys on such a large scale. The objective of this article is to develop habitat suitability maps for Australian flora and fauna under different climate futures.

**Results:**

Using MaxEnt, we produced Australia-wide habitat suitability maps under RCP2.6-SSP1, RCP4.5-SSP2, RCP7.0-SSP3, and RCP8.5-SSP5 climate futures for 1,382 terrestrial vertebrates and 9,251 vascular plants vascular plants at 5 km^2^ for open access. This represents 60% of all Australian mammal species, 77% of amphibian species, 50% of reptile species, 71% of bird species, and 44% of vascular plant species. We also include tabular data, which include summaries of total quality-weighted habitat area of species under different climate scenarios and time periods.

**Conclusions:**

The spatial data supplied can help identify important and sensitive locations for species under various climate futures. Additionally, the supplied tabular data can provide insights into the impacts of climate change on biodiversity in Australia. These habitat suitability maps can be used as input data for landscape and conservation planning or species management, particularly under different climate change scenarios in Australia.

## Graphical Abstract

**Graphical Abstract:**
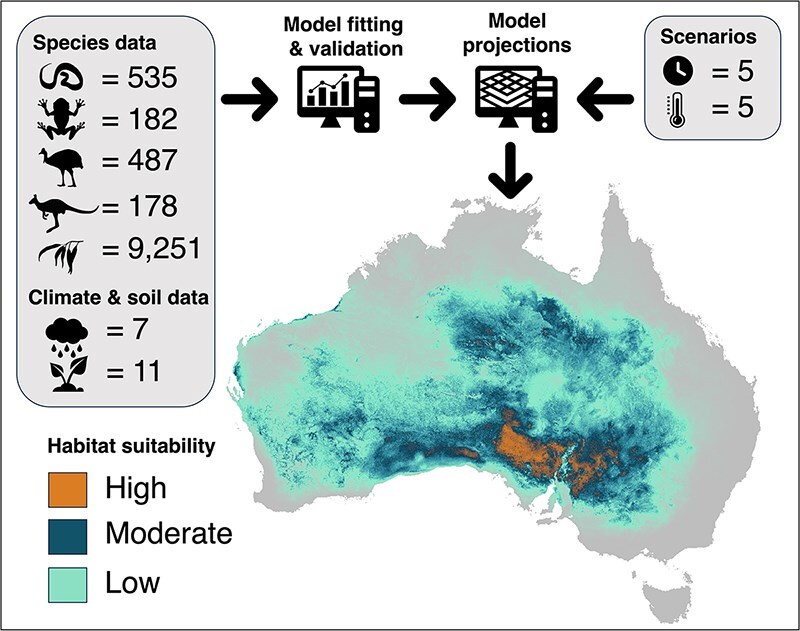

## Introduction

Rich spatial and temporal information about the effect of climatic and environmental change on species distributions is necessary to ensure robust species management and conservation policy more broadly [1–4]. Identifying areas where species occur now, as well as areas that may be suitable in the future, is a crucial aspect of decision-making under uncertainty [4]. The availability of resources for conservation, including financial, staffing, and land availability, is limited and exacerbates the challenge of conservation planning during climate change [3]. These constraints have sparked the need for more strategic landscape and conservation planning methods, such as spatial prioritization, to identify the most effective conservation solutions [5]. Spatial information on where species are now and where suitable areas may be in the future is the foundation of efficient planning for conservation action, particularly in areas where local conditions are more sensitive to climate change [4].

Australia is a hyperdiverse country with high levels of species endemism [6, 7]. Unfortunately, Australia also has some of the highest recorded numbers of contemporary extinctions worldwide, and more than 1,900 species and ecological communities are under threat [8, 9]. Given the extensive and severe range and population declines of many threatened species [9–11], many more species are also predicted to have a high risk of extinction in the future [12]. To ensure the conservation of Australia’s unique biodiversity, identifying and protecting important areas for species such as climate refugia is key to planning for resilience and adaptive capacity [13]. To fulfill this task, underlying data on species location and the habitat suitability of areas for species under different climate futures are required.

There are many ways to assess suitable areas for species, and one popular approach is to use the maximum entropy method (henceforth, MaxEnt). MaxEnt is a niche-based general-purpose machine learning method with a simple and precise mathematical formulation that is particularly well suited for species distribution modeling with presence-only data [14, 15]. Generating MaxEnt models for individual species at continental scales presents challenges around the processing and storage of large volumes of data. A comprehensive spatial dataset known as “CliMAS” [16] modeled 1,872 terrestrial and freshwater vertebrate species distributions using the Intergovernmental Panel on Climate Change’ s (IPCC’ s) Coupled Model Intercomparison Project 3 (CMIP3) future climate projections [17] which were freely available through a web-based portal. Although the CliMAS models led to many applied outcomes [18, 19, 20], the website was retired in 2020, in recognition of the fact that there have been 2 major updates by the IPCC and the current projections are based on CMIP6. For conservation planning to progress, an improved and enlarged suite of freely available spatial data, based on up-to-date climate projections and extended for a much broader range of species including vascular plants, is needed.

We developed habitat suitability maps for Australian flora and fauna under different climate futures using a MaxEnt approach. We produced freely accessible Australia-wide habitat suitability maps for 1,441 terrestrial vertebrates and 9,251 vascular plants. This represents 60% of all Australian mammal species, 77% of amphibian species, 50% of reptile species, 71% of bird species, and 44% of vascular plant species. We fit these models using 7 bioclimatic variables and 11 soil and landscape variables under 4 climate scenarios, 8 general circulation models (GCMs) and 1 ensemble average, and 5 time periods. These habitat suitability maps are best used as input data to represent species or biodiversity values for conservation planning and assessment, particularly under climate change in Australia.

## Methods

**Figure 1:**
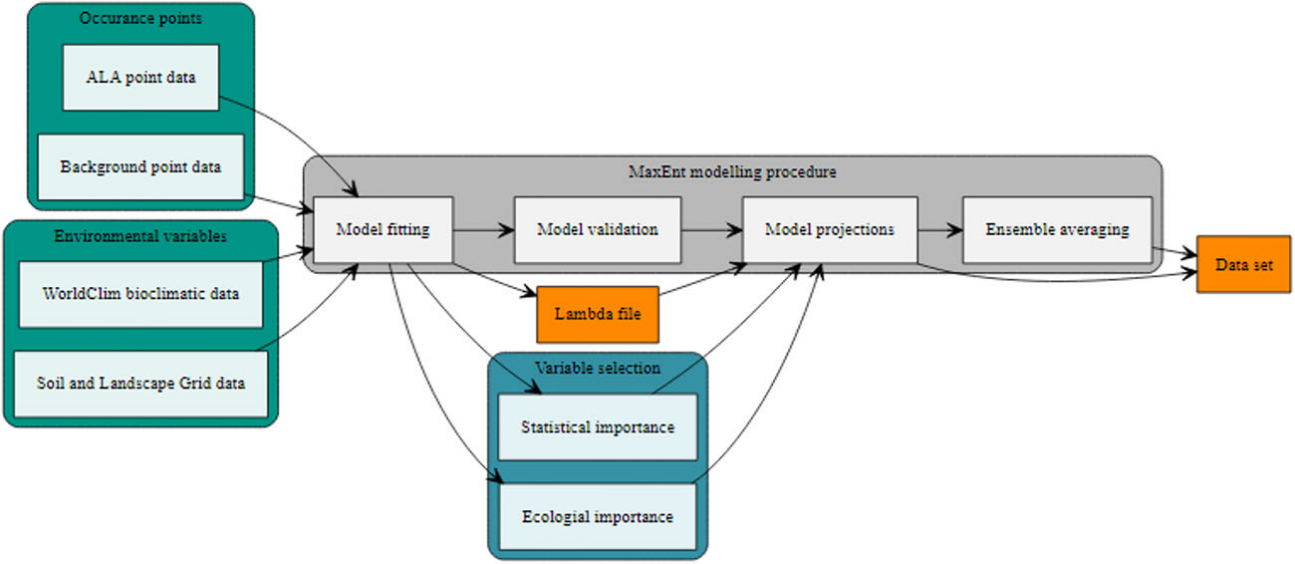
Workflow of the MaxEnt modeling procedure. Input data are represented as green, variable selection procedure is represented as purple, MaxEnt modeling procedure is represented as gray, and the output files are represented as orange.

The workflow for this study was adapted from the CliMAS project [16] (Fig. 1). The first step involved compiling and collecting the input data, which consisted of occurrence point data and environmental variables. We then used MaxEnt to fit models of habitat suitability using climate, soil, and landscape variables. We used a variable selection procedure, which considered the statistical and ecological importance of variables to refine the predictor variables as well as validating the models. We then used the lambda files produced in the model-fitting step to project species habitat suitability under future climate scenarios.

### Input data

#### Species occurrence points

**Figure 2:**
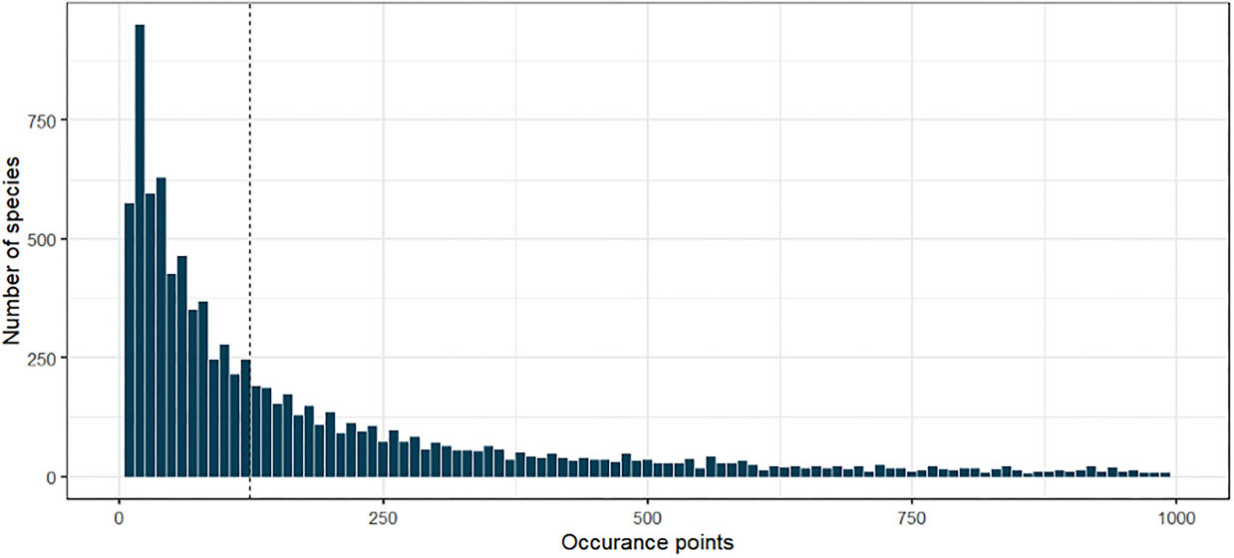
Distribution of occurrence points (*n*) for species models.

Species occurrence records, which were used to fit the historical climate models, were sourced from the Australian Atlas of Living Australia (ALA) [21], the Queensland Museum, and CSIRO. Vascular plant occurrence point data were acquired from the Queensland Museum. Vertebrate species occurrence were records acquired through ALA went through an additional data-cleaning process prior to modeling [21]. We used the points originally applied in the CliMAS project as of 2012 for vertebrates and the vascular plant points that were compiled for the CliMAS project but never modeled. Through these sources, we obtained occurrence point data for 197 mammals (60% coverage), 523 birds (71% coverage), 530 reptiles (50%), 191 amphibians (77%), and 9,200 vascular plants (44% coverage). Across all species, the median number of occurrence points was 123 and the distribution of the number of occurrence points ranged based on the following quantiles: 0% = 1, 25% = 43, 50% = 123, 75% = 410, 100% = 78,503 (Fig. 2).

MaxEnt uses background sample points as pseudoabsences and recommends the use of target groups in sample selection to help overcome potential spatial biases [22, 23]. To create the target group background files, we combined all occurrence points for all species within a taxonomic group and sampled the background points from this space. Each target group background file contained between 60,000 and 250,000 points depending on the taxonomic group, in which MaxEnt takes a subsample of 10,000 points.

#### Environmental variables

We used a combination of bioclimatic, soil, and landscape variables as predictors to fit the MaxEnt models. For the climate variables, we downloaded spatial data at a 5 km^2^ resolution on historical and future CMIP6 modeled bioclimatic variables through the WorldClim database [24]. Bioclimatic variables summarize monthly temperature and rainfall values into 19 more biologically meaningful variables (Table 1). Bioclimatic variables were downloaded for 8 GCMs: BCC-CSM2-MR [25], CNRM-CM6-1 [26], CNRM-ESM2-1 [27], CanESM5 [28], IPSL-CM6A-LR [ 29], MIROC-ES2L [30], MIROC6 [31], and MRI-ESM2-0 [32], for 4 shared socioeconomic (SSP) [33] and representative concentration pathway (RCP) combinations, RCP2.6-SSP1, RCP4.5-SSP2, RCP7.0-SSP3, and RCP8.5-SSP5, and 5 time periods (1990, 2030, 2050, 2070, and 2090). As we did not have access to the following 2 files, IPSL-CM6A-LR SSP2-4.5 2030 and MRI-ESM2-0 SSP5-8.5 2030, we linearly interpolated values. All climate scenarios and bioclimatic variables were clipped to the extent of Australia prior to modeling.

**Table 1:** Summary of the bioclimatic, soil, and landscape variable selected in the final MaxEnt model

Code	Variable name	Contribution^1^	Importance^2^	Ecological rationale
***Bioclimatic variables***				
BIO1	Annual Mean Temperature	8.72	18.21	Influences thermal tolerances of species.
BIO5	Max Temperature of Warmest Month	6.33	9.92	Influences upper thermal tolerances of species through extreme temperatures.
BIO6	Min Temperature of Coldest Month	4.30	8.66	Influences lower thermal tolerances of species through extreme temperatures.
BIO12	Annual Precipitation	8.60	10.81	Average annual rainfall, which influences water availability.
BIO13	Precipitation of Wettest Month	17.67	7.77	Maximum rainfall in the wettest month, which influences maximum water availability.
BIO14	Precipitation of Driest Month	14.93	8.45	Minimum rainfall in the driest month, which influences minimum water availability.
BIO15	Precipitation Seasonality	12.13	13.20	Standard deviation of rainfall in the annually, which influences the variation in water availability.
***Soil and landscape variables***				
AWC	Available Water Capacity	0.94	0.68	The amount of water held by the soil for future use.
BDW	Bulk Density (Whole Earth)	0.89	1.17	Soil’s ability to function for structural support, water and nutrient and microbial life movement, and soil aeration.
CLY	Clay	1.04	0.95	Promotes water retention and reduces air circulation in soil.
DES	Depth of Soil	2.00	1.29	Defines the root space and volume of soils available.
ECE	Electroconductivity	3.39	5.21	Movement of nutrients within the soil, which influences the availability of soil nutrients.
elev	Elevation	2.37	1.57	Elevation influences soil properties and air pressure.
pHc	pH	5.43	4.30	Affects the amount of nutrients that are water soluble in soil.
slope	Slope Relief	1.81	1.00	Influences soil properties and creates varying microclimates.
SLT	Silt	2.63	2.10	Promotes water retention and creates relatively porous soil conditions.
SND	Sand	1.60	1.60	Promotes water drainage and air circulation in soil.
SOC	Organic Carbon	5.17	3.05	Promotes soil structure by providing a food source for micro-organisms.

1 Average (mean) percent contribution in the final models for each environmental variable across all species. A measure of the contribution of each variable toward model fit after each iteration of the MaxEnt model.

2 Average (mean) percent importance in the final models for each environmental variable across all species. A measure of the importance of each variable measure depends the resulting decrease in training AUC on the final MaxEnt model.

We downloaded 15 environmental variables from the Soil and Landscape Grid of Australia [34] to use as environmental predictors of habitat suitability. Additionally, we downloaded the Interim Biogeographic Regionalisation for Australia [35] (IBRA) as an indication of the inherent spatial differences in biome across Australia. Soil and landscape variables were clipped and masked to the extent of Australia and scaled to the same resolution as the bioclimatic data (Table 1).

### MaxEnt modeling procedure

#### Model fitting

All habitat suitability models were fit in MaxEnt Version 3.4.1 using the command line. MaxEnt models were first run with 10 replicates (replicates = 10) validated using a cross-validation method to train the model and to compute model validation statistics. At this stage, habitat suitability values were calculated as values between 0 and 1 with no threshold applied and were later converted to values between 0 and 100. An example of the full MaxEnt model specification can be found in the GitHub or Zenodo repository affiliated with this article [36]. Important outputs of the MaxEnt modeling procedure include a .csv file containing statistical information to inform variable selection and model validation as well as the “lambdas file,” which is a text file containing the regression coefficients or lambdas fit by MaxEnt during modeling.

#### Variable selection

The variables included in the final MaxEnt model runs were informed by analyzing the variable contributions and importance percentages calculated using a full MaxEnt model run, information about variable complexity [37], and ecological knowledge based on several published models of terrestrial vertebrate and vascular plant climate and habitat suitability. The goal of variable selection was to reduce the number of predictor variables from the initial 35 variables chosen as potential environmental predictors to avoid overfitting. Although MaxEnt is robust to multicollinearity among variables [38], including excessive numbers of predictors can affect the model’s ability to make inferences outside of the training data.

We reviewed variables included within several Australian biodiversity modeling efforts of terrestrial vertebrates [16] and vascular plants [39, 40]. We then performed a full MaxEnt model run, which included the 35 variables described in the above section, for each species. We reviewed the importance of variables based on the average percent contribution and percent importance values across all species. The percent contribution is a measure of the contribution of each variable toward model fit after each iteration of the MaxEnt model, while the percent importance is a measure of the importance of each variable toward model fit for the final MaxEnt model. We also categorized bioclimatic variables based on complexity and favored simple variables as they tended to be less correlated with one another [37].

This combined approach to variable selection resulted in 18 variables that moved through to the model-fitting stage (Table 1): 7 bioclimatic variables and 11 soil and landscape variables. All bioclimatic variables selected for this study were included in CliMAS models [16] and similar modeling efforts for Australian plants [39], and all bioclimatic variables with the exception of BIO15 were considered simple climate variables [37] (Table 1). All bioclimatic variables except for BIO05 had high or moderate importance values in the full model. Similarly, we included additional soil and landscape variables [41] based on their use in recent biodiversity models [39], and we favored soil and landscape variables that were simpler.

#### Model validation

Once variables were selected, models were rerun, and model performance was assessed based on the area under the curve (AUC; i.e., the area under the receiver operating curve [ROC] curve) and the Boyce Index. The AUC is a widely used model validation metric used within the MaxEnt literature [42]. The AUC metric measures the predictive accuracy of the model and represents the probability that a randomly selected occurrence point is ranked higher than a randomly selected background point. The Boyce Index is another method that can be used to evaluate model performance and does so by assessing the magnitude in which the model predictions differ from random distribution of the observed presences across the prediction gradients [43, 44]. The Boyce Index value is represented by the Spearman rank correlation coefficient, which assesses the increase in the prediction/expected (P/E) plot [45].

**Figure 3:**
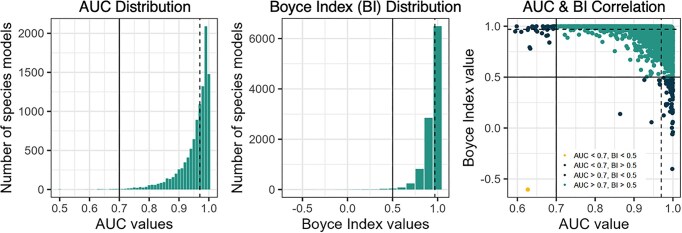
From left to right, the plots are the distribution of AUC values, the distribution of Boyce Index (BI) values, and a scatterplot between AUC and BI values for species models. The median AUC and Boyce Index value is represented by a dashed line. On the AUC plot, the 0.7 threshold is presented using a solid vertical line. On the BI plot, the 0.5 threshold is presented using a solid vertical line. These thresholds are also represented by solid lines on the scatterplot.

The median AUC across all models was 0.97, and generally, AUC values of 0.7 or below indicate poor performance (Fig. 3). We assess that 99.6% (*n* = 10,566) of species have an AUC value above 0.7 AUC, and 0.4% (*n* = 38) of species have an AUC value below the 0.7 threshold (33 birds, 4 vascular plants, and 1 mammal). Boyce Index values can vary from −1 to 1, and we find that the median Boyce Index across all models in this study was 0.97 (Fig. 3). A Boyce Index closer to 1 indicates that suitability predictions are consistent with the occurrence point distribution, and values of 0.5 or below generally indicate poor performance [43, 44]. We assess that 99.3% (*n* = 10,509) of species have a value over 0.5, 0.65% (*n* = 69) species had a value between 0.5 and 0, and 0.05% (*n* = 5) species had a value below 0 (1 bird and 4 vascular plants).

We have also provided a scatterplot summary of AUC in relation to the Boyce Index. Based on the 0.7 threshold for AUC and the 0.5 threshold for the Boyce Index, we find that 98.99% of species meet both thresholds. We find that 0.69% (*n* = 73) meet the AUC threshold but not the Boyce Index threshold, 0.32% (*n* = 34) species meet the Boyce Index threshold but not the AUC threshold, and 1 species did not meet either threshold (brown falcon, *Falco berigora*). Prior to using species data, please ensure you check the AUC and the Boyce Index value, which are contained within the species folder within the maxentResults.csv and the boyce_index_score.csv file.

#### Model projections

Using the best model selected in the model-fitting procedure, we projected species-level MaxEnt models under the future climate scenarios (RCP2.6-SSP1, RCP4.5-SSP2, RCP7.0-SSP3, and RCP8.5-SSP5) and 8 GCMs for 1 historical time period (1985) and 4 future time periods (2030, 2050, 2070, 2090) using the lambda files produced in the model-fitting step. Using the predicted habitat suitability data, we then calculated an ensemble average (mean), minimum, and maximum habitat suitability (to capture model variance) across 8 GCMs for each species, climate scenario, and time period.

#### Geospatial calculations

To describe the patterns of habitat suitability across time in an accessible tabular format, we calculated the total quality-weighted sum of habitat suitability for each species under different climate scenarios at each time period (Equation (1)). We first adjusted the resolution of the rasters to 1 km^2^; therefore, the quality-weighted habitat area (*qwHA*) sum corresponds to the “habitat area” in km^2^. For example, if the probable habitat suitability in a cell is equal to 1, the cell is equivalent to 1 km^2^, whereas if the probable habitat suitability in a cell is equal to 0.3, the cell is equivalent to 0.3 km^2^. It should be noted that the quality-weighted habitat area is not equivalent to the realized area available for a species given ecological or land-use constraints, which can both influence habitat availability and suitability for species. The probability of habitat suitability (*p*) was summed across raster cells (*xy*) for each species (*j*), year (*y*), and climate scenario (*c*):


(1) \begin{eqnarray*}
qwH{A}_{jyc} = \mathop \sum \limits_{i = 1}^n {p}_{jyc,xy}
\end{eqnarray*}

To describe how the patterns of habitat suitability may have changed across space under different climate scenarios or years, we summarized raster data for each species in multiple ways. For each taxonomic group (*t*), we calculated changes in habitat suitability (*s*) by subtracting future time periods and climate scenarios (*yc*) by historical climate niche (${p}^h$), where positive values indicate areas that increase in suitability in the future and negative values indicate areas that decrease in climate suitability in the future. We provide visual representation of this information in Fig. 7 and include the absolute and proportional change in habitat area in the tabular summaries provided for species:


(2) \begin{eqnarray*}
S{_t^{yc}} = p_t^h - p_t^{yc}
\end{eqnarray*}

To spatially identify important areas of climate refugia, which was done for Fig. 6, we multiplied the historical habitat suitability matrix by the habitat suitability in each future climate scenario and year combination. For each the cell, the probability of habitat suitability values per cell (*p*), for each species (*t*), year (*y*), and climate scenario (*c*), was multiplied by the future habitat suitability. Cell values were then divided by 100, and the resulting cell value represents climate refugia (*r*) between 0 and 100.


(3) \begin{eqnarray*}
r{_t^{yc}}_{\mathrm{\ }} = (p_t^h * p_t^{yc})/100
\end{eqnarray*}

### Reuse potential

#### Code availability

For each species, MaxEnt models were run directly from the terminal using java and bash syntax and were ultimately executed using a “Simple Linux Utility for Resource Management” (SLURM) workload manager on a high-performance Linux-based computer cluster. Additional modeling and geospatial analyses were processed using a shell file executed using SLURM on the computer cluster. The scripts used to generate these data are available in the companion GitHub and Zonodo repositories [36].

#### Dataset

Individual species’ maps for historical and future minimum, mean, and maximum ensembled habitat suitability, as well as the MaxEnt lambda file and summary reports produced in this study, are publicly accessible for download on the open-access companion GigaDB database [46, 77]. This dataset includes species-level historical (1970–2000 centered on 1990) and the future minimum, mean, and maximum habitat suitability projections for 1,382 terrestrial vertebrates (182 amphibians, 487 birds, 178 mammals, and 535 reptiles) and 9,251 vascular plants under 4 climate scenarios and 5 time periods; these data equate to 521,017 .tif raster files that are compressed using Lempel–Ziv–Welch (lzw) compression. Additionally, for each species, we have included a .csv file that contains the total quality-weighted habitat areas (in km^2^) for each species under each different climate scenario and time period. We have also consolidated these tables across all species and included these tabular data. A complete list of the species for which habitat suitability maps were produced can be found in the companion GigaDB database.

#### Spatial resolution of data

**Figure 4:**
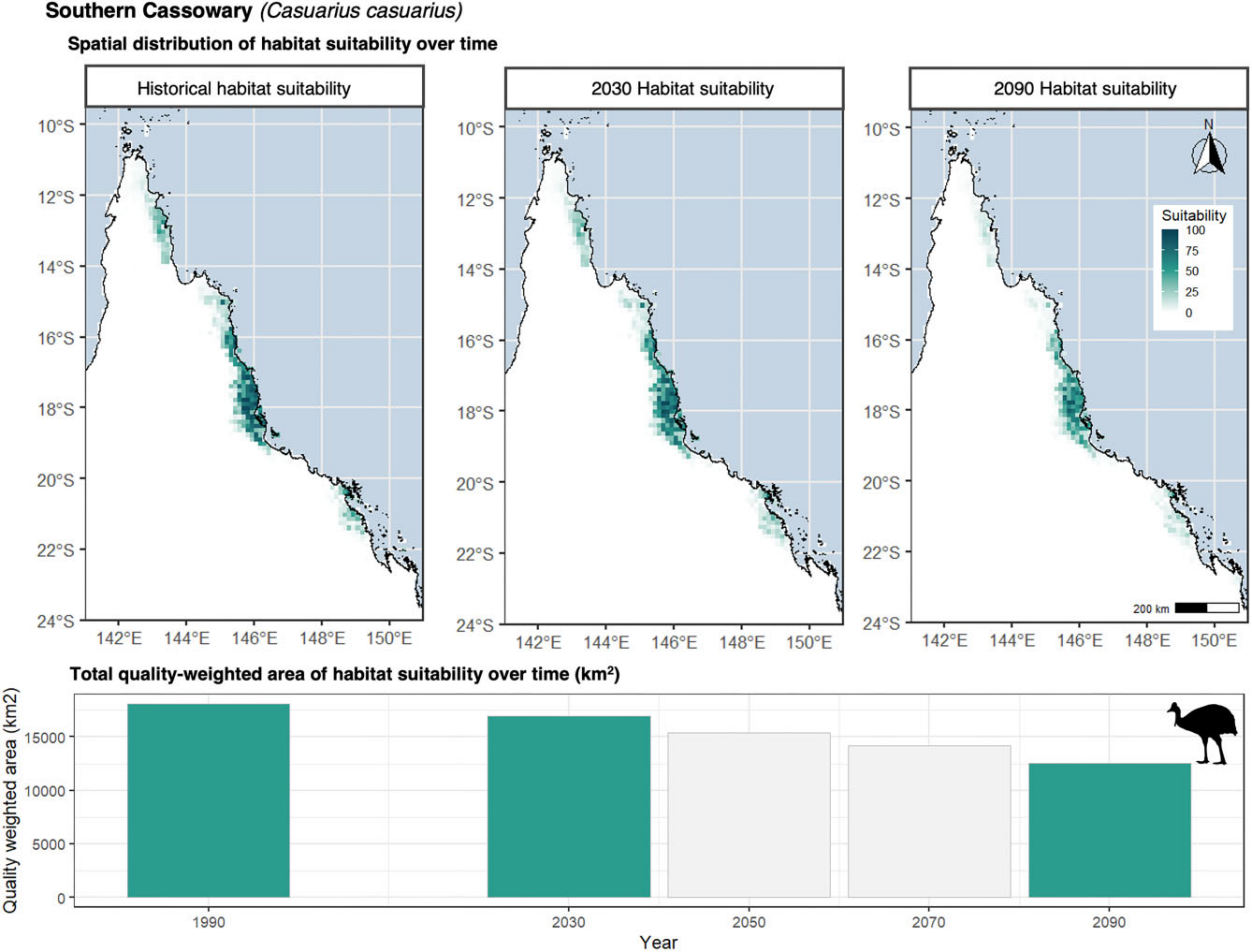
This habitat suitability distribution is for the southern cassowary (*Casuarius casuarius*) and presents its historical suitability projection historically, in 2030, and in 2090. The graph represents the total amount of habitat suitability (km^2^) available in each time period; green bars correspond to the maps presented (historical, 2030, and 2090). *Casuarius casuarius* icon was sourced from T. Michael Keesey, PhyloPic (2014 May 30).

These data are presented at a 5-km^2^ resolution, which is aligned with the climate data used as key inputs to the MaxEnt model. The data can be subsequently downscaled to finer resolutions, but assumptions will have to be made about how habitat suitability is distributed across cells. The current resolution of these data is best utilized to understand general trends across space and time. To demonstrate the resolution, we present the southern cassowary (*Casuarius casuarius*), which is known to occur in the Wet Tropics region of Queensland, Australia. Current suitable areas for the southern cassowary are predicted to occur between Townsville and Cooktown, with an isolated area around the Iron Range (Fig. 4). Taking the most severe climate change scenario (RCP8.5-SSP5), the environmental space for the southern cassowary is predicted to reduce over time around its central habitat in the Atherton Tablelands. The maps for the southern cassowary can be compared with [16] for reference.

#### Species-level data summary

**Figure 5:**
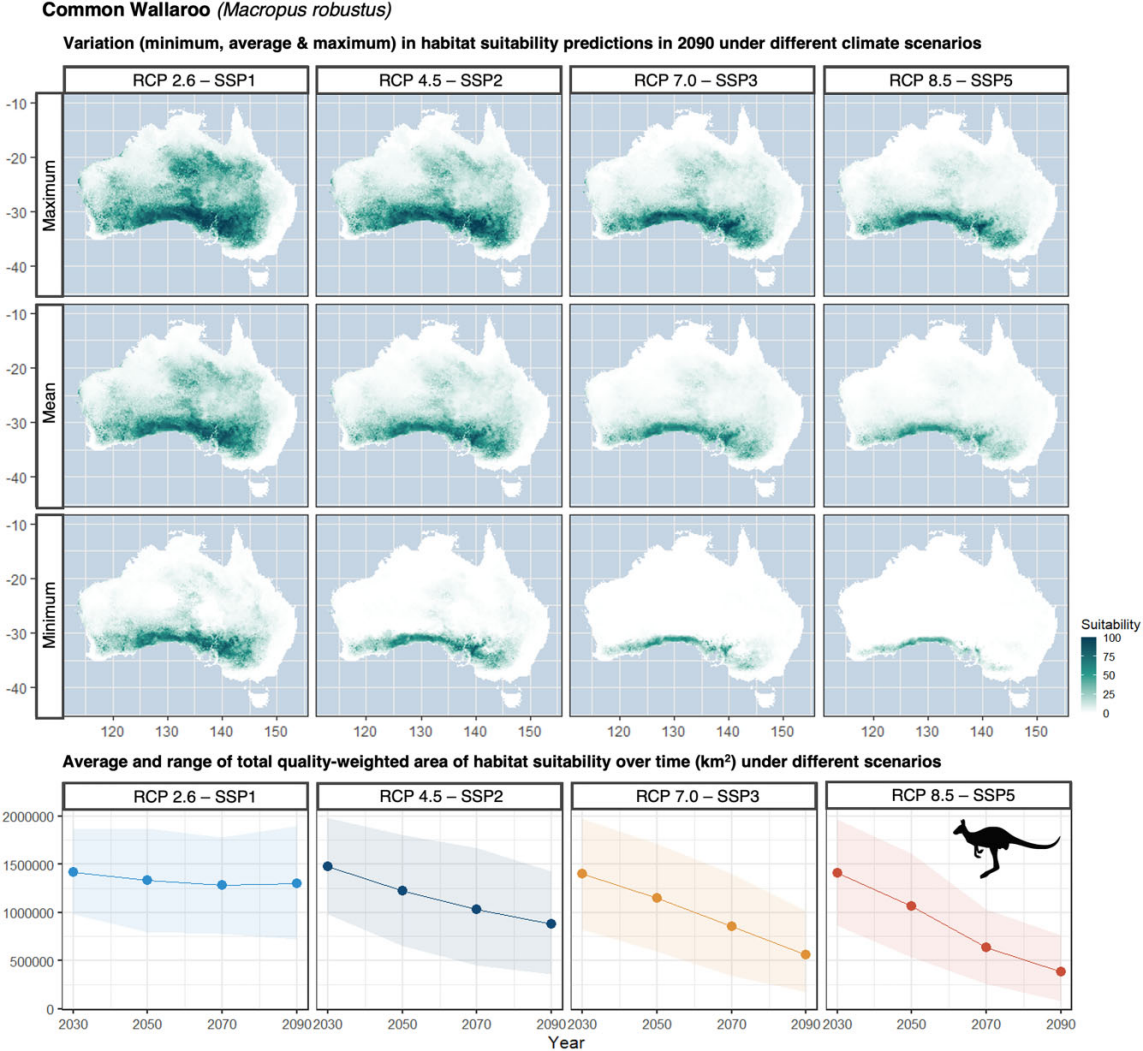
These habitat suitability distributions are for the common wallaroo (*Macropus robustus*) for 1 historical projection and 4 future emission scenarios in the year 2090. The habitat suitability distributions of each row of maps represent the minimum, mean, and maximum habitat suitability projections across GCMs. The line graphs represent the total habitat suitability (km^2^) for 4 future emission scenarios over time. The uncertainty band represents the minimum and maximum amount of habitat suitability across GCMs. *Macropus robustus* icon sourced from Jiekun He, PhyloPic (2022 Apr 10).

The dataset includes suitability maps for species under different climate scenarios and time periods using an ensemble average approach. Through the process of ensemble averaging, the minimum and maximum suitability maps were also produced. These maps can be compared to understand the bounds of how climate change may generally impact habitat suitability in the future. The importance of incorporating multiple GCM projections can be seen by the variation among the minimum, mean, and maximum suitability maps (Fig. 5). For the common wallaroo (*Macropus robustus*), the differences between the minimum, mean, and maximum suitability maps are most apparent under worsening climate scenarios. Areas across the southern parts of Australia remain suitable across all 3 suitability maps, compared to areas in the central and northern parts of their range becoming progressively less suitable. These trends are consistent with other macropod modeling studies that also suggest suitability for the common wallaroo will track south as climate scenarios worsen [47]. The maps for the common wallaroo can also be compared with [16] for reference.

#### Spatial changes over time

**Figure 6:**
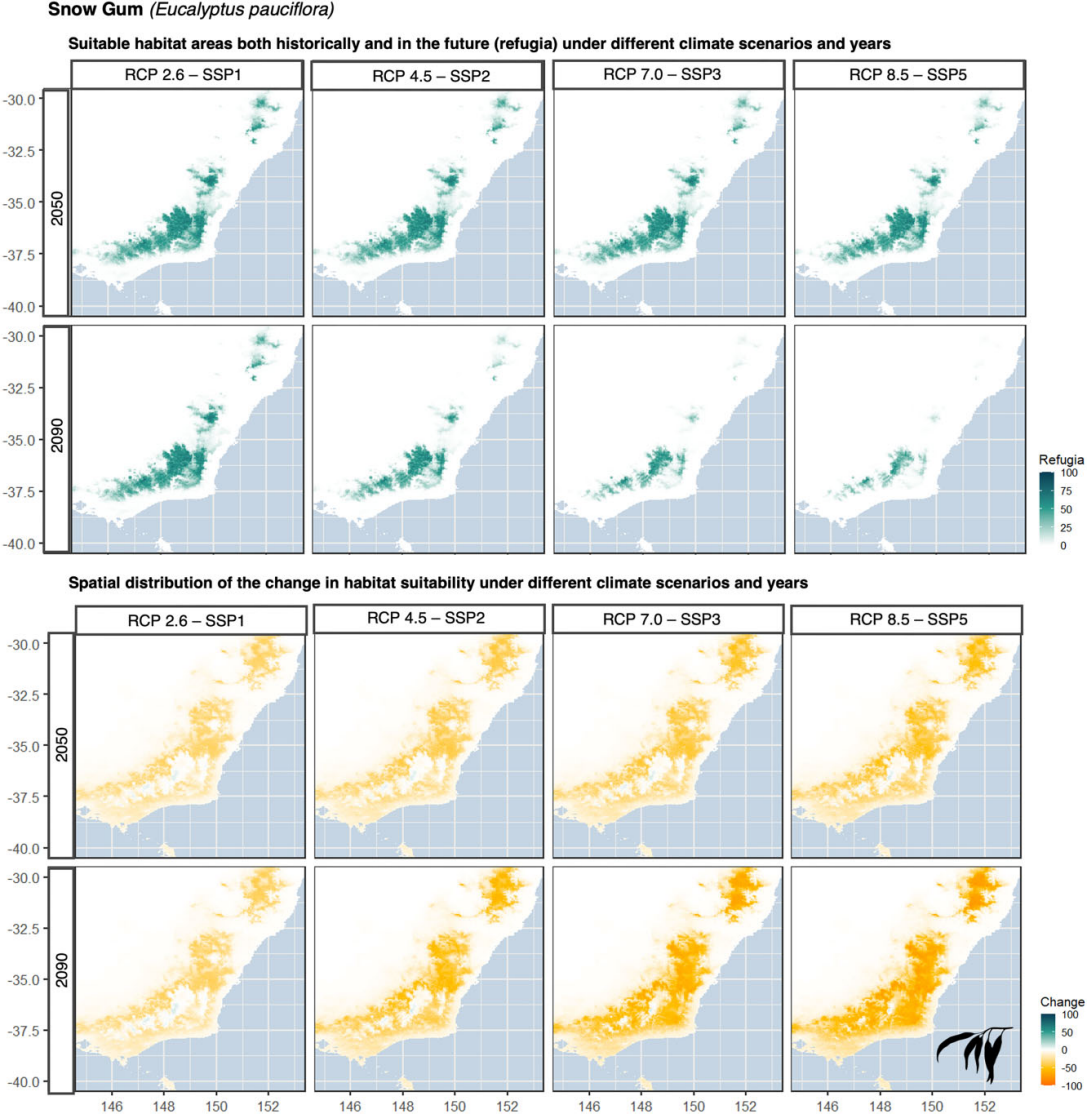
Refugia and habitat suitability change maps for snow gum (*Eucalyptus pauciflora*). The top panel presents climate change refugia for 4 future emission scenarios in the years 2050 and 2950. Dark green on the refugia maps represents areas that have high predictive suitability historically as well in future time periods. The bottom panel presents changes in habitat suitability for 4 future emission scenarios in the years 2050 and 2090. Orange areas indicate places that decrease in suitability compared to the previous time period, and green areas indicate areas that improve in suitability. White areas indicate no change in suitability. Eucalyptus leaf icon sourced from Ferran Sayol, PhyloPic (2019 Oct 9).

Taking this a step further, geospatial calculations can also be applied to determine the differences between years or climate scenarios. This can be conducted to identify areas of refugia (Equation (3)) or the location and magnitude of change between different time periods (Equation (2)). To calculate spatial locations of refugia, historical and future suitability maps can be multiplied together to accentuate areas in space that are suitable in both time periods. To calculate spatial changes in habitat suitability through time, historical suitability maps can be subtracted from future suitability maps to spatially accentuate locations that have changed in habitat suitability (i.e., improved in suitability or declined in suitability) across time periods. Using the snow gum (*Eucalyptus pauciflora*) as an example, we find refugia in the alpine region of Australia is predicted to decline for the snow gum under worsening climate scenarios, with declines being most severe in the year 2090 (Fig. 6, top). Across all climate scenarios, habitat suitability is declining from all areas of the snow gum’s range, and we did not identify areas of increases (Fig. 6, bottom).

#### Changes in quality-weighted habitat area

**Figure 7:**
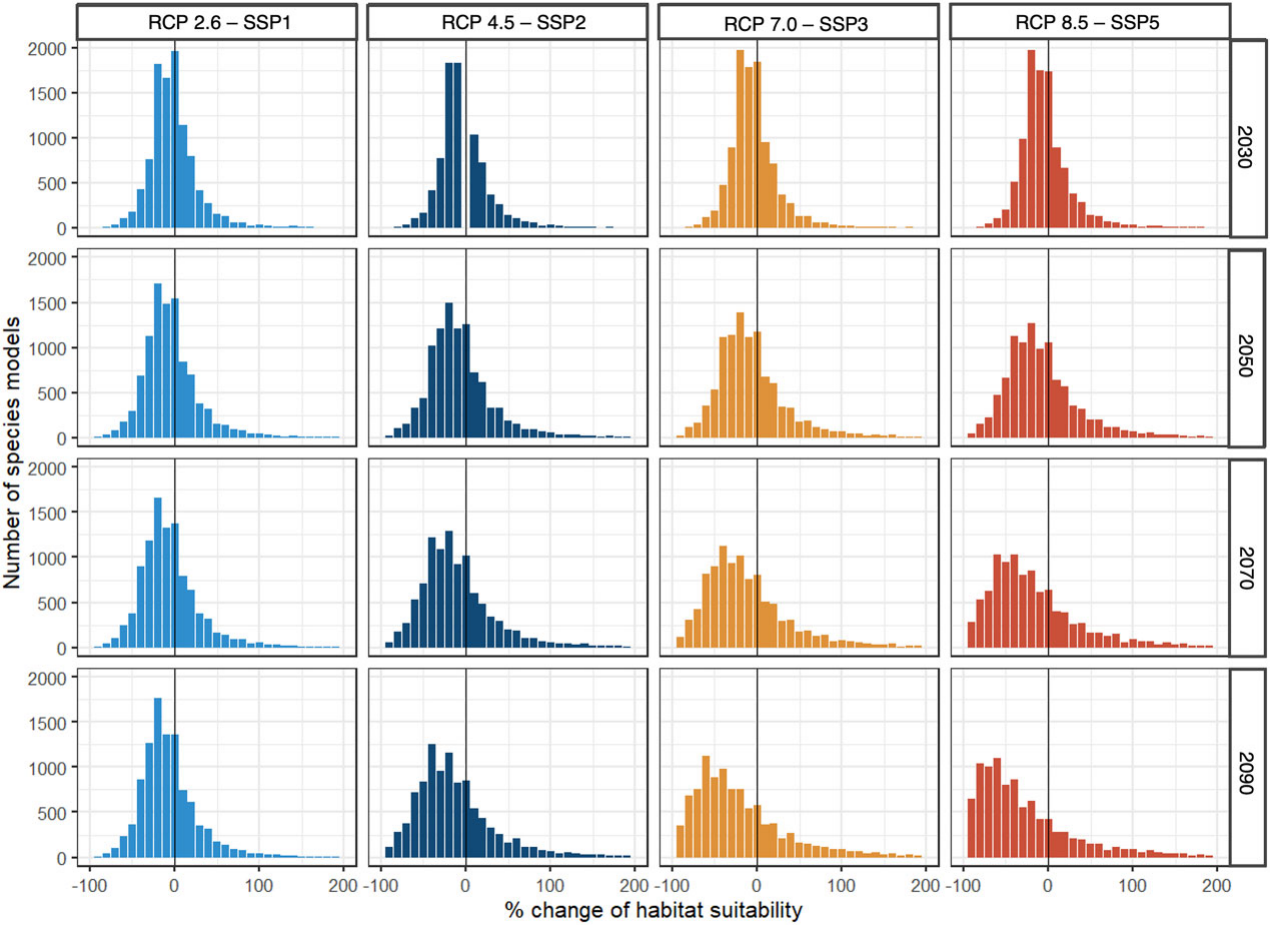
Histogram of the number of species and their relative change in quality-weighted habitat area between 1990 and each future time period (2030, 2050, 2070, 2090).

The dataset also includes a tabular summary of quality-weighted habitat area in km^2^ for each species under different climate scenarios and time periods (Equations (1) and (2)). The quality-weighted habitat area values can be analyzed and plotted to understand how climate change may impact habitat area for single species or groups of species in the future (Fig. 7). When these data are summarized across all species, we can show that in 2030, the distribution of change in habitat area is similar across the 4 climate scenarios. However, in 2090, the distribution of change in habitat area follows a different pattern across climate scenarios with progressively more species losing progressively more habitat area as climate change worsens (Fig. 7).

## Discussion

Spatial data on the suitability of areas for species are an important input to guide conservation planning, policy ,and management. The objective of this article was to develop habitat suitability maps for Australian flora and fauna under different climate futures using a MaxEnt approach. These data have been developed in a way that is consistent across species and enables users to analyze how different climate futures may impact the habitat suitability for biodiversity more generally across Australia. These data can also be used for species-level analysis and can be a starting point for additional analyses that use either geospatial information or tabular information that could take into consideration additional information like land use, conservation actions, or species ecology.

### Applications for landscape and species conservation

This spatial and tabular dataset is ideal for users who would like to understand how the habitat suitability of areas for species is predicted to change over time or under different climate scenarios. For example, at the landscape level, these habitat suitability maps can be combined into a general biodiversity layer to evaluate how habitat suitability more generally changes over time (Fig. 7) or over space and time (Figs. 4–6) [48]. These data can then be utilized in applications such as spatial prioritizations using such tools as Zonation [49] or Marxan [50] to guide spatial conservation priorities in Australia [4, 19]. Therefore, using these data for subsequent analysis can be useful to inform conservation (e.g., where to establish new protected areas), restoration, or monitoring plans in areas that are suitable for biodiversity or are predicted to lose or gain suitable areas for biodiversity.

At the species level, this dataset can be used to support conservation actions for species of interest (e.g., threatened species, iconic species, endemic species). The tabular data can be used to systematically identify species of interest based on the way climate change is anticipated to impact the species or could be used to inform processes such as threatened species listing [51]. Spatial information about species could also be useful to compare the long-term suitability of areas for threatened species under climate change to inform present-day decision-making and species management [52, 53]. This could be paired with other types of data to assess the impacts of climate change on species [54] or could inform broader-scale biodiversity conservation analyses [55].

#### Applications in sustainability and natural capital accounting

Biodiversity forms a foundation of broader sustainability ideals; therefore, to measure progress toward sustainability, conservation or corporate goals spatial data on biodiversity can serve as an important input information to the creation of metrics [56, 57]. Biodiversity indicators like the species richness, or more complex indicators like the Species Threat Abatement and Restoration metric (STAR) [58] or the biodiversity intactness index (BII) [59], all draw from species layers as input data. Feeding the habitat suitability maps generated in this study into biodiversity layers and into broader sustainability models or assessments can improve the consideration of biodiversity against other environmental or social values. This may include initiatives such as land-use planning or land-use change modeling [60, 61].

Additionally, as many businesses are transitioning toward “nature positive,” the use of biodiversity to monitor business impacts and progress toward nature positive is necessary. The habitat suitability maps generated in this study can be used to represent key species or biodiversity within natural capital within frameworks such as the System of Environmental-Economic Accounts (SEEA) framework [62], within sustainability assessments such as “foot printing” to enhance the biodiversity input data [63–65], or within nature-related impact or dependency assessments that inform frameworks like the Taskforce on Nature-Related Financial Disclosures (TNFD) [66].

#### Limitations and caveats with the data

When using and interpreting the data contained in this dataset, it is important to consider the following limitations and considerations. This dataset presents the habitat suitability of areas for species under different climate scenarios and time periods using a correlative approach. These maps are not distribution maps; rather, they present habitat suitability based on climate, soil, and landscape characteristics. Due to its 5-km^2^ spatial resolution, the data are best for understanding broader spatial trends that can be integrated into spatial planning [19], rather than more local management such as identifying specific sites for translocation without additional finer detail [54]. These maps have not been thresholded, nor do they consider dispersal [16], land use [67], biophysical capacity [68], or attributes that may be important for species of interest (e.g., normalized difference vegetation index [NDVI], fire or vegetation structure) [54]. There are a multitude of other methods to model suitability and species distributions that have their own use cases and limitations [69, 70].

The occurrence points used for this analysis were those originally used for the CliMAS work, and the ALA data were passed through an additional rigorous cleaning process for terrestrial vertebrates only. This process helped reduce the spatial bias and noise in the occurrence points [23]; however, more broadly, there are sampling biases that influence the distribution of occurrence points, such as land tenure. To improve on the models, an integrated pathway to ALA into the modeling procedure would be ideal as this would ensure up-to-date input data. However, this can also come with challenges as occurrence data are required to have the same temporal resolution to the historical or current climate data (i.e., 1990 in this study). While we did use target background files to reduce spatial biases [22], there may still be limitations of this approach at the taxonomic group level, for example, for small-ranging species [71]. Taxonomic-level grouping may still be too broad to adequately capture those species that are highly range restricted and require very specific micro-climate needs; therefore, species-specific level grouping may help to overcome this. Background files that are too broad may adequately capture sampling biases or the true relationship between occurrence points and environmental predictors.

MaxEnt models are also prone to overfit but are also less influenced by collinearity than statistical models; we tried mitigating the impacts of overfitting the MaxEnt models by conducting variable selection. In relation to the variables used, we were primarily guided by past efforts that model the suitability of areas across Australia for many species [39, 40], but this approach obviously overlooks some variables that can be important to suitability. For example, we did not consider variables such as the NDVI [72], land use [73], weather [74], or detailed information about vegetation structure or extreme events like fire [54]. Thus, our recommendation is for the users of these data to consider whether the variables used to model habitat suitability in this study are compatible with the species of interest or whether additional information is required. This will likely be the case if the user is interested in a more fine-scale application of the data, for example, at the single species or local level, as these data are best suited for macro-level analyses and applications.

Finally, there is much contention around the best way to assess model performance of MaxEnt models beyond just the AUC, to approaches like the True Skill Statistic value, the kappa score, and the Boyce Index [44, 45, 75, 76]. We present the AUC and the Boyce Index and do not consider the thresholds for these indexes prior to creating the habitat suitability projections; therefore, the user can assess the model performance for their species on interest when interpreting the data.

## Conclusion

To spatially target conservation actions, spatial information about the location and suitability of areas for species is needed. This study provides a comprehensive dataset of predicted habitat suitability under 4 climate futures while also incorporating the uncertainty across GCMs. We are providing a spatial and tabular data product at the Australian scale and at 5-km^2^ resolution that can be used to inform research and decision-making at local, regional, and national scales. These data can be applied within strategic conservation planning approaches and used to identify important areas for species consecration [5]. Spatial information about current and future suitable areas for species is a key component of conservation planning, particularly as the impact of climate change on species and biodiversity is uncertain.

## Data Availability

All spatial and tabular data are freely accessible in the *GigaScience* repository, GigaDB [77].

## Abbreviations

ALA: Atlas of Living Australia; AUC: area under the curve; BI: Boyce Index; BII: Biodiversity Intactness Index; CanESM5: Canadian Earth System Model, version 5; CMIP3: Coupled Model Intercomparison Project 3; CMIP6: Coupled Model Intercomparison Project 6; CSIRO: Commonwealth Scientific and Industrial Research Organisation; csv: comma-separated values file; GCM: general circulation model; IBRA: Interim Biogeographic Regionalisation for Australia; IPCC: Intergovernmental Panel on Climate Change; IPSL-CM6A-LR: Institut Pierre-Simon Laplace climate model; lzw compression: Lempel–Ziv–Welch compression; MIROC-ES2L: Model for Interdisciplinary Research on Climate, Earth System version 2 for Long-term simulations; MIROC6: Model for Interdisciplinary Research on Climate 6; MRI-ESM2-0: Meteorological Research Institute Earth System Model; NDVI: Normalized Difference Vegetation Index; P/E plot: prediction/expected plot; qwHA: quality-weighted habitat area (in km ^2^); RCP: representative concentration pathway; ROC: area under the receiver operating curve; SEEA: System of Environmental-Economic Accounts framework; SLURM: Simple Linux Utility for Resource Management; SSP: shared socioeconomic pathway; STAR: Species Threat Abatement and Restoration metric; TNFD: Taskforce on Nature-Related Financial Disclosures.

## Competing Interests

The authors declarethat they have no competing interests.

## Funding

This work was made possible by generous philanthropic support for Climateworks Centre’s Land Use Futures program, which supported C.A and B.B.; C.A. was also supported by an Alfred Deakin Postdoctoral Research Fellowship 2023–2025; D.S. was partly funded by the 2021–2024 ARC Linkage Grant Innovation in agricultural sector Greenhouse Gas abatement in New South Wales, led by Prof. Jeff Connor.

## Authors' Contributions

C.A., D.S. and E.G.: data curation, methodlogy and writing – review & editing. C.A. and D.S.: formal analysis. E.G.: conceptualization and funding acquisition.

## Supplementary Material

giae002_GIGA-D-23-00183_Original_Submission

giae002_GIGA-D-23-00183_Revision_1

giae002_Response_to_Reviewer_Comments_Original_Submission

giae002_Reviewer_1_Report_Original_Submission Arshad Mahmood Khan, Ph.D. -- 8/13/2023

giae002_Reviewer_1_Report_Revision_1 Arshad Mahmood Khan, Ph.D. -- 12/6/2023

giae002_Reviewer_2_Report_Original_Submission Nabaz Khwarahm -- 8/26/2023

giae002_Reviewer_2_Report_Revision_1 Nabaz Khwarahm -- 12/4/2023

giae002_Reviewer_3_Report_Original_Submission Masoud Yousefi -- 9/7/2023
