# Correction to: Habitat suitability maps for Australian flora and fauna under CMIP6 climate scenarios

**DOI:** 10.1093/gigascience/giae031

**Published:** 2025-03-05

This is a correction to: Carla L Archibald, David M Summers, Erin M Graham, Brett A Bryan, Habitat suitability maps for Australian flora and fauna under CMIP6 climate scenarios, *GigaScience*, Volume 13, 2024, giae002, https://doi.org/10.1093/gigascience/giae002.

In the originally published version of this manuscript, a reference to the open-access companion GigaDB database was erroneously omitted from the ‘Dataset’ paragraph of the ‘Reuse potential’ section. This has been corrected.

Additionally, the metadata file giae002_GIGA-D-23-00183_Supplementary-material_GigaDBUploadForm has been removed from the supplementary material, as the details in this file are contained in the cited GigaDB database.

