# Supplementary material for: Habitat suitability maps for Australian flora and fauna under CMIP6 climate scenarios
Source: Gigascience. 2024 Mar 5;13:giae002. doi: 10.1093/gigascience/giae002 (PMC10939329; doi:10.1093/gigascience/giae002)
Supplement: giae002_GIGA-D-23-00183_Original_Submission [file giae002_giga-d-23-00183_original_submission.pdf]

GigaScience

Habitat suitability maps for Australian flora and fauna under CMIP6 climate scenarios

--Manuscript Draft--

**Manuscript Number:**	GIGA-D-23-00183
**Full Title:**	Habitat suitability maps for Australian flora and fauna under CMIP6 climate scenarios
**Article Type:**	Data Note
**Funding Information:**	
**Abstract:**	Spatial information about the location and suitability of areas for native plant and animal species under different climate futures is an important input to land use and conservation planning and management. Australia, renowned for its abundant species diversity and endemism, often relies on modelled data to assess species distributions due to the country's vast size and the challenges associated with conducting on-ground surveys on such a large scale. Modelled habitat suitability maps use information about known occurrences of species and predict suitable areas for species using climate, soil and landscape information. Using MaxEnt, we produced Australia-wide habitat suitability maps under RCP2.6-SSP1, RCP4.5-SSP2, RCP7.0-SSP3 and RCP8.5-SSP5 climate futures for 1,382 terrestrial vertebrates and 9,251 vascular plants at 5km2 for open access. This represents 60% of all Australian mammal species, 77% of amphibian species, 50% of reptile species, 71% of bird species and 44% of vascular plant species. We also include tabular data which includes summaries of total quality-weighted habitat area of species under different climate scenarios and time periods. These habitat suitability maps can be used as input data for landscape and conservation planning or species management, particularly under different climate change scenarios in Australia.
**Corresponding Author:**	Carla Leigh Archibald, Ph.D<br>Deakin University<br>Burwood, VIC AUSTRALIA
**Corresponding Author Secondary Information:**	
**Corresponding Author's Institution:**	Deakin University
**Corresponding Author's Secondary Institution:**	
**First Author:**	Carla Leigh Archibald, Ph.D
**First Author Secondary Information:**	
**Order of Authors:**	Carla Leigh Archibald, Ph.D
	David M Summers, Ph.D
	Erin M Graham, Ph.D
	Brett A Bryan, Ph.D
**Order of Authors Secondary Information:**	
**Additional Information:**	
**Question**	**Response**
Are you submitting this manuscript to a special series or article collection?	No
**Experimental design and statistics**<br><br>Full details of the experimental design and statistical methods used should be given	Yes

Powered by Editorial Manager® and ProduXion Manager® from Aries Systems Corporation

in the Methods section, as detailed in our Minimum Standards Reporting Checklist. Information essential to interpreting the data presented should be made available in the figure legends.<br><br>Have you included all the information requested in your manuscript?	
**Resources**<br><br>A description of all resources used, including antibodies, cell lines, animals and software tools, with enough information to allow them to be uniquely identified, should be included in the Methods section. Authors are strongly encouraged to cite Research Resource Identifiers (RRIDs) for antibodies, model organisms and tools, where possible.<br><br>Have you included the information requested as detailed in our Minimum Standards Reporting Checklist?	Yes
**Availability of data and materials**<br><br>All datasets and code on which the conclusions of the paper rely must be either included in your submission or deposited in publicly available repositories (where available and ethically appropriate), referencing such data using a unique identifier in the references and in the "Availability of Data and Materials" section of your manuscript.<br><br>Have you have met the above requirement as detailed in our Minimum Standards Reporting Checklist?	No
If not, please give reasons for any omissions below.<br><br>as follow-up to "**Availability of data and**	I am also intending to upload the data to GigaDB titled "Supporting data for "Habitat suitability maps for Australian flora and fauna under CMIP6 climate scenarios". Therefore, I have not provided the link to the GigaDB in the Data Note, but I intend to add this when possible. I have included an excel file in the Supplementary Material including the files names of each file. The GitHub page is available, and a link is

Powered by Editorial Manager® and ProduXion Manager® from Aries Systems Corporation

**materials**<br><br>All datasets and code on which the conclusions of the paper rely must be either included in your submission or deposited in publicly available repositories (where available and ethically appropriate), referencing such data using a unique identifier in the references and in the "Availability of Data and Materials" section of your manuscript.<br><br>Have you have met the above requirement as detailed in our Minimum Standards Reporting Checklist?<br><br>"	included in the manuscript.

Powered by Editorial Manager® and ProduXion Manager® from Aries Systems Corporation

Manuscript
Click here to access/download;Manuscript;Manuscript_Habitat-suitability-maps-under-climate-change.docx

Data Description

Introduction

Rich spatial and temporal information about the effect of climatic and environmental change on species distributions is necessary to ensure robust species management and conservation policy more broadly (Bryan et al., 2014; Hanson et al., 2019; Leclère et al., 2020; Summers et al., 2012). Identifying areas where species occur now, as well as areas which may be suitable in the future, is a crucial aspect of decision making under uncertainty (Summers et al., 2012). The availability of resources for conservation, including financial, staffing and land availability, is limited and exacerbates the challenge of conservation planning during climate change (Hanson et al., 2019). These constraints have sparked the need for more strategic landscape and conservation planning methods, such as spatial prioritisation, to identify the most effective conservation solutions (Tulloch et al., 2015). Spatial information on where species are now and where suitable areas may be in the future is the foundation of efficient planning for conservation action, particularly in areas where local conditions are more sensitive to climate change (Summers et al., 2012).

Australia is a hyper-diverse country with high levels of species endemism (Chapman, 2009; Coleman, 2016). Unfortunately, Australia also has some of the highest recorded numbers of contemporary extinctions worldwide and more than 1900 species and ecological communities are even now under threat (Woinarski et al., 2019; Australian Government Department of Agriculture and the Environment, 2021). Given the extensive and severe range and population declines of many threatened species (Bergstrom et al., 2021; Kearney et al., 2018; Woinarski et al., 2019), many more species are also predicted to have a high risk of extinction in the future (Garnett et al., 2022). To ensure the conservation of Australia's unique biodiversity, identifying and protecting important areas for species such as climate refugia is key to planning for resilience and adaptive capacity (Reside et al., 2014). To fulfill this task, underlying data

on species location and the habitat suitability of areas for species under different climate futures is required.

There are many ways to assess suitable areas for species, and one popular approach is to use the maximum entropy method (henceforth, MaxEnt). MaxEnt is a niche-based general-purpose machine learning method with a simple and precise mathematical formulation which is particularly well-suited for species distribution modelling with presence-only data (Phillips et al., 2006). Generating MaxEnt models for individual species at continental scales presents challenges around the processing and storage of large volumes of data. Graham et al. (2019) developed a comprehensive spatial dataset of 1,872 terrestrial and freshwater vertebrate species distributions using the Intergovernmental Panel on Climate Change's (IPCC) AR4 Coupled Model Intercomparison Project 3 (CMIP3) future climate projections (Meehl et al., 2007) and made them freely available through a web-based portal known as 'CliMAS'. Although the CliMAS models led to many applied outcomes (Maxwell et al., 2019; Ward et al., 2022), the website was retired in 2020, in recognition of the fact that there have been two major updates by the IPCC and the current projections are based on CMIP6. For conservation planning to progress, an improved and enlarged suite of freely available spatial data, based on up-to-date climate projections and extended for a much broader range of species including vascular plants, is needed.

We developed habitat suitability maps for Australian flora and fauna under different climate futures using a MaxEnt approach. We produced freely accessible Australia-wide habitat suitability maps for 1,441 terrestrial vertebrates and 9,251 vascular plants. This represents 60% of all Australian mammal species, 77% of amphibian species, 50% of reptile species, 71% of bird species and 44% of vascular plant species. We fit these models using 7 bioclimatic variables and 11 soil and landscape variables under 4 climate scenarios, 8 GCMs and 1 ensemble average, and 5 time periods. These habitat suitability maps are best used as input data to represent species or biodiversity values for conservation planning, particularly under different climate change scenarios in Australia.

Methods

The workflow for this study was adapted from the CliMAS project (Graham et al., 2019) (Figure 1). The first step involved compiling and collecting the input data which consisted of occurrence point data as well as climate, soil and landscape variables. We then used MaxEnt to fit models of habitat suitability using climate, soil and landscape variables. We conducted a variable selection procedure which considered the statistical and ecological importance of variables to refine the predictor variables as well as validating the models. We then used the lambda files produced in the model fitting step to project species habitat suitability under future climate scenarios and time periods.

[INSERT FIGURE 1]

Figure 1 *Workflow of the MaxEnt modelling procedure. Input data is represented as green, variable selection procedure is represented as purple, MaxEnt modelling procedure is represented as grey and the output files are represented as orange.*

Input data

Species occurrence points

Species occurrence records which were used to fit the historical climate models were sourced from the Australian Atlas of Living Australia (ALA) (Atlas of Living Australia, 2012), the Queensland Museum, and CSIRO. Vascular plant occurrence point data were acquired through from the Queensland Museum. Vertebrate species occurrence were records acquired through ALA went through an additional data cleaning process prior to modelling (see Graham et al., 2019). We used the points originally applied in the CliMAS project as of 2012 for vertebrates, and the vascular plant point compiled but never modelled with for the CliMAS project. Throughout these sources we obtained occurrence point data for 197 mammals (60% coverage), 523 birds (71% coverage), 530 reptiles (50%), 191 amphibians (77%) and 9,251 vascular plants (44% coverage). MaxEnt uses background sample points as pseudoabsences and recommends the use of target groups in sample selection (Philips *et al.* 2009). Each background file contained between

60,000 to 250,000 points depending on the taxonomic group, in which MaxEnt takes a subsample of 10,000 points.

Environmental variables

We used a combination of bioclimatic, soil and landscape variables as predictors to fit the MaxEnt models. For the climate variables, we downloaded spatial data at a 5km^2 resolution on historical and future CMIP6 modelled bioclimatic variables through the WorldClim database (www.worldclim.org, accessed on September 2020). Bioclimatic variables summarise monthly temperature and rainfall values into 19 more biologically meaningful variables. Bioclimatic variables were downloaded for eight global climate models (GCMs): BCC-CSM2-MR, CNRM-CM6-1, CNRM-ESM2-1, CanESM5, GFDL-ESM4, IPSL-CM6A-LR, MIROC-ES2L, MIROC6, MRI-ESM2-0, for four shared socioeconomic (SSP) and representative concentration pathway (RCP) combinations: RCP2.6-SSP1, RCP4.5-SSP2, RCP7.0-SSP3 and RCP8.5-SSP5 and 5 time-periods (1990, 2030, 2050, 2070 and 2090). As we did not have access to the following two files: IPSL-CM6A-LR SSP2-4.5 2030 and MRI-ESM2-0 SSP5-8.5 2030, we linearly interpolated values. All climate scenarios, bioclimatic variables were clipped to the extent of Australia prior to modelling.

We downloaded 15 environmental variables from the Soil and Landscape Grid of Australia database (https://www.csiro.au/en/research/natural-environment/land/soil-and-landscape-grid-of-australia, accessed on Sep 2021) to use as environmental predictors of habitat suitability. Additionally, we downloaded the Interim Biogeographic Regionalisation for Australia (IBRA) as an indication of the inherent spatial differences in biome across Australia. Soil and landscape variables were clipped and masked to the extent of Australia and scaled to the same resolution as the bioclimatic data.

MaxEnt modelling procedure

Model fitting

All habitat suitability models were fit in MaxEnt Version 3.4.1. Maxent models were first run with 10 replicates validated using a cross validation method to train the model and to compute model validation statistics. At this stage, habitat suitability values are calculated as values between 0 and 1 with no threshold applied and were later converted to values between 0 and 100. Important outputs of the MaxEnt modelling procedure include a .csv file containing statistical information to inform variable selection and model validation as well as the 'lambdas file', which is a text file containing the regression coefficients, or lambdas, fit by MaxEnt during modelling.

Variable selection

The variables included in the final MaxEnt model runs were informed by analysing the variable contributions and importance percentages calculated using a full MaxEnt model run, information about variable complexity (Low et al., 2021), as well as ecological knowledge based on several published models of terrestrial vertebrate and vascular plant climate and habitat suitability. The goal of variable selection was to reduce the number of predictor variables from the initial 35 variables chosen as potential environmental predictors to avoid overfitting. Although Maxent is considered to be robust to multicollinearity among variables (Feng et al., 2019), including excessive numbers of predictors can affect the model's ability to make inferences outside of the training data.

We reviewed variables included within several Australian biodiversity modelling efforts of terrestrial vertebrates (Graham et al., 2019), and vascular plants (Butt et al., 2013; Gallagher et al., 2019). We then performed a full MaxEnt model run which included the 35 variables described in the above section, for each species. We reviewed the importance of variables based on the average percent contribution and percent importance values across all species. The percent contribution is a measure of the contribution of

each variable towards model fit after each iteration of the MaxEnt model, while the percent importance is a measure of the importance of each variable towards model fit for the final MaxEnt model. We also categorised bioclimatic variables based on complexity and favoured simple variables as they tended to be less correlated with one another (Low et al., 2021).

This combined approach to variable selection resulted in 18 variables which moved through to the model fitting stage (Table 1): 7 bioclimatic variables and 11 soil and landscape variables. All bioclimatic variables selected for this study were included in CliMAS models (Graham et al., 2019) and similar modelling efforts for Australian plants (Gallagher et al., 2019), and all bioclimatic variables with the exception of BIO15 were considered to be simple climate variables (Low et al., 2021) (Table 1). All bioclimatic variables except for BIO05 had high or moderate importance values in the full model. Similarly, we included additional soil and landscape variables (Hageer et al., 2017) based on their use in recent biodiversity models (Gallagher et al., 2019), and we favored soil and landscape variables that were simpler.

[INSERT TABLE 1]

Model validation

Once variables were selected, models were re-run, and model performance was assessed based on the area under the curve (AUC) value, with AUC values of 0.7 or below indicating poor performance. This process resulted in 33 birds, 4 vascular plants, 1 mammal, 0 reptiles and 0 amphibians with AUC valus less than 0.7. The median AUC across all models was 0.9714. Prior to using species data, please ensure you check the AUC value which is contained withing the maxentResults.csv file.

[INSERT FIGURE 2]

Figure 2 *Distribution of AUC values for species models.*

Model projections

Using the best model selected in the model fitting procedure we projected species-level MaxEnt models under the future climate scenarios RCP2.6-SSP1, RCP4.5-SSP2, RCP7.0-SSP3 and RCP8.5-SSP5, 8 GCMs, for 1 historical time-period (1990) and 4 future time-periods (2030, 2050, 2070, 2090) using the lambda files produced in the model fitting step. Using the predicted habitat suitability data, we then calculated an ensemble average (mean), minimum and maximum habitat suitability (to capture model variance) across 8 GCMs for each species, climate scenario and time-period.

Geospatial calculations

To describe the patterns of habitat suitability across time in an accessible tabular format we calculated the total quality-weighted sum of habitat suitability for each species under different climate scenarios at each time period (Equation 1). We first adjusted the resolution of the rasters to $1km^2$, therefore the quality-weighted habitat area (*qwHA*) sum corresponds to the 'habitat area' in km^2. For example, if the probable habitat suitability in a cell is equal to 1, the cell is equivalent to $1km^2$, whereas if the probable habitat suitability in a cell is equal to 0.3, the cell is equivalent to $0.3km^2$. Noting that the quality-weighted habitat area is not equivalent to the realised area available for a species given ecological or land use constraints which can both influence habitat availability and suitability for species. The probability of habitat suitability (*p*) was summed across raster cells (*xy*), for each species (*j*), year (*y*) and climate scenario (*c*):

$$qwHA_{jyc} = \sum_{i=1}^{n} p_{jyc,xy}$$

(Equation 1)

To describe how the patterns of habitat suitability may have changed across space under different climate scenarios or years, we summarised raster data for each species in multiple ways. For each species (*t*) we calculated changes in habitat suitability (*s*) by subtracting future time periods and climate scenarios (*yc*) by historical climate niche (p^h). Where positive values indicate areas that increase in suitability in the

future and negative values indicate areas that decrease in climate suitability in the future. We provide visual representation of this information in Figure 6, and included the absolute and proportional change in habitat area in the tabular summaries provided for species:

$$s_t^{yc} = p_t^h - p_t^{yc}$$

(Equation 2)

To spatially identify important areas of climate refugia which was done for Figure 5, we multiplied the historical habitat suitability matrix by the habitat suitability in each future climate scenario and year combination. For each the cell, the probability of habitat suitability values per cell (*p*), for each species (*t*), year (*y*) and climate scenario (*c*) were multiplied by the future habitat suitability. Cell values were then divided by 100, and the resulting cell value represents climate refugia (*r*) between 0 to 100.

$$r_t^{yc} = (p_t^h * p_t^{yc})/100$$

(Equation 3)

Re-use potential

Code availability

For each species, MaxEnt models were run directly from the terminal using java and bash syntax and were ultimately executed using SLURM on a high-performance Linux-based computer cluster. Additional modelling and geospatial analyses were processed using a shell file executed using SLURM on the computer cluster. Data and geospatial analyses were conducted in R version 4.0.1 (R Core Team., 2020), key libraries include the ‘tidyverse’ (Wickham et al., 2019), ‘sf’ (Pebesma, 2018) and ‘raster’ (Hijmans, 2021). We used Python version 3.8.3 as well as the Geospatial Data Abstraction Library (GDAL). The scripts used in to generate this data is available in the GitHub repository, (see, https://github.com/CarlaBirdy/MaxEnt-habitat-models).

Dataset

Individual species' maps for historical and future minimum, mean and maximum ensembled habitat suitability, as well as the MaxEnt lambda file and summary reports produced in this study are publicly accessible for download on the open-access companion GigaDB database (which upholds the FAIR principles, Wilkinson et al., 2016). This dataset includes species-level historical (1970-2000 centered on 1990) and the future minimum, mean and maximum habitat suitability projections for 1,382 terrestrial vertebrates (182 amphibians, 487 birds, 178 mammals and 535 reptiles) and 9,251 vascular plants under 4 climate scenarios and 5 time periods, this data equates to 521,017 rasters that are compressed using Lempel–Ziv–Welch (lzw) compression. Additionally, for each species we have included a .csv file which contains the total quality-weighted habitat areas (in km^2) for each species under each different climate scenario and time period. We have also consolidated these tables across all species and included this tabular data. A complete list of the species for which habitat suitability maps were produced can be found in the companion GigaDB database.

Spatial resolution of data

This data is presented at $5km^2$ resolution which is aligned with the climate data used as key inputs to the MaxEnt model. The data can be subsequently downscaled to finer resolutions, however assumptions will have to be made about how habitat suitability is distributed across cells. The current resolution of this data is best utilised to understand general trends across space and time. To demonstrate the resolution, we present the southern cassowary (*Casuarius casuarius*) which is known to occur in the Wet Tropics region of Queensland, Australia. Current suitable areas for the southern cassowary are predicted to occur between Townsville to Cooktown, with an isolated area around the Iron Range (Figure 3). Taking the most severe climate change scenario (RCP8.5 - SSP5), the environmental space for the southern cassowary is predicted to reduce over time around its central habitat in the Atherton Tablelands. The maps for the southern cassowary can be compared with (Graham et al., 2019) for reference.

[INSERT FIGURE 3]

Figure 3 *This habitat suitability distribution is for the Southern cassowary (Casuarius casuarius) and presents its historical suitability projection. This zoomed in location map highlights the resolution of the data, and how the habitat suitability distribution for the Southern cassowary is modeled over time under the RCP8.5 - SSP5 scenario.*

Species-level data summary

The dataset includes suitability maps for species under different climate scenarios and time periods using an ensemble average approach. Through the process of ensemble averaging, the minimum and maximum suitability maps were also produced. These maps can be compared to understand the bounds of how climate change may generally impact habitat suitability in the future. The importance of incorporating multiple GCM projections can be seen by the variation among the minimum, mean, and maximum suitability maps (Figure 4). For the common wallaroo (*Macropus robustus*), the differences between the minimum, mean, and maximum suitability maps are most apparent under worsening climate scenarios. Areas across the southern parts of Australia remain suitable across all three suitability maps, compared to areas in the central and northern parts of their range becoming progressively less suitable. These trends are consistent with other macropod modelling studies that also suggest suitability for the common wallaroo will track south as climate scenarios worsen (Ritchie & Bolitho, 2008). The maps for the common wallaroo can also be compared with (Graham et al., 2019) for reference.

[INSERT FIGURE 4]

Figure 4 *Minimum, mean, and maximum suitability value across GCMs. These habitat suitability distributions are for the common wallaroo (Macropus robustus) for four future emission scenarios in the year 2090.*

Spatial changes over time

Taking this a step further, geospatial calculations can also be applied to determine the differences between years or climate scenarios. This can be conducted to identify areas of refugia (Equation 3), or the location and magnitude of change between different time periods (Equation 2). To calculate refugia, historical and future suitability maps can be multiplied together to accentuate areas in space that are suitable in both time periods. To calculate changes in habitat suitability, historical and future suitability maps can be

subtracted from one another to accentuate areas in space that have changed in suitability across time periods. Using the snow gum (*Eucalyptus pauciflora*) as an example, we find refugia in the alpine region of Australia is predicted to decline for the snow gum under worsening climate scenarios, with declines being most severe in the year 2090 (Figure 5, top). Across all climate scenarios habitat suitability is declining from all areas of the snow gum's range, and we did not identify areas of increases (Figure 5, bottom).

[INSERT FIGURE 5]

***Figure 5** These refugia and habitat suitability change maps are for the snow gum (Eucalyptus pauciflora). The Top Panel present climate change refugia for four future emission scenarios in the years 2030 and 2050. Darker blue on the refugia maps represent areas that have high predictive suitability historically as well in future time periods. The Bottom Panel present changes in habitat suitability for four future emission scenarios in the years 2030 and 2050. Darker orange areas indicate places that decrease in suitability compared to the previous time period, and white areas indicate no change in suitability.*

Changes in quality-weighted habitat area

The dataset also includes a tabular summary of quality-weighted habitat area in km^2 for each species under different climate scenarios and time periods (Equation 1 and Equation 2). The quality-weighted habitat area values can be analysed and plotted to understand the how climate change may impact habitat area for single species or groups of species in the future (Figure 6). When this data is summarised across all species, we can show that in 2030 the distribution of change in habitat area are similar across the four climate scenarios. However, in 2090 the distribution of change in habitat area follows a different pattern across climate scenarios with progressively more species loosing progressively more habitat area as climate scenario worsens (Figure 6).

[INSERT FIGURE 6]

***Figure 6** Histogram of the number of species and their relative change in quality weighted habitat area between 1990 and each future time period (2030, 2050, 2070, 2090).*

Discussion

Spatial data on the suitability of areas for species is an important input to guide conservation planning, policy and management. The objective of this paper was to develop habitat suitability maps for Australian flora and fauna under different climate futures using a MaxEnt approach. This data has been developed in a way that is consistent across species and enables users to analyse how different climate futures may impact the habitat suitability for biodiversity more generally across Australia. This data can also be used for species-level analysis and can be a starting point for additional analyses which utilise either geospatial information or tabular information that could take into consideration additional information like land use, conservation actions or species ecology.

This spatial and tabular dataset is ideal for users that would like to understand how the habitat suitability of areas for species is predicted to change over time or under different climate scenarios. Due to its $5km^2$ spatial resolution, the data is best for understanding broader spatial trends that can be integrated into spatial planning (Maxwell et al., 2019), rather than more local management such as identifying specific sites for translocation (Eyre et al., 2022). For example, as presented above, these maps can be combined to evaluate how habitat suitability changes over time (Figure 6) or over space and time (Figure 5), which can then be considered into conservation or monitoring plans in areas which are predicted to lose or gain suitable areas for the species. These analyses can be conducted at a species or a taxonomic group level to support conservation actions for species of interest (e.g., threatened species) or for biodiversity values more generally. Spatial information about species could be directly utilised to develop management or monitoring plans that consider how climate change may impact the species habitat area.

When using and interpreting the data contained in this data set it is important to consider the following limitations and considerations. This dataset presents the habitat suitability of areas for species under different climate scenarios and time periods. These maps are not distribution maps, rather they present

habitat suitability based on climate, soil and landscape characteristics. These maps have not been thresholded nor do they consider dispersal (Graham et al., 2019), land use (Kapitza et al., 2021), biophysical capacity (Briscoe et al., 2023), or attributes that may be important for species of interest (e.g., fire or vegetation structure e.g., Eyre et al., 2022). The occurrence points used for this analysis were those originally used for the CliMAS work, and the ALA data were passed through an additional rigorous cleaning process. This process helped reduce the spatial bias and noise in the occurrence points (Phillips et al., 2009); however, more broadly there are sampling biases that influence the distribution of occurrence points, such as land tenure. To improve on the models, an integrated pathway to ALA into the modelling procedure would be ideal as this would ensure up-to-date input data. However, this can also come with challenges as occurrence data is required to have the same temporal resolution to the historical or current climate data (i.e., 1990 in this study). MaxEnt models are prone to overfit but are also less influenced by collinearity than statistical models, we tried mitigating the impacts of overfitting the MaxEnt models by conducting variable selection. There are a multitude of other methods to model suitability and species distributions that have their own use cases and limitations (Elith & Graham, 2009).

To spatially target conservation actions, spatial information about the location and suitability of areas for species is needed. This study provides a comprehensive data set of predicted habitat suitability under 4 climate futures, while also incorporating the uncertainty across GCMs. We are providing a spatial and tabular data product at the Australian scale and at $5km^2$ resolution that can be used to inform research and decision making at local, regional and national scales. This data can be applied within strategic conservation planning approaches and can be used to identify important areas for species consecration (Tulloch et al., 2015). Spatial information about current and future suitable areas for species is a key component of conservation planning, particularly as the impact of climate change on species and biodiversity is uncertain.

Data availability

All spatial and tabular data are freely accessible in the companion GigaDB repository.

Acknowledgements

This work was made possible by generous philanthropic support for Climateworks Centre's Land Use Futures program which supported BB and CA. CA was also supported by an Alfred Deakin Postdoctoral Research Fellowship 2023-2025. DS was partly funded by the 2021-2024 ARC linkage grant Innovation in agricultural sector Greenhouse Gas abatement in New South Wales, led by Prof Jeff Connor. This work would also like to acknowledge those originally involved with the CliMAS project.

Conflict of interest

The authors declare no conflicts of interest.

References

Australian Government Department of Agriculture and the Environment. (2021). *Species Profile and Threats Database (SPRAT)*. http://www.environment.gov.au/cgi-bin/sprat/public/sprat.pl

Bergstrom, D. M., Wienecke, B. C., van den Hoff, J., Hughes, L., Lindenmayer, D. B., Ainsworth, T. D., Baker, C. M., Bland, L., Bowman, D. M. J. S., Brooks, S. T., Canadell, J. G., Constable, A. J., Dafforn, K. A., Depledge, M. H., Dickson, C. R., Duke, N. C., Helmstedt, K. J., Holz, A., Johnson, C. R., … Shaw, J. D. (2021). Combating ecosystem collapse from the tropics to the Antarctic. *Global Change Biology*, *27*(9), 1692–1703. https://doi.org/10.1111/gcb.15539

Briscoe, N. J., Morris, S. D., Mathewson, P. D., Buckley, L. B., Jusup, M., Levy, O., Maclean, I. M. D., Pincebourde, S., Riddell, E. A., Roberts, J. A., Schouten, R., Sears, M. W., & Kearney, M. R. (2023). Mechanistic forecasts of species responses to climate change: The promise of biophysical ecology. In *Global Change Biology* (Vol. 29, Issue 6, pp. 1451–1470). John Wiley and Sons Inc. https://doi.org/10.1111/gcb.16557

Bryan, B. A., Nolan, M., Harwood, T. D., Connor, J. D., Navarro-Garcia, J., King, D., Summers, D. M., Newth, D., Cai, Y., Grigg, N., Harman, I., Crossman, N. D., Grundy, M. J., Finnigan, J. J., Ferrier, S., Williams, K. J., Wilson, K. A., Law, E. A., & Hatfield-Dodds, S. (2014). Supply of carbon sequestration and biodiversity services from Australia's agricultural land under global change. *Global Environmental Change*, *28*, 166–181. https://doi.org/10.1016/j.gloenvcha.2014.06.013

Butt, N., Pollock, L. J., & Mcalpine, C. A. (2013). Eucalypts face increasing climate stress. *Ecology and Evolution*, *3*(15), 5011–5022. https://doi.org/10.1002/ece3.873

Chapman, A. D. (2009). *Numbers of Living Species in Australia and the World*. Biodiversity Information Services.

Coleman, S. (2016). Australia state of the environment 2016: built environment. In *independent report to the Australian Government Minister for the Environment and Energy*. https://doi.org/10.30547/mediascope.1.2018.6

Elith, J., & Graham, C. H. (2009). Do they? How do they? WHY do they differ? on finding reasons for differing performances of species distribution models. *Ecography*, *32*(1), 66–77. https://doi.org/10.1111/j.1600-0587.2008.05505.x

Eyre, A. C., Briscoe, N. J., Harley, D. K. P., Lumsden, L. F., McComb, L. B., & Lentini, P. E. (2022). Using species distribution models and decision tools to direct surveys and identify potential translocation sites for a critically endangered species. *Diversity and Distributions*, *28*(4), 700–711. https://doi.org/10.1111/ddi.13469

Feng, X., Park, D. S., Liang, Y., Pandey, R., & Papeş, M. (2019). Collinearity in ecological niche modeling: Confusions and challenges. *Ecology and Evolution*, *9*(18), 10365–10376. https://doi.org/10.1002/ece3.5555

Gallagher, R. V., Allen, S., & Wright, I. J. (2019). Safety margins and adaptive capacity of vegetation to climate change. *Scientific Reports*, *9*(1), 1–11. https://doi.org/10.1038/s41598-019-44483-x

Garnett, S., Hayward-Brown, B. K., Kopf, R. K., Woinarski, J. C. Z., Cameron, K. A., Chapple, D. G., Copley, P., Fisher, A., Gillespie, G., Latch, P., Legge, S., Lintermans, M., Moorrees, A., Page, M.,

Renwick, J., Birrell, J., Kelly, D., & Geyle, H. M. (2022). Australia's most imperilled vertebrates. *Biological Conservation*, *270*, 109561. https://doi.org/10.1016/j.biocon.2022.109561

Graham, E. M., Reside, A. E., Atkinson, I., Baird, D., Hodgson, L., James, C. S., & VanDerWal, J. J. (2019). Climate change and biodiversity in Australia: a systematic modelling approach to nationwide species distributions. *Australasian Journal of Environmental Management*, *26*(2), 112–123. https://doi.org/10.1080/14486563.2019.1599742

Hageer, Y., Esperón-Rodríguez, M., Baumgartner, J. B., & Beaumont, L. J. (2017). Climate, soil or both? Which variables are better predictors of the distributions of Australian shrub species? *PeerJ*, *2017*(6). https://doi.org/10.7717/peerj.3446

Hanson, J. O., Schuster, R., Strimas-Mackey, M., & Bennett, J. R. (2019). Optimality in prioritizing conservation projects. *Methods in Ecology and Evolution*, *10*(10), 1655–1663. https://doi.org/10.1111/2041-210X.13264

Hijmans, R. J. (2021). *raster: Geographic Data Analysis and Modeling* (R package version 3.5-9). https://CRAN.R-project.org/package=raster.

Kapitza, S., Van Ha, P., Kompas, T., Golding, N., Cadenhead, N. C. R., Bal, P., & Wintle, B. A. (2021). Assessing biophysical and socio-economic impacts of climate change on regional avian biodiversity. *Scientific Reports*, *11*(1). https://doi.org/10.1038/s41598-021-82474-z

Kearney, S. G., Cawardine, J., Reside, A. E., Fisher, D. O., Maron, M., Doherty, T. S., Legge, S., Silcock, J., Woinarski, J. C. Z., Garnett, S. T., Wintle, B. A., & Watson, J. E. M. (2018). The threats to Australia's imperilled species and implications for a national conservation response. *Pacific Conservation Biology*, *Chapman 2009*. https://doi.org/10.1071/PC18024

Leclère, D., Obersteiner, M., Barrett, M., Butchart, S. H. M., Chaudhary, A., De Palma, A., DeClerck, F. A. J., Di Marco, M., Doelman, J. C., Dürauer, M., Freeman, R., Harfoot, M., Hasegawa, T., Hellweg, S., Hilbers, J. P., Hill, S. L. L., Humpenöder, F., Jennings, N., Krisztin, T., … Young, L. (2020). Bending the curve of terrestrial biodiversity needs an integrated strategy. *Nature*, *585*(October 2018). https://doi.org/10.1038/s41586-020-2705-y

Low, B. W., Zeng, Y., Tan, H. H., & Yeo, D. C. J. (2021). Predictor complexity and feature selection affect Maxent model transferability: Evidence from global freshwater invasive species. *Diversity and Distributions*, *27*(3), 497–511. https://doi.org/10.1111/ddi.13211

Maxwell, S. L., Reside, A., Trezise, J., McAlpine, C. A., & Watson, J. E. (2019). Retention and restoration priorities for climate adaptation in a multi-use landscape. *Global Ecology and Conservation*, *18*, e00649. https://doi.org/10.1016/j.gecco.2019.e00649

Meehl, G. A., Covey, C., Delworth, T., Latif, M., Mcavaney, B., Mitchell, J. F. B., Stouffer, R. J., & Taylor, K. E. (2007). *The WCRP CMIP3 Multi-model Dataset: A New Era in Climate Change Research*. http://cera-www.dkrz.de/IPCC_DDC/

Pebesma, E. (2018). Simple Features for R: Standardized Support for Spatial Vector Data. *The R Journal*, *10*(1), 439–446.

Phillips, S. B., Aneja, V. P., Kang, D., & Arya, S. P. (2006). Maximum entropy modeling of species geographic distributions. *Ecological Modelling*, *6*(2–3), 231–252. https://doi.org/10.1016/j.ecolmodel.2005.03.026

Phillips, S. J., Dudík, M., Elith, J., Graham, C. H., Lehmann, A., Leathwick, J., & Ferrier, S. (2009). Sample selection bias and presence-only distribution models: Implications for background and pseudo-absence data. *Ecological Applications*, *19*(1), 181–197. https://doi.org/10.1890/07-2153.1

R Core Team. (2020). *R: A language and environment for statistical computing* (4.0.1). R Foundation for Statistical Computing.

Reside, A. E., Welbergen, J. A., Phillips, B. L., Wardell-Johnson, G. W., Keppel, G., Ferrier, S., Williams, S. E., & Vanderwal, J. (2014). Characteristics of climate change refugia for Australian biodiversity. *Austral Ecology*, *39*(8), 887–897. https://doi.org/10.1111/aec.12146

Ritchie, E. G., & Bolitho, E. E. (2008). Australia's savanna herbivores: Bioclimatic distributions and an assessment of the potential impact of regional climate change. *Physiological and Biochemical Zoology*, *81*(6), 880–890. https://doi.org/10.1086/588171

Summers, D. M., Bryan, B. A., Crossman, N. D., & Meyer, W. S. (2012). Species vulnerability to climate change: Impacts on spatial conservation priorities and species representation. *Global Change Biology*, *18*(7), 2335–2348. https://doi.org/10.1111/j.1365-2486.2012.02700.x

Tulloch, V. J. D., Tulloch, A. I. T., Visconti, P., Halpern, B. S., Watson, J. E. M., Evans, M. C., Auerbach, N. A., Barnes, M., Beger, M., Chadès, I., Giakoumi, S., McDonald-Madden, E., Murray, N. J., Ringma, J., & Possingham, H. P. (2015). Why do We map threats? Linking threat mapping with actions to make better conservation decisions. *Frontiers in Ecology and the Environment*, *13*(2), 91–99. https://doi.org/10.1890/140022

Ward, M., Tulloch, A., Stewart, R., Possingham, H. P., Legge, S., Gallagher, R. V., Graham, E. M., Southwell, D., Keith, D., Dixon, K., Yong, C., Carwardine, J., Cronin, T., Reside, A. E., & Watson, J. E. M. (2022). Restoring habitat for fire-impacted species' across degraded Australian landscapes. *Environmental Research Letters*, *17*(8). https://doi.org/10.1088/1748-9326/ac83da

Wickham, H., Averick, M., Bryan, J., Chang, W., McGowan, L. D., François, R., Grolemund, G., Hayes, A., Henry, L., Hester, J., Kuhn, M., Lin Pedersen, T., Miller, E., Milton Bache, S., Müller, K., Ooms, J., Robinson, D., Paige Seidel, D., Spinu, V., … Yutani, H. (2019). Welcome to the tidyverse. *Journal of Open Source Software*, *4*(43), 1686.

Wilkinson, M. D., Dumontier, M., Aalbersberg, Ij. J., Appleton, G., Axton, M., Baak, A., Blomberg, N., Boiten, J. W., da Silva Santos, L. B., Bourne, P. E., Bouwman, J., Brookes, A. J., Clark, T., Crosas, M., Dillo, I., Dumon, O., Edmunds, S., Evelo, C. T., Finkers, R., … Mons, B. (2016). Comment: The FAIR Guiding Principles for scientific data management and stewardship. *Scientific Data*, *3*. https://doi.org/10.1038/sdata.2016.18

Woinarski, J., Braby, M. F., Burbidge, A. A., Coates, D., Garnett, S. T., Fensham, R. J., Legge, S. M., McKenzie, N. L., Silcock, J. L., & Murphy, B. P. (2019). Reading the black book: The number, timing, distribution and causes of listed extinctions in Australia. *Biological Conservation*, *239*(November), 108261. https://doi.org/10.1016/j.biocon.2019.108261

 Click here to access/download;Table;table_1.docx

Table 1 *Summary of the bioclimatic, soil and landscape variable selected in the final MaxEnt model.*

Code	Variable Name	Contribution[1]	Importance[2]	Ecological Rationale
Bioclimatic variables				
BIO1	Annual Mean Temperature	8.72	18.21	Influences thermal tolerances of species.
BIO5	Max Temperature of Warmest Month	6.33	9.92	Influences upper thermal tolerances of species through extreme temperatures.
BIO6	Min Temperature of Coldest Month	4.30	8.66	Influences lower thermal tolerances of species through extreme temperatures.
BIO12	Annual Precipitation	8.60	10.81	Average annual rainfall which influences water availability.
BIO13	Precipitation of Wettest Month	17.67	7.77	Maximum rainfall in the wettest month which influences maximum water availability.
BIO14	Precipitation of Driest Month	14.93	8.45	Minimum rainfall in the driest month which influences minimum water availability.
BIO15	Precipitation Seasonality	12.13	13.20	Standard deviation of rainfall in the annually which influences the variation in water availability.
Soil and landscape variables				
AWC	Available Water Capacity	0.94	0.68	The amount of water held by the soil for future use.
BDW	Bulk Density (Whole Earth)	0.89	1.17	Soil's ability to function for structural support, water and nutrient and microbial life movement, and soil aeration.
CLY	Clay	1.04	0.95	Promotes water retention and reduces air circulation in soil.
DES	Depth of Soil	2.00	1.29	Defines the root space and volume of soils available.
ECE	Electroconductivity	3.39	5.21	Movement of nutrients within the soil which influences the availability of soil nutrients.
elev	Elevation	2.37	1.57	Elevation influences soil properties and air pressure.
pHc	pH	5.43	4.30	Affects the amount of nutrients that are water soluble in soil.
slope	Slope Relief	1.81	1.00	Influences soil properties and creates varying microclimates.
SLT	Silt	2.63	2.10	Promotes water retention and creates relatively porous soil conditions.
SND	Sand	1.60	1.60	Promotes water drainage and air circulation in soil.
SOC	Organic Carbon	5.17	3.05	Promotes soil structure by providing a food source for micro-organisms.

[1] *Average (mean) percent contribution in the final models for each environmental variable across all species. A measure of the contribution of each variable towards model fit after each iteration of the MaxEnt model.*

[2] *Average (mean) percent importance in the final models for each environmental variable across all species. A measure of the importance of each variable measure depends the resulting decrease in training AUC on the final MaxEnt model.*

Figure 1

Click here to access/download;Figure;figure_1.png

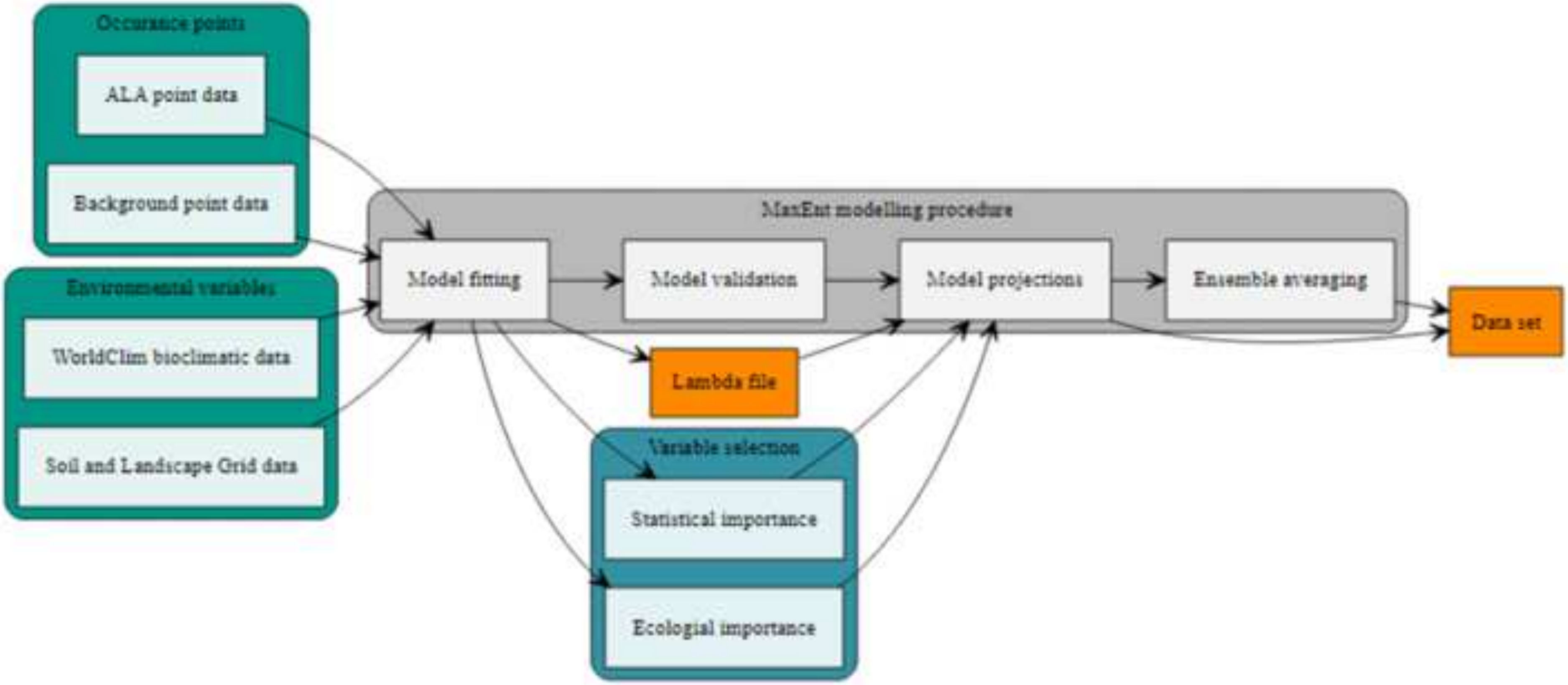

Figure 2

Click here to access/download;Figure;figure_2.png

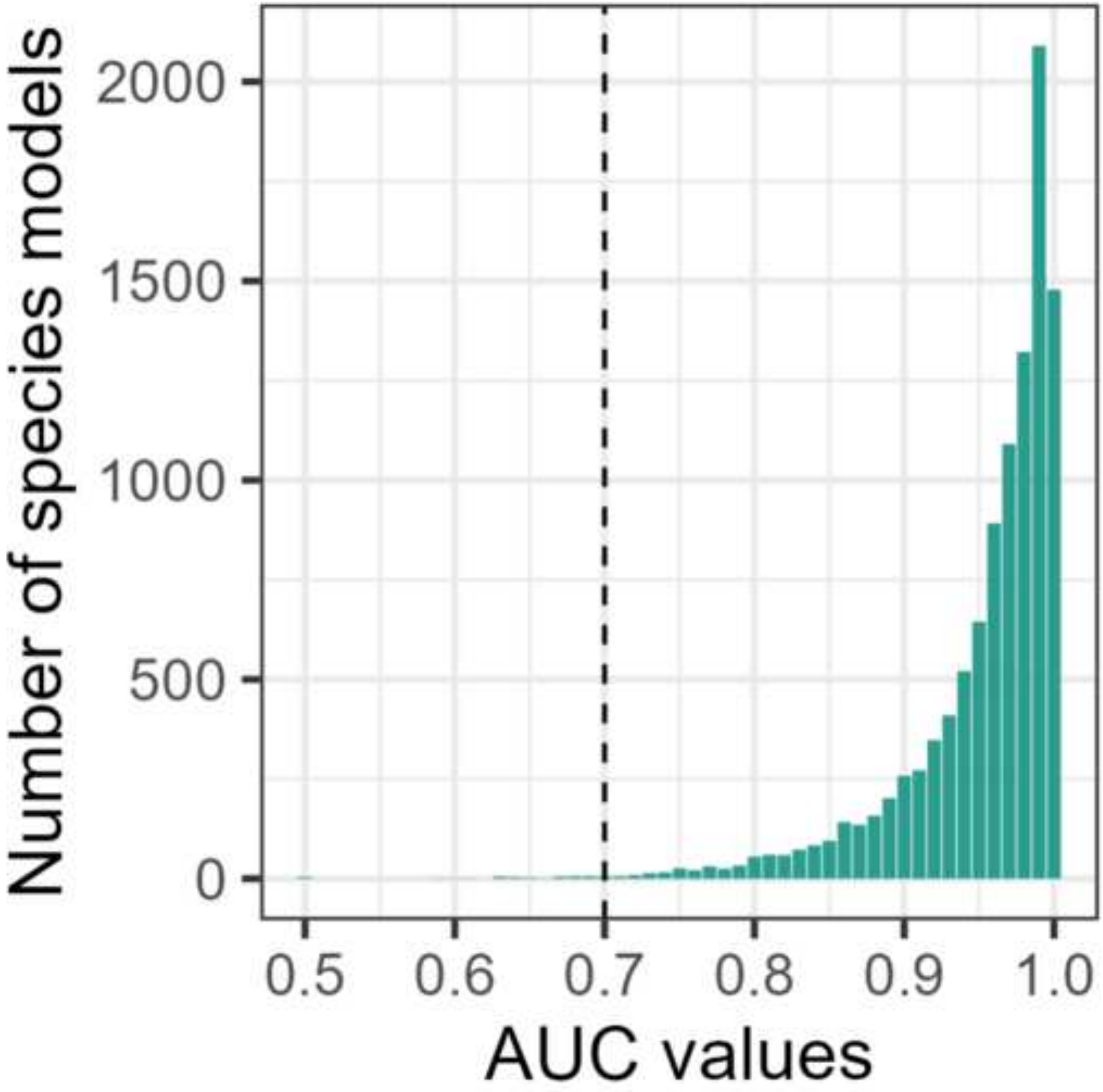

Figure 3

Click here to access/download;Figure;figure_3.png

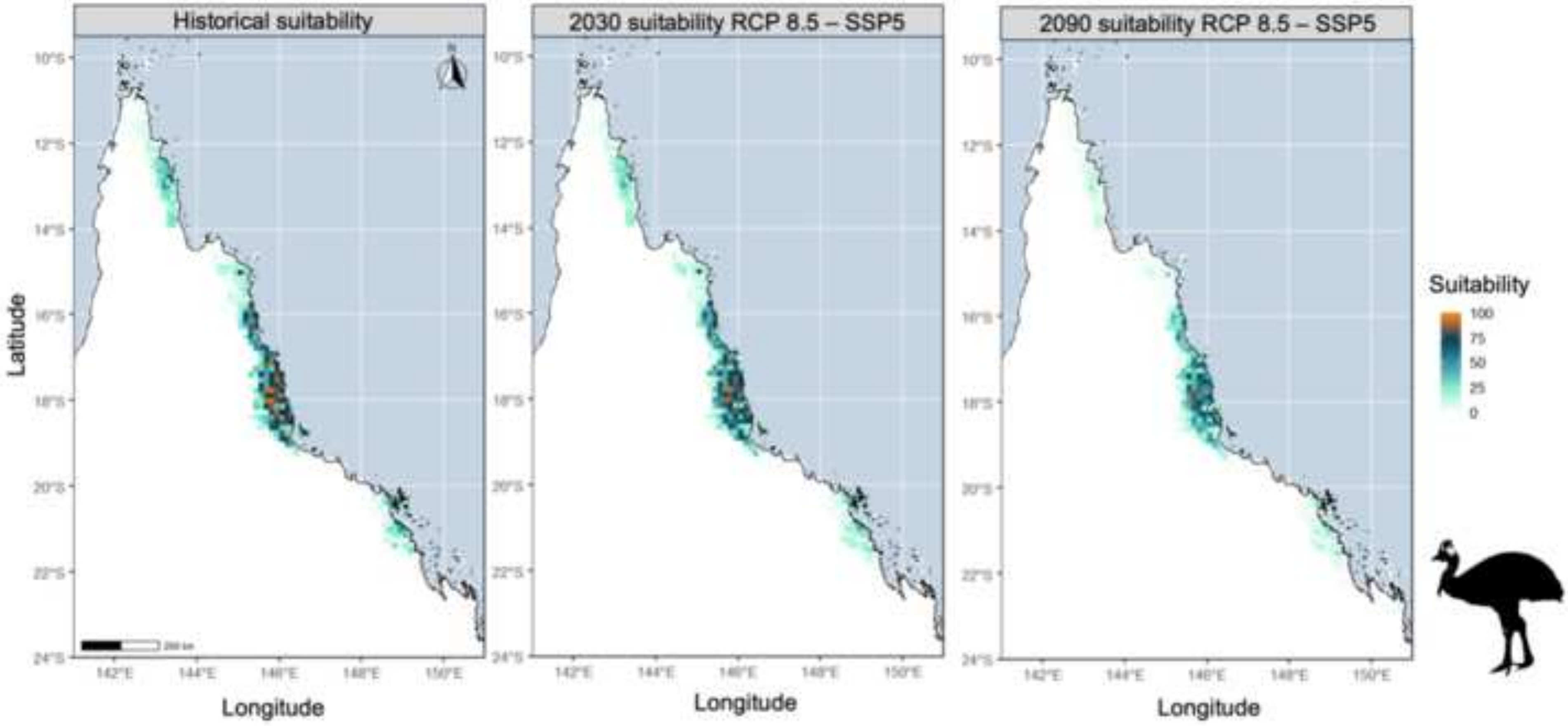

Figure 4

Click here to access/download;Figure;figure_4.png

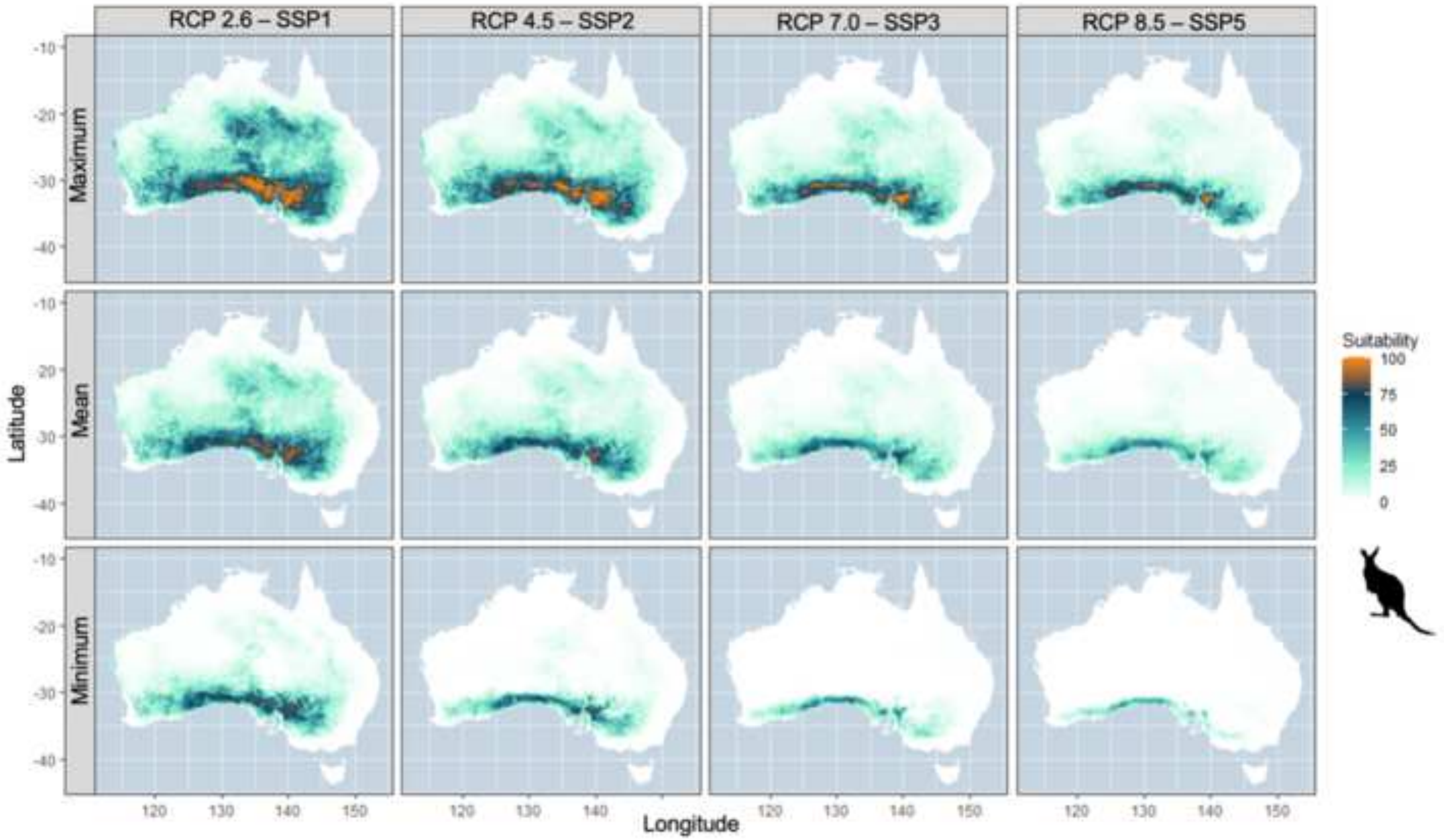

Figure 5 Click here to access/download;Figure;figure_5.png

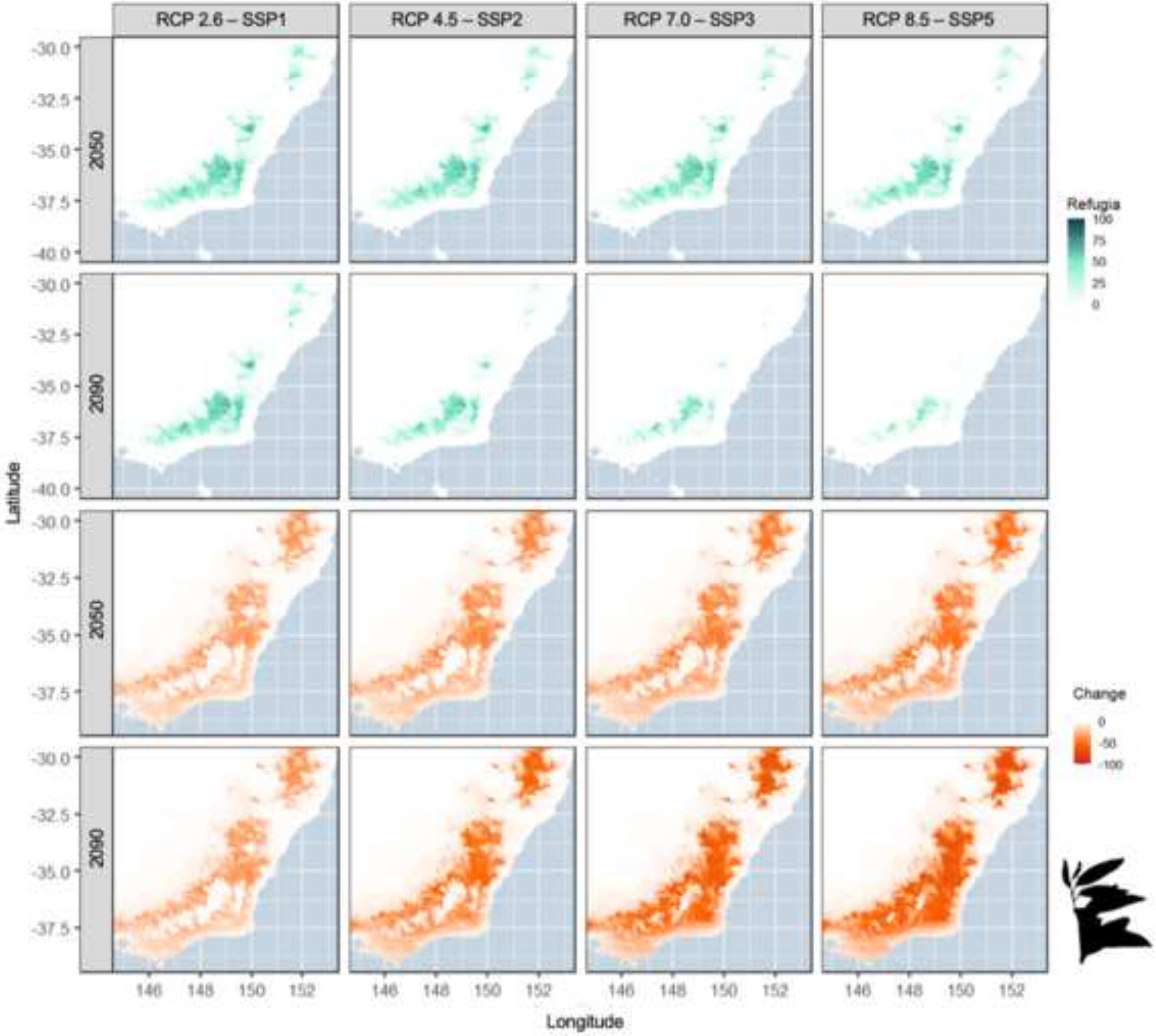

Figure 6

Click here to access/download;Figure;figure_6.png

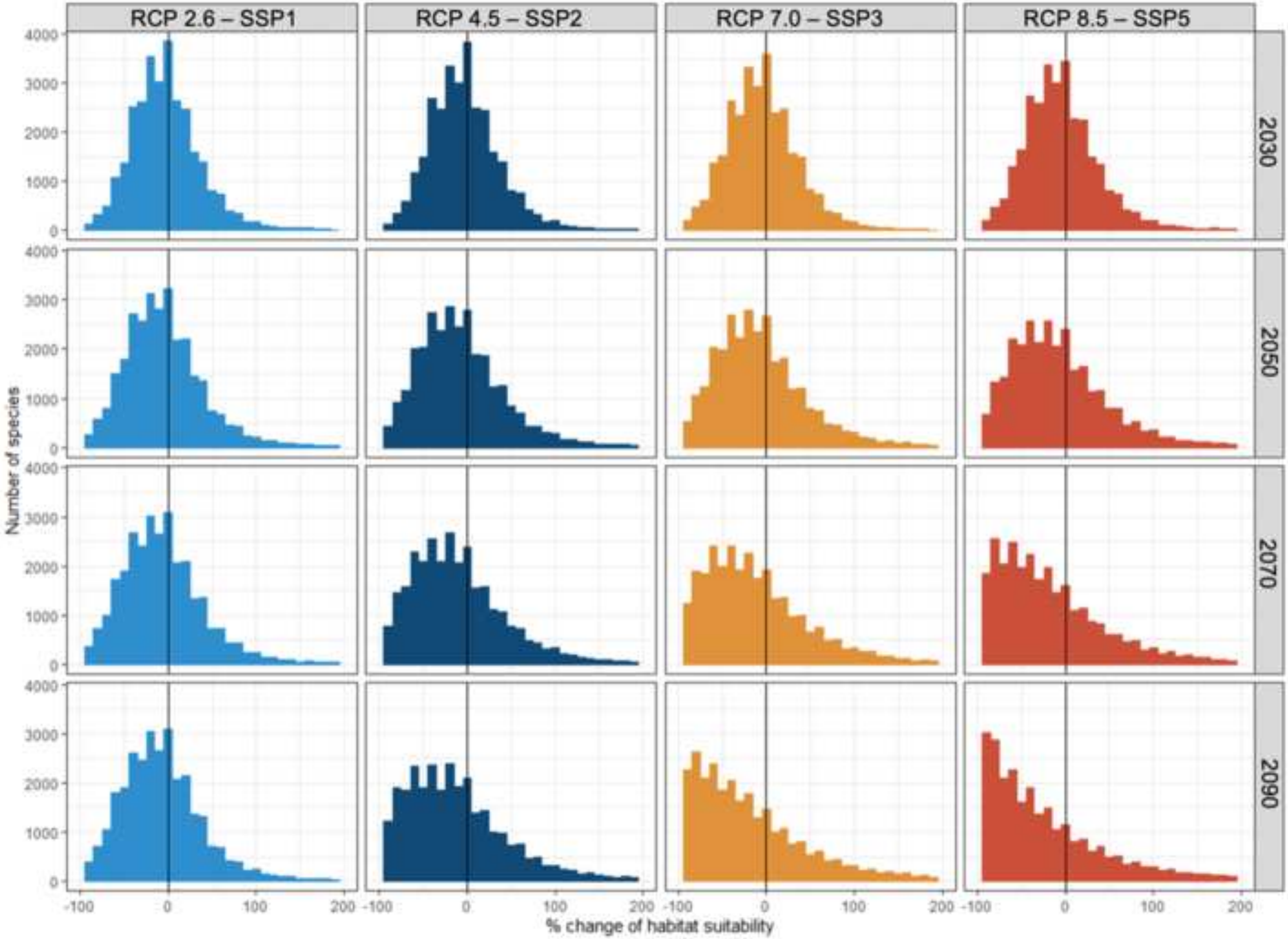

Graphical abstract

Click here to access/download;Figure;graphical_abstract.png

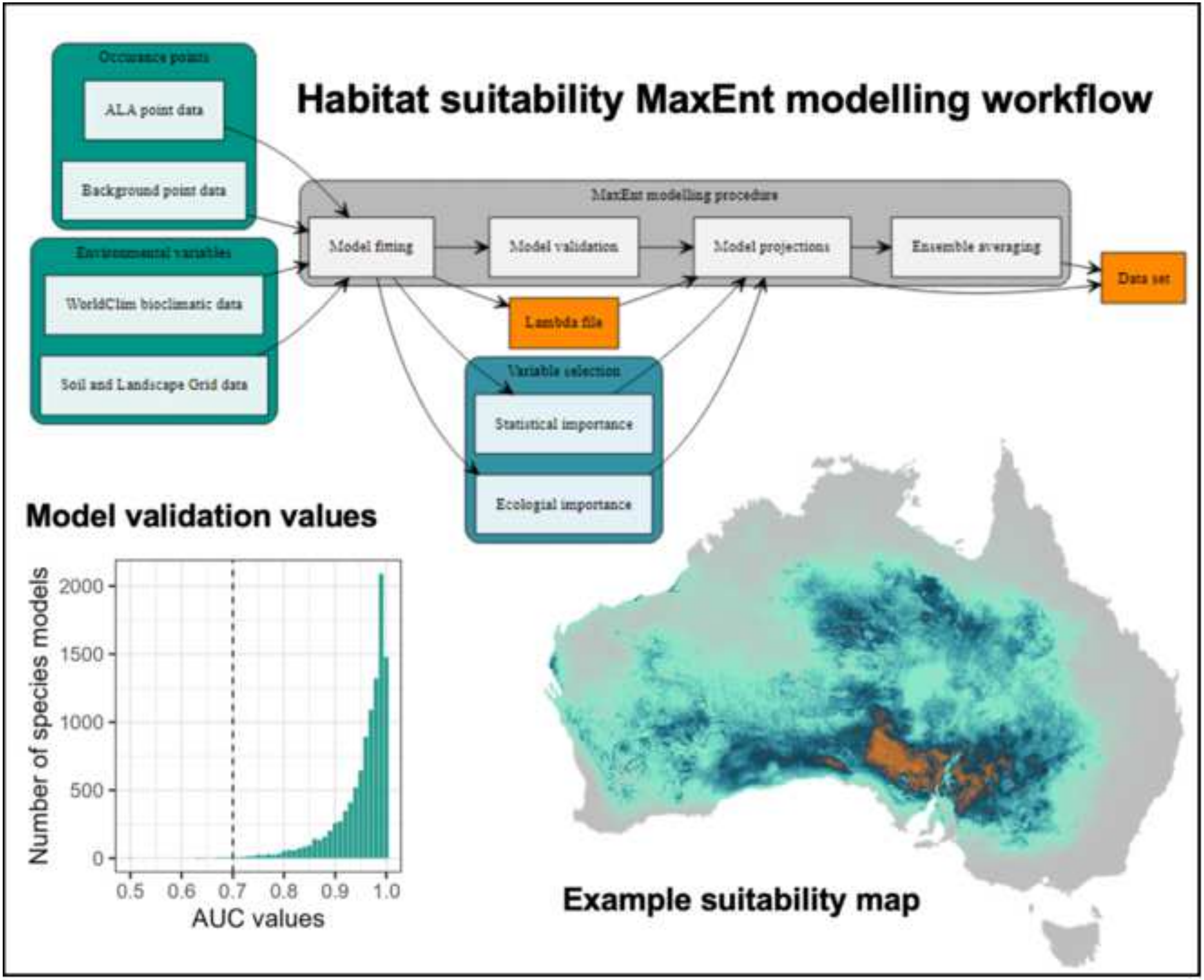

Click here to access/download
Supplementary Material
sup-material_GigaDBUploadForm.xlsx

Personal Cover

Click here to access/download;Personal Cover;Cover_Letter.docx

Deakin University
School of Life and Environmental Sciences

DEAKIN UNIVERSITY

Dear Dr Laurie Goodman and Dr Scott Edmunds July 2023
Publishing Director | Editor-in-Chief
GigaScience

I wish to submit this Data Note titled '**Habitat suitability maps for Australian flora and fauna under CMIP6 climate scenarios**' which compliments the dataset 'Supporting data for "Habitat suitability maps for Australian flora and fauna under CMIP6 climate scenarios"' (to be hosted on *GigaDB)*, on behalf of my co-authors for consideration for publication in the journal *GigaScience.*

Australia is a global biodiversity hotspot, home to an extraordinary array of unique species and diverse, iconic ecosystems. Geospatial information on where species are now and where suitable areas may be in the future is the foundation of efficient planning for conservation action, particularly in areas where local conditions are more sensitive to climate change. However, developing continental-scale data for numerous species poses challenges due to the computational resources requirements as well as requires the handling and storing large datasets. The aim of this data compilation effort was to quantify the habitat suitability of Australian flora and fauna species under climate change and over time and provide the data on an open-access platform, such as *GigaScience*.

This Data Note outlines the significance, methodology and reuse potential for this exceptional dataset in the field of conservation science in a manner to encourage reuse:

- **Dataset size:** The data set we present to is approximately 60GB in size and is comprised mainly of compressed raster images, as well as tabular and text file data. This dataset includes species-level historical and future, minimum, mean and maximum habitat suitability projections for 1,382 terrestrial vertebrates and 9,251 vascular plants under 4 climate scenarios and 5 time periods, this data equates to 521,017 rasters.
- **FAIR (Findable, Accessible, Interoperable and Reusable) principles**: This project strives to be 'findable' and 'accessible' by being published in an open sources database such as *GigaScience* and *GigaDB*. We also stive for the data to be 'interoperable' through the publication of this data note as well as by providing a GitHub repository for the affiliated scripts. Finally, we aim that this data is 'reusable' as the resuse potential has been described in the paper, and we are hosting the data on GigaDB.
- **Novelty of data**: Currently there is no spatial data product available open access that provides information about habitat suitability for Australian species under climate change. In 2017, Graham et al. (2019) published a comprehensive spatial dataset of species distributions known as the CliMAS, however, the data portal was retired in 2020 due to updates in IPCC projections (CMIP6). This has left a gap in the biodiversity conservation data landscape in Australia.
- **Reuse potential:** The data are well documented in the Data Note, and we have included extensive metadata in the files sheet in the GigaDB repository. We provide unique information allowing novel uses of data which include spatial and temporal analysis of species as well as Australian. Additionally, the original CliMAS dataset, available through the web portal CliMAS, yielded 7,559 unique pageviews over a four-year period and many practical outcomes published in academic journals. Therefore, we are confident the data we are presented to *GigaScience* will have similar academic and practical application.

We confirm that this paper has not in whole or in part been published previously and is not currently under consideration for publication elsewhere. We look forward to your response.

Dr Carla Archibald, on behalf of all co-authors.
School of Life and Environmental Sciences
Deakin University
c.archibald@deakin.edu.au

Wilkinson, M. D., et al. (2016). The FAIR Guiding Principles for scientific data management and stewardship. Scientific Data, 3

Graham, E. M., et al. (2019). Climate change and biodiversity in Australia: a systematic modelling approach to nationwide species distributions. *Australasian Journal of Environmental Management*, *26*(2), 112–123
