# Supplementary material for: Habitat suitability maps for Australian flora and fauna under CMIP6 climate scenarios
Source: Gigascience. 2024 Mar 5;13:giae002. doi: 10.1093/gigascience/giae002 (PMC10939329; doi:10.1093/gigascience/giae002)
Supplement: giae002_GIGA-D-23-00183_Revision_1 [file giae002_giga-d-23-00183_revision_1.pdf]

GigaScience

Habitat suitability maps for Australian flora and fauna under CMIP6 climate scenarios

--Manuscript Draft--

**Manuscript Number:**	GIGA-D-23-00183R1
**Full Title:**	Habitat suitability maps for Australian flora and fauna under CMIP6 climate scenarios
**Article Type:**	Data Note
**Funding Information:**	
**Abstract:**	Background: Spatial information about the location and suitability of areas for native plant and animal species under different climate futures is an important input to land use and conservation planning and management. Australia, renowned for its abundant species diversity and endemism, often relies on modelled data to assess species distributions due to the country's vast size and the challenges associated with conducting on-ground surveys on such a large scale. The objective of this paper is to develop habitat suitability maps for Australian flora and fauna under different climate futures. Results: Using MaxEnt, we produced Australia-wide habitat suitability maps under RCP2.6-SSP1, RCP4.5-SSP2, RCP7.0-SSP3 and RCP8.5-SSP5 climate futures for 1,382 terrestrial vertebrates and 9,251 vascular plants vascular plants at 5km2 for open access. This represents 60% of all Australian mammal species, 77% of amphibian species, 50% of reptile species, 71% of bird species and 44% of vascular plant species. We also include tabular data which includes summaries of total quality-weighted habitat area of species under different climate scenarios and time periods. Conclusions: The spatial data supplied can help identify important and sensitive locations for species under various climate futures. Additionally, the supplied tabular data can provide insights into the impacts of climate change on biodiversity in Australia. These habitat suitability maps can be used as input data for landscape and conservation planning or species management, particularly under different climate change scenarios in Australia.
**Corresponding Author:**	Carla Leigh Archibald, Ph.D<br>Deakin University<br>Burwood, VIC AUSTRALIA
**Corresponding Author Secondary Information:**	
**Corresponding Author's Institution:**	Deakin University
**Corresponding Author's Secondary Institution:**	
**First Author:**	Carla L Archibald, Ph.D
**First Author Secondary Information:**	
**Order of Authors:**	Carla L Archibald, Ph.D
	David M Summers, Ph.D
	Erin M Graham, Ph.D
	Brett A Bryan, Ph.D
**Order of Authors Secondary Information:**	
**Response to Reviewers:**	Hello, we have provided our Reviewer Report as a word document, please refer to this document as it contains updated figures.<br>____<br><br>Associate Editor<br><br>Dear Carla,<br><br>Your manuscript "Habitat suitability maps for Australian flora and fauna under CMIP6 climate scenarios" (GIGA-D-23-00183) has been assessed by our reviewers. Although

Powered by Editorial Manager® and ProduXion Manager® from Aries Systems Corporation

it is of interest, we are unable to consider it for publication in its current form. The reviewers have raised a number of points which we believe would improve the manuscript and may allow a revised version to be published in GigaScience.

Their reports, together with any other comments, are below. Please also take a moment to check our website at https://aus01.safelinks.protection.outlook.com/?url=https%3A%2F%2Fwww.editorialmanager.com%2Fgiga%2F&data=05%7C01%7Cc.archibald%40deakin.edu.au%7Cb83f42e4d5064d41996308dbb018b752%7Cd02378ec168846d585401c28b5f470f6%7C0%7C0%7C638297393738149435%7CUnknown%7CTWFpbGZsb3d8eyJWIjoiMC4wLjAwMDAiLCJQIjoiV2luMzIiLCJBTiI6Ik1haWwiLCJXVCI6Mn0%3D%7C3000%7C%7C%7C&sdata=OVNQPHhHe9gvRwMTeo3zMAZJrUvc5C2G5yKxN9YUZlA%3D&reserved=0 for any additional comments that were saved as attachments.

In addition, please register any new software application in the bio.tools and SciCrunch.org databases to receive RRID (Research Resource Identification Initiative ID) and biotoolsID identifiers, and include these in your manuscript. Computational workflows should be registered in workflowhub.eu and the DOIs cited in the relevant places in the manuscript. These will facilitate tracking, reproducibility and re-use of your tool.

If you are able to fully address these points, we would encourage you to submit a revised manuscript to GigaScience. Once you have made the necessary corrections, please submit online at:

https://aus01.safelinks.protection.outlook.com/?url=https%3A%2F%2Fwww.editorialmanager.com%2Fgiga%2F&data=05%7C01%7Cc.archibald%40deakin.edu.au%7Cb83f42e4d5064d41996308dbb018b752%7Cd02378ec168846d585401c28b5f470f6%7C0%7C0%7C638297393738149435%7CUnknown%7CTWFpbGZsb3d8eyJWIjoiMC4wLjAwMDAiLCJQIjoiV2luMzIiLCJBTiI6Ik1haWwiLCJXVCI6Mn0%3D%7C3000%7C%7C%7C&sdata=OVNQPHhHe9gvRwMTeo3zMAZJrUvc5C2G5yKxN9YUZlA%3D&reserved=0

If you have forgotten your username or password please use the "Send Login Details" link to get your login information. For security reasons, your password will be reset.

Please include a point-by-point within the 'Response to Reviewers' box in the submission system. Please ensure you describe additional experiments that were carried out and include a detailed rebuttal of any criticisms or requested revisions that you disagreed with. Please also ensure that your revised manuscript conforms to the journal style, which can be found in the Instructions for Authors on the journal homepage. If the data and code has been modified in the revision process please be sure to update the public versions of this too.

The due date for submitting the revised version of your article is 06 Dec 2023.

I look forward to receiving your revised manuscript soon.

Best wishes,

Hongling Zhou
GigaScience
https://aus01.safelinks.protection.outlook.com/?url=http%3A%2F%2Fwww.gigasciencejournal.com%2F&data=05%7C01%7Cc.archibald%40deakin.edu.au%7Cb83f42e4d5064d41996308dbb018b752%7Cd02378ec168846d585401c28b5f470f6%7C0%7C0%7C638297393738149435%7CUnknown%7CTWFpbGZsb3d8eyJWIjoiMC4wLjAwMDAiLCJQIjoiV2luMzIiLCJBTiI6Ik1haWwiLCJXVCI6Mn0%3D%7C3000%7C%7C%7C&sdata=nq1%2BnH5afs0LilMaNqUnsiJLEIhZr4%2BIytT9lTJoYfQ%3D&reserved=0

Dear Hongling Zhou,

Thank you for these three constructive reviews and your feedback on this manuscript, "Habitat suitability maps for Australian flora and fauna under CMIP6 climate scenarios" (GIGA-D-23-00183). We note the 12 key points of feedback raised by the reviewers,

and have addressed them line-by-line.

We have added additional information about the occurrence point data used, and have addressed the concerns about sample bias by describing the use of our target group background points in more detail. We have acknowledged the comments on model validation raised by reviewer 1 and reviewer two and have discussed the limitations of using AUC to evaluate model performance [as well as added one additional indicator of model performance]. Reviewer 2 also emphasised to value of adding additional information about the practical uses of the data, which we have addressed in the discussion by adding two new sections on “Applications for landscape and species conservation”, and on “Applications in sustainability and natural capital accounting”. Overall, we have also greatly expanded the limitations and caveats section to ensure that these elements are discussed in as much detail as possible.

We are confident that these changes to the manuscript make this study a much more robust and useful contribution to the literature, and we look forward to your response. Please find detailed line-by-line changes below.

Sincerely,
Carla Archibald

Reviewer reports:

Reviewer #1:

Title: Habitat suitability maps for Australian flora and fauna under CMIP6 climate scenarios
MS#: GIGA-D-23-00183 Article type: Data Note

The maximum entropy technique was used by the authors to study the potential geographic distribution of thousands of terrestrial vertebrate and plant species and estimated the potential effects of climate change. This data note contains a wealth of informative material. Our understanding of the complex interactions between Australian species and their surroundings is improved by this painstaking and analytical work, which also provides essential insights into ecological management and conservation. I also value the thoroughness of the analysis they conducted.

Without a doubt, this is a highly time-consuming and laborious task, therefore I'll just mention the minor changes below, which are specifically connected to the work limitations and which should be mentioned in the discussion section.

Thank you for your thoughtful and positive feedback about our analysis and data. Your recognition of the value of this research in enhancing our understanding of Australian species and its relevance to conservation is greatly appreciated. Regarding the minor changes and limitations that you've identified, we have acknowledged such aspects in the in the paper to ensure transparency and robustness which we have also included below.

These include;

1.No doubt, MaxEnt perform really good with least number of occurrence points as well, please convey the minimum and maximum number of utilized occurrence points of the considered species in the text.

Thank you for this comment, it’s a good idea to include the range of occurrence points somewhere in the manuscript. So, what we have done is calculated a histogram of the total number of occurrence points across species. 95% of species have less than 1000 occurrence points, and there are 6 species (0.05%) that have less than 10 occurrence points. 580 species have more than 1000 occurrence points, and the species with the most occurrence points is the Willie wagtail (Rhipidura leucophrys) which has 78,503 occurrence points. The 25th quartile is 43 points, the median number of occurrence points is 123, and the 75th quartile is 410.

We have added the following text into the manuscript:

Powered by Editorial Manager® and ProduXion Manager® from Aries Systems Corporation

Line 92: “Across all species, the median number of occurrence points was 123 and the distribution of the number of occurrence points ranged based on the following quantiles: 0%=1, 25%= 43, 50%= 123, 75%= 410, 100%= 78,503 (Figure 2).”

Figure 2 Distribution of occurrence points (n) for species models.

2.Line 72-74; and 86-87: Did you clip the environmental data to Australian extent and utilized to extract 10,000 background points for all species? Or clipped the environmental data with different extent for each species based on minimum and maximum lat/long values of each species? Clarity is missing. Use of a large background especially for those species having least number of occurrence points growing in any unique microhabitat (and in a small geographic land area) influence AUC significantly.

Thank you for raising this point about how the background files were constructed, we recognise that we could have made this much clearer within our methods. To answer your point directly about whether we clipped the environmental data to Australian extent (based on your reference to Lines 86-87). We had provided this information about initial data manipulation and clipping already on:

Line 123: “All climate scenarios, bioclimatic variables were clipped to the extent of Australia prior to modelling.”

To build on this, we will include more detail on the background file as you have suggested (based on your references to Line 72-74 above). To generate the background files, we did not clip the environmental data further, rather we sampled the background points from only a subset of pixels across Australia based on the occurrence point distribution across taxonomic groups. To create the target group background files, we combined all occurrence points for all species within a taxonomic group, and then sampled the background points from this space. We did this at a taxonomic group level rather than at a species level. This approach of using a target group background file is a way for us to consider spatial biases in Australia.

To address this comment within the manuscript, we have added additional details within the methods section:

Line 98: “MaxEnt uses background sample points as pseudoabsences and recommends the use of target groups in sample selection to help overcome considered spatial biases (Phillips et al., 2009). To create the target group background files, we combined all occurrence points for all species within a taxonomic group and sampled the background points from this space. Each target group background file contained between 60,000 to 250,000 points depending on the taxonomic group, in which MaxEnt takes a subsample of 10,000 points.”

To address your final point about the use of a large background file for species with few occurrence points and this not being able to identify unique microhabitat in a small geographic land area we have added the following text into the methods and limitations section:

Line 473: “While we did use target background files to reduce spatial biases (Barber et al., 2022), there may still be limitations of this approach at the taxonomic group level, for example for small ranging species (Breiner et al., 2015). Taxonomic level grouping may still be too broad to adequately capture those species that are highly range restricted and require very specific micro-climate needs, therefore species-specific level grouping may help to overcome this. Background files that are too broad may adequately capture sampling biases or the true relationship between occurrence points and environmental predictors.”

3.As you targeted the terrestrial biota mainly, why NDVI or related measures were not considered to be used as important discriminatory variables? Such measures are very important for species residing in dense forests.

Powered by Editorial Manager® and ProduXion Manager® from Aries Systems Corporation

	We acknowledge that a range of variables like NDVI (Wen et al., 2015) and weather more broadly (Reside et al., 2010) can be important variables to define the distribution of species. However, many of the key studies we drew upon to inform variable selection for the models the habitat suitability of biodiversity in Australia under climate change did not use NDVI as an explanatory variable (Butt et al., 2013; Gallagher et al., 2019; Graham et al., 2019). Additionally, one major aspect of our study was future projections, and while we used future bioclimatic scenario data from WorldClim, to incorporate NDVI into this analysis, we would have had to model future changes in NDVI, and then use these future projected values within the Maxent analysis. While we did not do this as this was out of the scope of our specific study, many of the bioclimatic variables that we did use in the Maxent models have been reported to be significantly correlated with NDVI (Schwager & Berg, 2021). For example, average temperature, annual precipitation and precipitation seasonality (Ma et al., 2022). To address why we didn't not use why NDVI up-front in the paper we have added the following text within the Limitations:<br><br>Line 482: "In relation to the variables used, we were primarily guided by past efforts that model the suitability of areas across Australia for many species (Butt et al., 2013; Gallagher et al., 2019; Graham et al., 2019), however this approach obviously overlooks some variables that can be import to model suitability. For example, we did not consider variables such as the normalized difference vegetation index (NDVI) (Wen et al., 2015), land use (Lentini & Wintle, 2015), weather (Reside et al., 2010), or detailed information about vegetation structure or extreme events like fire (Eyre et al., 2022).Thus, our recommendation is for the users of this data to consider whether the variables used to model habitat suitability in this study is compatible with the species of interest, or whether additional information is required. This will likely be the case if the user is interested in a more fine-scale application of the data, for example at the single species or local level, as this data is best suited for macro-level analyses and applications."<br><br>4.To minimize the impact of sampling bias, a bias file (using species occurrence data and environment to estimate and develop a two-dimensional kernel density raster) for each species can be generated and used in MaxEnt distribution modelling. The inclusion of such bias files in the MaxEnt modelling effectively manipulate the background, and introduce the same spatial bias like that which exists in the presence data (Please see; https://aus01.safelinks.protection.outlook.com/?url=https%3A%2F%2Fdoi.org%2F10.3390%2Ff13050715&data=05%7C01%7Cc.archibald%40deakin.edu.au%7Cb83f42e4d5064d41996308dbb018b752%7Cd02378ec168846d585401c28b5f470f6%7C0%7C0%7C638297393738149435%7CUnknown%7CTWFpbGZsb3d8eyJWIjoiMC4wLjAwMDAiLCJQIjoiV2luMzIiLCJBTiI6Ik1haWwiLCJXVCI6Mn0%3D%7C3000%7C%7C%7C&sdata=HNgyy9z%2Fo8lLNLX%2B4wP%2B2SLaGniK5zB6X7kA8KmSwMs%3D&reserved=0).<br><br>Thank you for raising this point about spatial biases, we recognise that we could have made this much clearer within our methods. We considered spatial biases in this analysis though using a target group background files which we have addressed in your second comment about the target background files.<br><br>To address this comment within the manuscript, we have added additional details within the methods section:<br><br>Line 98: "MaxEnt uses background sample points as pseudoabsences and recommends the use of target groups in sample selection to help overcome considered spatial biases (Philips et al. 2009). To create the target group background files, we combined all occurrence points for all species within a taxonomic group and sampled the background points from this space. Each target group background file contained between 60,000 to 250,000 points depending on the taxonomic group, in which MaxEnt takes a subsample of 10,000 points."<br><br>5.Though, overall variable importance is communicated, different species have different environmental requirements. I am unable to access the relevant additional/supplementary material at the moment, and want to suggest that please

convey the variable importance for highly endangered species (if yet not included in the supplementary data).

The variable importance values for each variable are presented within the Maxent Report files for each species. Therefore, if readers would like to check the individual value for a variable for a species of interest this information on the variable importance is available for the user. Additionally, to your point about endangered species, these listings constantly change and update. So, in the table provided, rather than including the current listings, we have just included an up-dated scientific and common name column which can be joined to relevant listing databases when required by the user.

6.AUC is a widely used metric, but have several inherent flaws, especially for Gaussian point processes, such as MaxEnt. AUC and TSS are frequently criticized by the researchers:
a.AUC: a misleading measure of the predictive distribution models;
b.Without quality presence-absence data, discrimination metrics such as TSS can be misleading measures of model performance, hence, partial AUC-ROC might be relatively more appropriate measure.
Please include all the possible limitations of the work as mentioned above in the discussion section.

Thank you for raising this point about the importance of model validation and raising the challenges of using AUC and TSS to assess model performance. To address the first point about the AUC being a misleading measure, we have better clarified what the AUC assesses within in the Methods section. This ensures the reader is refreshed on what the AUC actual measures. To address the second point, and the comment more generally, about assessing model performance and the role and/or suitability of the AUC as an indicator of this, we have added the presentation of the Boyce Index, as well as expanded section about model validation within the limitations.

Model validation, from Line 211: “Model validation

Once variables were selected, models were re-run, and model performance was assessed based on the area under the curve (AUC, i.e., the area under the receiving operating curve (ROC) curve) and the Boyce index. The AUC is a widely used model validation metric used within the Maxent literature (Merow et al., 2013). The AUC metric measures the predictive accuracy of the model and represents the probability that a randomly selected occurrence point is ranked higher than a randomly selected background point. The Boyce index is another method that can be used to evaluate model performance and does so assessing the magnitude in which the model predictions differ from random distribution of the observed presences across the prediction gradients (Boyce et al., 2002; Hirzel et al., 2006a). The Boyce index value is represented by the Spearman rank correlation coefficient which assesses the increase in the Predicted/Expected (P/E) plot (Jiménez & Soberón, 2020).

The median AUC across all models was 0.97. AUC values of 0.7 or below indicates poor performance (Figure 2), 99.6% (n=10,566) of species have an AUC value above 0.7 AUC, and 0.4% (n=38) of species have an AUC value below the 0.7 threshold (33 birds, 4 vascular plants and 1 mammal). Boyce index values can vary from −1 to 1 and the median Boyce index across all models in this study was 0.97 (Figure 2). A Boyce index closer to 1 indicates that suitability predictions are consistent with the occurrence point distribution, and values of 0.5 or below generally indicate poor performance (Boyce et al., 2002; Hirzel et al., 2006a). 99.3% (n=10,509) of species had a value over 0.5, 0.65% (n=69) species had a value between 0.5 and 0 and 0.05% (n=5) species had a value below 0 (1 bird and 4 vascular plants).

We have also provided a scatter plot summary of AUC in relation to Boyce Index. Based on the 0.7 threshold for AUC and the 0.5 threshold for the Boyce index, we find that 98.99% of species meet both thresholds. We find that 0.69% (n= 73) meet the AUC threshold but not the Boyce Index threshold, 0.32% (n= 34) species meet the Boyce Index threshold but not the AUC threshold and 1 species did not meet either threshold (Brown Falcon, Falco berigora). Prior to using species data, please ensure you check the AUC and the Boyce index value which is contained within the species folder within the maxentResults.csv and the boyce_index_score.csv file.

Powered by Editorial Manager® and ProduXion Manager® from Aries Systems Corporation

Figure 2 From left to right the plots are the distribution of AUC values, the distribution of Boyce Index (BI) values and a scatter plot between AUC and BI values for species models. The median AUC and Boyce Index value is represented by the dashed vertical line. On the AUC plot the 0.7 threshold is presented using a solid vertical line. On the BI plot the 0.5 threshold is presented using a solid vertical line. These thresholds are also represented by solid lines on the scatter plot."

Finally, in the Limitations section we have also raised this concern:

Limitations, Line 513: "Finally, there is much contention around the best way to assess model performance of Maxent models beyond just the AUC, to approaches like the True Skill Statistic value (TSS), the kappa score and the Boyce Index (Allouche et al., 2006; Hirzel et al., 2006; Jiménez & Soberón, 2020; Valavi et al., 2022). We present the AUC and the Boyce Index and do not consider the thresholds for these indexes prior to creating the habitat suitability projections, therefore the user can assess the model performance for their species on interest when interpreting the data."

Reviewer #2:

The manuscript entitled "Habitat suitability maps for Australian flora and fauna under CMIP6 climate scenarios" uses a distribution modeling approach (i.e., Maxent) together with environmental variables to model and map current and future habitat suitability distributions of a large number of flora and fauna species. I enjoyed reading this work; it is nicely written and has proper structure. The drawn conclusions are coherent with the obtained results. This is an important paper that needs to be published to promote conservation and management actions in Australia. Although similar methodologies are common, the results of the study and the provided information could have useful implications for management and conservation actions.

Thank you very much for your positive feedback on our manuscript, "Habitat suitability maps for Australian flora and fauna under CMIP6 climate scenarios." We appreciate your kind words and recognition of the paper's structure, clarity, and the coherence of our conclusions with the obtained results. Your support for the publication of this work is greatly appreciated. We agree that while similar methodologies are prevalent, the unique results and information presented in our paper could indeed offer valuable insights for practical management and conservation actions. We have addressed the minor changes and limitations that you've identified below.

Abstract
- Please outline the objective of the study clearly.
We have edited this sentence to now read:

Abstract, Line 6: "The objective of this paper is to develop habitat suitability maps for Australian flora and fauna under different climate futures."

- Please add some information on how suitable habitats would change by comparing current areas with future areas.
We have now added some information on how suitable habitats may change, this section now reads:

Powered by Editorial Manager® and ProduXion Manager® from Aries Systems Corporation

Abstract, Line 13: “Conclusions: The spatial data supplied can help identify important and sensitive locations for species under various climate futures. Additionally, the supplied tabular data can provide insights into the impacts of climate change on biodiversity in Australia. These habitat suitability maps can be used as input data for landscape and conservation planning or species management, particularly under different climate change scenarios in Australia.”

Methods

Line 81-82: I suggest citing the GCMs.

Line 124: “We have added the references to each GCM, “Bioclimatic variables were downloaded for eight global climate models (GCMs): BCC-CSM2-MR (Wu et al., 2021), CNRM-CM6-1 (Voldoire et al., 2019), CNRM-ESM2-1 (Séférian et al., 2019), CanESM5 (Swart et al., 2019), GFDL-ESM4 (Krasting et al., 2018), IPSL-CM6A-LR (Boucher et al., 2020), MIROC-ES2L (Hajima et al., 2020), MIROC6 (Tatebe et al., 2018), MRI-ESM2-0 (Yukimoto et al., 2019)”

See the reference list at the end of this document for the full references.

Line 96: “Model fitting” Please outline the model setting of Maxent. Model parameterization is important, yet minimal information is provided.

Thank you for to this point about needing to clarify the model fitting section. To provide you with a clear understanding of the call here, we have presented the exact Maxent command line call below, which is also provided in the GitHub repository for this study.

Initial cross validation step:
java -mx2048m -jar /maxent.jar -e background.csv -s occurance.csv -o /species_folder nothreshold nowarnings novisible allowpartialdata replicates=10 nooutputgrids -r -a

Model fitting:
java -mx2048m -jar / maxent.jar -e /background.csv -s / occurance.csv -o /species_folder nothreshold nowarnings novisible nowriteclampgrid nowritemess allowpartialdata writeplotdata -P -J -r -a

To address this comment within the paper, we have edited the model fitting section to now read:

Line 154: “All habitat suitability models were fit in MaxEnt Version 3.4.1 using the command line. Maxent models were first run with 10 replicates (replicates=10) validated using a cross validation method to train the model and to compute model validation statistics. At this stage, habitat suitability values are calculated as values between 0 and 1 with no threshold applied and were later converted to values between 0 and 100. An example of the full Maxent model specification can be found in the GitHub repository affiliated with this paper. Important outputs of the MaxEnt modelling procedure include a .csv file containing statistical information to inform variable selection and model validation as well as the ‘lambdas file’, which is a text file containing the regression coefficients or lambdas fit by MaxEnt during modelling.”

Line 136: “AUC”, AUC alone is sometimes not sufficient to evaluate the performance of the model; other metrics such as Kappa and TSS are also important.

Thank you for raising this point about the importance of model validation and raising the challenges of using AUC and TSS to assess model performance. To address the first point about the AUC being a misleading measure, we have better clarify what the AUC assess within in the Methods section. This ensures the reader is refreshed on what the AUC actual measures:

Methods, Line 214: “Once variables were selected, models were re-run, and model performance was assessed based on the area under the curve (AUC, i.e., the area under the receiver operating curve (ROC) curve) and the Boyce index. The AUC is a widely used model validation metric used within the Maxent literature (Merow et al., 2013). The AUC metric measures the predictive accuracy of the model and represents

Powered by Editorial Manager® and ProduXion Manager® from Aries Systems Corporation

the probability that a randomly selected occurrence point is ranked higher than a randomly selected background point."

To address the second point, and the comment more generally, about assessing model performance and the role and/or suitability of the AUC as an indicator of this, we have added the presentation of the Boyce Index, as well as expanded section about model validation within the limitations.

Model validation, from Line 211: "Model validation

Once variables were selected, models were re-run, and model performance was assessed based on the area under the curve (AUC, i.e., the area under the receiving operating curve (ROC) curve) and the Boyce index. The AUC is a widely used model validation metric used within the Maxent literature (Merow et al., 2013). The AUC metric measures the predictive accuracy of the model and represents the probability that a randomly selected occurrence point is ranked higher than a randomly selected background point. The Boyce index is another method that can be used to evaluate model performance and does so assessing the magnitude in which the model predictions differ from random distribution of the observed presences across the prediction gradients (Boyce et al., 2002; Hirzel et al., 2006a). The Boyce index value is represented by the Spearman rank correlation coefficient which assesses the increase in the Predicted/Expected (P/E) plot (Jiménez & Soberón, 2020).

The median AUC across all models was 0.97. AUC values of 0.7 or below indicates poor performance (Figure 2), 99.6% (n=10,566) of species have an AUC value above 0.7 AUC, and 0.4% (n=38) of species have an AUC value below the 0.7 threshold (33 birds, 4 vascular plants and 1 mammal). Boyce index values can vary from −1 to 1 and the median Boyce index across all models in this study was 0.97 (Figure 2). A Boyce index closer to 1 indicates that suitability predictions are consistent with the occurrence point distribution, and values of 0.5 or below generally indicate poor performance (Boyce et al., 2002; Hirzel et al., 2006a). 99.3% (n=10,509) of species had a value over 0.5, 0.65% (n=69) species had a value between 0.5 and 0 and 0.05% (n=5) species had a value below 0 (1 bird and 4 vascular plants).

We have also provided a scatter plot summary of AUC in relation to Boyce Index. Based on the 0.7 threshold for AUC and the 0.5 threshold for the Boyce index, we find that 98.99% of species meet both thresholds. We find that 0.69% (n= 73) meet the AUC threshold but not the Boyce Index threshold, 0.32% (n= 34) species meet the Boyce Index threshold but not the AUC threshold and 1 species did not meet either threshold (Brown Falcon, Falco berigora). Prior to using species data, please ensure you check the AUC and the Boyce index value which is contained within the species folder within the maxentResults.csv and the boyce_index_score.csv file.

Figure 2 From left to right the plots are the distribution of AUC values, the distribution of Boyce Index (BI) values and a scatter plot between AUC and BI values for species models. The median AUC and Boyce Index value is represented by the dashed vertical line. On the AUC plot the 0.7 threshold is presented using a solid vertical line. On the BI plot the 0.5 threshold is presented using a solid vertical line. These thresholds are also represented by solid lines on the scatter plot."

Finally, in the Limitations section we have also raised this concern:

Powered by Editorial Manager® and ProduXion Manager® from Aries Systems Corporation

Limitations, Line 513: “Finally, there is much contention around the best way to assess model performance of Maxent models beyond just the AUC, to approaches like the True Skill Statistic value (TSS), the kappa score and the Boyce Index (Allouche et al., 2006; Hirzel et al., 2006; Jiménez & Soberón, 2020; Valavi et al., 2022). We present the AUC and the Boyce Index and do not consider the thresholds for these indexes prior to creating the habitat suitability projections, therefore the user can assess the model performance for their species on interest when interpreting the data.”

Line 238: “subtracted from one another to accentuate areas”. Reporting some information on how the suitability areas would change is suggested.

Thank you for this point, we will clarify where we have presented information on how suitability areas would change over time. We have slightly clarified the wording to ensure clarity, and have changed to:

Lines 354: “To calculate spatial changes in habitat suitability through time, historical suitability maps can be subtracted from future suitability maps to spatially accentuate locations that have changed in habitat suitability (i.e., improved in suitability or declined in suitability) across time periods.”

Discussion
A brief paragraph on the implications of the applied technique and the huge data could be useful. In addition, how the applied technique and provided data aid in establishing priority zones for conservation action is suggested.
https://doi.org/10.1016/j.ecoinf.2022.101930

Thank you for raising the need to better emphasise the use potential in the context of additional applied techniques to work with the data. To address this comment, we firstly wanted to point to the purpose of the “Re-use potential” potential sections and the role of the “Spatial resolution of data”, “Species-level data summary” and the “Spatial changes over time” sections to describe the initial reuse potential of the data. To extend these sections and provide additional recommendations on reuse potential we will better emphasise the use and various potential applied techniques to work with the data in the Discussion.
Discussion, from Line 392:
“Applications for landscape and species conservation
This spatial and tabular dataset is ideal for users that would like to understand how the habitat suitability of areas for species is predicted to change over time or under different climate scenarios. For example, at the landscape level, these habitat suitability maps can be combined into a general biodiversity layer to evaluate how habitat suitability more generally changes over time (Figure 6) or over space and time (Figure 5) (Hama & Khwarahm, 2023). This data can then be utilized in applications such as spatial prioritizations using such tools as Zonation (Minin et al., 2014) or Marxan (Watts et al., 2009) to guide spatial conservation priorities in Australia (Maxwell et al., 2019; Summers et al., 2012b). Therefore, can be useful to inform conservation (e.g., where to establish new protected areas), restoration or monitoring plans in areas which are suitable for biodiversity, or are predicted to lose or gain suitable areas for biodiversity.

At the species level, this data set can be used to support conservation actions for species of interest (e.g., threatened species, iconic species, endemic species). The tabular data can be used to systematically identify species of interest based on the way climate change is anticipated to impact the species. Or could be used to inform processes such as threatened species listing (IUCN, 2022). Spatial information about species could also be useful to compare the long-term suitability of areas for threatened species under climate change to inform present day decision-making and species management (Harley, 2023; Hawke et al., 2020). Could be paired with other types of data to assess the impacts of climate change on species (Eyre et al., 2022). Or could inform boarder scale biodiversity conservation analyses (Engert et al., 2023).
Applications in sustainability and natural capital accounting
Biodiversity forms a foundation of broader sustainability ideals, therefore, to measure progress towards sustainability, conservation or corporate goals spatial data on biodiversity can serve as an important input infomration to the creation of metrics

Powered by Editorial Manager® and ProduXion Manager® from Aries Systems Corporation

(Lamb et al., 2009; Watermeyer et al., 2021). Biodiversity indicators like the species richness, or more complex indicators like the Species Threat Abatement and Restoration metric (STAR) (Mair et al., 2021) or the biodiversity intactness index (BII) (Biggs & Scholes, 2005) all draw from species layers as input data. Feeding the habitat suitability maps generated in this study into biodiversity layers and into broader sustainability models or assessments can improve the consideration of biodiversity against other environmental or social values. This may include initiatives such as land use planning, or land use change modelling (Gao & Bryan, 2017; Stock et al., 2013). Additionally, as many businesses are transitioning towards 'nature positive' the use of biodiversity to monitor business impacts and progress towards nature positive is necessary. The habitat suitability maps generated in this study can be used to represent key species or biodiversity within natural capital within frameworks such as the System of Environmental-Economic Accounting (SEEA) framework (UNEP et al., 2015), or could be used within sustainability assessments such as 'foot printing' to updates the biodiversity input data (Halpern et al., 2022; Hoang et al., 2023; Irwin & Geschke, 2023), or could be used to help assess organisational impacts or dependencies to biodiversity or to specific species within frameworks like the Taskforce on Nature-Related Financial Disclosures (TNFD) (TNFD, 2023)."

Reviewer #3:

This manuscript presents habitat suitability of Australian flora and fauna under future climate change. The authors produced habitat suitability maps under RCP2.6-SSP1, RCP4.5-SSP2, RCP7.0-SSP3 and RCP8.5-SSP5 climate futures for 1,382 terrestrial vertebrates and 9,251 vascular Plants. The research can be used for landscape and conservation planning or species management, particularly under different climate change scenarios in Australia contribute towards evidence-based decision-making regarding demarcation of protected areas in Iran. Overall, the manuscript is well written with sufficient presentation of research aims, methodologies, key results and outcomes. I recommend this manuscript for publication in GigaScience.

Thank you for this overwhelmingly supportive review. We appreciate your shared vision of the potential importance and role of this data in real-world applications of landscape and conservation planning or species management in Australia.

Reference List

Allouche, O., Tsoar, A., & Kadmon, R. (2006). Assessing the accuracy of species distribution models: prevalence, kappa and the true skill statistic (TSS). Journal of Applied Ecology, 43(6), 1223–1232. https://doi.org/10.1111/j.1365-2664.2006.01214.x
Barber, R. A., Ball, S. G., Morris, R. K. A., & Gilbert, F. (2022). Target-group backgrounds prove effective at correcting sampling bias in Maxent models. Diversity and Distributions, 28(1), 128–141. https://doi.org/10.1111/ddi.13442
Biggs, R., & Scholes, R. J. (2005). A biodiversity intactness index. Nature, 434(7029), 45–49. http://go.galegroup.com/ps/i.do?id=GALE%7CA185471773&v=2.1&u=ntu&it=r&p=AONE&sw=w
Boucher, O., Servonnat, J., Albright, A. L., Aumont, O., Balkanski, Y., Bastrikov, V., Bekki, S., Bonnet, R., Bony, S., Bopp, L., Braconnot, P., Brockmann, P., Cadule, P., Caubel, A., Cheruy, F., Codron, F., Cozic, A., Cugnet, D., D'Andrea, F., … Vuichard, N. (2020). Presentation and Evaluation of the IPSL-CM6A-LR Climate Model. Journal of Advances in Modeling Earth Systems, 12(7), 1–52. https://doi.org/10.1029/2019MS002010
Breiner, F. T., Guisan, A., Bergamini, A., & Nobis, M. P. (2015). Overcoming limitations of modelling rare species by using ensembles of small models. Methods in Ecology and Evolution, 6(10), 1210–1218. https://doi.org/10.1111/2041-210X.12403
Butt, N., Pollock, L. J., & Mcalpine, C. A. (2013). Eucalypts face increasing climate stress. Ecology and Evolution, 3(15), 5011–5022. https://doi.org/10.1002/ece3.873
Engert, J. E., Pressey, R. L., & Adams, V. M. (2023). Threatened fauna protections compromised by agricultural interests in Australia. Conservation Letters. https://doi.org/10.1111/conl.12975
Eyre, A. C., Briscoe, N. J., Harley, D. K. P., Lumsden, L. F., McComb, L. B., & Lentini, P. E. (2022). Using species distribution models and decision tools to direct surveys and identify potential translocation sites for a critically endangered species. Diversity and

Distributions, 28(4), 700–711. https://doi.org/10.1111/ddi.13469
Gallagher, R. V., Allen, S., & Wright, I. J. (2019). Safety margins and adaptive capacity of vegetation to climate change. Scientific Reports, 9(1), 1–11. https://doi.org/10.1038/s41598-019-44483-x
Gao, L., & Bryan, B. A. (2017). Finding pathways to national-scale land-sector sustainability. Nature, 544(7649), 217–222. https://doi.org/10.1038/nature21694
Graham, E. M., Reside, A. E., Atkinson, I., Baird, D., Hodgson, L., James, C. S., & VanDerWal, J. J. (2019). Climate change and biodiversity in Australia: a systematic modelling approach to nationwide species distributions. Australasian Journal of Environmental Management, 26(2), 112–123. https://doi.org/10.1080/14486563.2019.1599742
Hajima, T., Watanabe, M., Yamamoto, A., Tatebe, H., Noguchi, M. A., Abe, M., Ohgaito, R., Ito, A., Yamazaki, D., Okajima, H., Ito, A., Takata, K., Ogochi, K., Watanabe, S., & Kawamiya, M. (2020). Development of the MIROC-ES2L Earth system model and the evaluation of biogeochemical processes and feedbacks. Geoscientific Model Development, 13(5), 2197–2244. https://doi.org/10.5194/gmd-13-2197-2020
Halpern, B. S., Frazier, M., Verstaen, J., Rayner, P. E., Clawson, G., Blanchard, J. L., Cottrell, R. S., Froehlich, H. E., Gephart, J. A., Jacobsen, N. S., Kuempel, C. D., McIntyre, P. B., Metian, M., Moran, D., Nash, K. L., Többen, J., & Williams, D. R. (2022). The environmental footprint of global food production. Nature Sustainability, 5(12), 1027–1039. https://doi.org/10.1038/s41893-022-00965-x
Hama, A. A., & Khwarahm, N. R. (2023). Predictive mapping of two endemic oak tree species under climate change scenarios in a semiarid region: Range overlap and implications for conservation. Ecological Informatics, 73, 101930. https://doi.org/10.1016/j.ecoinf.2022.101930
Harley, D. (2023). Seven urgent actions to prevent the extinction of the critically endangered Leadbeater's possum (Gymnobelideus leadbeateri). Pacific Conservation Biology, 29(5), 387–395. https://doi.org/10.1071/PC22021
Hawke, T., Bino, G., Kingsford, R. T., Grant, T., Griffiths, J., Weeks, A., Tingley, R., Mccoll-Gausden, E., Serena, M., Williams, G., Brunt, T., Mijangos, L., Sherwin, W., & Noonan, J. (2020). A national assessment of the conservation status of the platypus.
Hirzel, A. H., Le Lay, G., Helfer, V., Randin, C., & Guisan, A. (2006). Evaluating the ability of habitat suitability models to predict species presences. Ecological Modelling, 199(2), 142–152. https://doi.org/10.1016/j.ecolmodel.2006.05.017
Hoang, N. T., Taherzadeh, O., Ohashi, H., Yonekura, Y., Nishijima, S., Yamabe, M., Matsui, T., Matsuda, H., Moran, D., & Kanemoto, K. (2023). Mapping potential conflicts between global agriculture and terrestrial conservation. Proceedings of the National Academy of Sciences of the United States of America, 120(23). https://doi.org/10.1073/pnas.2208376120
Irwin, A., & Geschke, A. (2023). A consumption-based analysis of extinction risk in Australia. Conservation Letters, 16(3). https://doi.org/10.1111/conl.12942
IUCN. (2022). The IUCN Red List of Threatened Species (IUCN). http://www.iucnredlist.org
Jiménez, L., & Soberón, J. (2020). Leaving the area under the receiving operating characteristic curve behind: An evaluation method for species distribution modelling applications based on presence-only data. Methods in Ecology and Evolution, 11(12), 1571–1586. https://doi.org/10.1111/2041-210X.13479
Krasting, J. P., John, J. G., Blanton, C., McHugh, C., Nikonov, S., Radhakrishnan, A., Rand, K., Zadeh, N. T., Balaji, V., Durachta, J., Dupuis, C., Menzel, R., Robinson, T., Underwood, S., Vahlenkamp, H., Dunne, K. A., Gauthier, P. P., Ginoux, P., Griffies, S. M., … Zhao, M. (2018). NOAA-GFDL GFDL-ESM4 model output prepared for CMIP6 CMIP.
Lamb, E. G., Bayne, E., Holloway, G., Schieck, J., Boutin, S., Herbers, J., & Haughland, D. L. (2009). Indices for monitoring biodiversity change: Are some more effective than others? Ecological Indicators, 9(3), 432–444. https://doi.org/10.1016/j.ecolind.2008.06.001
Lentini, P. E., & Wintle, B. A. (2015). Spatial conservation priorities are highly sensitive to choice of biodiversity surrogates and species distribution model type. Ecography, 38(11), 1101–1111. https://doi.org/10.1111/ecog.01252
Ma, B., Zeng, W., Hu, G., Cao, R., Cui, D., & Zhang, T. (2022). Normalized difference vegetation index prediction based on the delta downscaling method and back-propagation artificial neural network under climate change in the Sanjiangyuan region, China. Ecological Informatics, 72, 101883. https://doi.org/10.1016/j.ecoinf.2022.101883

Powered by Editorial Manager® and ProduXion Manager® from Aries Systems Corporation

Mair, L., Bennun, L. A., Brooks, T. M., Butchart, S. H. M., Bolam, F. C., Burgess, N. D., Ekstrom, J. M. M., Milner-Gulland, E. J., Hoffmann, M., Ma, K., Macfarlane, N. B. W., Raimondo, D. C., Rodrigues, A. S. L., Shen, X., Strassburg, B. B. N., Beatty, C. R., Gómez-Creutzberg, C., Iribarrem, A., Irmadhiany, M., … McGowan, P. J. K. (2021). A metric for spatially explicit contributions to science-based species targets. Nature Ecology and Evolution, 5(6), 836–844. https://doi.org/10.1038/s41559-021-01432-0
Maxwell, S. L., Reside, A., Trezise, J., McAlpine, C. A., & Watson, J. E. (2019). Retention and restoration priorities for climate adaptation in a multi-use landscape. Global Ecology and Conservation, 18, e00649. https://doi.org/10.1016/j.gecco.2019.e00649
Minin, E., Veach, V., Lehtomäki, J., Pouzols, F. M., & Moilanen, A. (2014). A quick introduction to Zonation. Version 1 (for Zv4). User Manual. 1, 1–30. http://cbig.it.helsinki.fi/files/zonation/Z_quick_intro_manual_B5_final_3.pdf
Phillips, S. J., Dudík, M., Elith, J., Graham, C. H., Lehmann, A., Leathwick, J., & Ferrier, S. (2009). Sample selection bias and presence-only distribution models: Implications for background and pseudo-absence data. Ecological Applications, 19(1), 181–197. https://doi.org/10.1890/07-2153.1
Reside, A. E., Vanderwal, J. J., Kutt, A. S., & Perkins, G. C. (2010). Weather, Not Climate, Defines Distributions of Vagile Bird Species. PLoS ONE, 5(10), 1–9. https://doi.org/10.1371/journal.pone.0013569
Schwager, P., & Berg, C. (2021). Remote sensing variables improve species distribution models for alpine plant species. Basic and Applied Ecology, 54, 1–13. https://doi.org/10.1016/j.baae.2021.04.002
Séférian, R., Nabat, P., Michou, M., Saint-Martin, D., Voldoire, A., Colin, J., Decharme, B., Delire, C., Berthet, S., Chevallier, M., Sénési, S., Franchisteguy, L., Vial, J., Mallet, M., Joetzjer, E., Geoffroy, O., Guérémy, J. F., Moine, M. P., Msadek, R., … Madec, G. (2019). Evaluation of CNRM Earth System Model, CNRM-ESM2-1: Role of Earth System Processes in Present-Day and Future Climate. Journal of Advances in Modeling Earth Systems, 11(12), 4182–4227. https://doi.org/10.1029/2019MS001791
Stock, F., Bryan, B., Nolan, M., Graham, P., Connor, J., Dunstall, S., Ernst, A., Stock, F., Nolan, M., Bryan, B., Graham, P., Connor, J., Dunstall, S., & Ernst, A. (2013). LUTO - Land Use Trade-Offs National modeling of land use choices and pathways.
Swart, N. C., Cole, J. N. S., Kharin, V. V., Lazare, M., Scinocca, J. F., Gillett, N. P., Anstey, J., Arora, V., Christian, J. R., Hanna, S., Jiao, Y., Lee, W. G., Majaess, F., Saenko, O. A., Seiler, C., Seinen, C., Shao, A., Sigmond, M., Solheim, L., … Winter, B. (2019). The Canadian Earth System Model version 5 (CanESM5.0.3). Geoscientific Model Development, 12(11), 4823–4873. https://doi.org/10.5194/gmd-12-4823-2019
Tatebe, H., Ogura, T., Nitta, T., Komuro, Y., Ogochi, K., Takemura, T., Sudo, K., Sekiguchi, M., Abe, M., Saito, F., Chikira, M., Watanabe, S., Mori, M., Hirota, N., Kawatani, Y., Mochizuki, T., Yoshimura, K., Takata, K., O'ishi, R., … Kimoto, M. (2018). Description and basic evaluation of simulated mean state, internal variability, and climate sensitivity in MIROC6. Geoscientific Model Development Discussions, 1–92. https://doi.org/10.5194/gmd-2018-155
TNFD. (2023). Recommendations of the Taskforce on Nature-related Financial Disclosures.
UNEP, UNSD, CBD, & NORAD. (2015). SEEA Experimental Ecosystem Accounting: Technical Recommendations (Issue December).
Valavi, R., Guillera-Arroita, G., Lahoz-Monfort, J. J., & Elith, J. (2022). Predictive performance of presence-only species distribution models: a benchmark study with reproducible code. Ecological Monographs, 92(1). https://doi.org/10.1002/ecm.1486
Voldoire, A., Saint-Martin, D., Sénési, S., Decharme, B., Alias, A., Chevallier, M., Colin, J., Guérémy, J. F., Michou, M., Moine, M. P., Nabat, P., Roehrig, R., Salas y Mélia, D., Séférian, R., Valcke, S., Beau, I., Belamari, S., Berthet, S., Cassou, C., … Waldman, R. (2019). Evaluation of CMIP6 DECK Experiments With CNRM-CM6-1. Journal of Advances in Modeling Earth Systems, 11(7), 2177–2213. https://doi.org/10.1029/2019MS001683
Watermeyer, K. E., Guillera-Arroita, G., Bal, P., Burgass, M. J., Bland, L. M., Collen, B., Hallam, C., Kelly, L. T., McCarthy, M. A., Regan, T. J., Stevenson, S., Wintle, B. A., & Nicholson, E. (2021). Using decision science to evaluate global biodiversity indices. Conservation Biology, 35(2), 492–501. https://doi.org/10.1111/cobi.13574
Watts, M. E., Ball, I. R., Stewart, R. S., Klein, C. J., Wilson, K., Steinback, C., Lourival, R., Kircher, L., & Possingham, H. P. (2009). Marxan with Zones: Software for optimal conservation based land- and sea-use zoning. Environmental Modelling and Software, 24(12), 1513–1521. https://doi.org/10.1016/j.envsoft.2009.06.005

	Wen, L., Saintilan, N., Yang, X., Hunter, S., & Mawer, D. (2015). MODIS NDVI based metrics improve habitat suitability modelling in fragmented patchy floodplains. Remote Sensing Applications: Society and Environment, 1, 85–97. https://doi.org/10.1016/j.rsase.2015.08.001<br>Wu, T., Yu, R., Lu, Y., Jie, W., Fang, Y., Zhang, J., Zhang, L., Xin, X., Li, L., Wang, Z., Liu, Y., Zhang, F., Wu, F., Chu, M., Li, J., Li, W., Zhang, Y., Shi, X., Zhou, W., … Hu, A. (2021). BCC-CSM2-HR: A high-resolution version of the Beijing Climate Center Climate System Model. Geoscientific Model Development, 14(5), 2977–3006. https://doi.org/10.5194/gmd-14-2977-2021<br>Yukimoto, S., Kawai, H., Koshiro, T., Oshima, N., Yoshida, K., Urakawa, S., Tsujino, H., Deushi, M., Tanaka, T., Hosaka, M., Yabu, S., Yoshimura, H., Shindo, E., Mizuta, R., Obata, A., Adachi, Y., & Ishii, M. (2019). The meteorological research institute Earth system model version 2.0, MRI-ESM2.0: Description and basic evaluation of the physical component. Journal of the Meteorological Society of Japan, 97(5), 931–965. https://doi.org/10.2151/jmsj.2019-051
**Additional Information:**	
**Question**	**Response**
Are you submitting this manuscript to a special series or article collection?	No
**Experimental design and statistics**<br><br>Full details of the experimental design and statistical methods used should be given in the Methods section, as detailed in our Minimum Standards Reporting Checklist. Information essential to interpreting the data presented should be made available in the figure legends.<br><br>Have you included all the information requested in your manuscript?	Yes
**Resources**<br><br>A description of all resources used, including antibodies, cell lines, animals and software tools, with enough information to allow them to be uniquely identified, should be included in the Methods section. Authors are strongly encouraged to cite Research Resource Identifiers (RRIDs) for antibodies, model organisms and tools, where possible.<br><br>Have you included the information requested as detailed in our Minimum Standards Reporting Checklist?	Yes

Powered by Editorial Manager® and ProduXion Manager® from Aries Systems Corporation

**Availability of data and materials**<br><br>All datasets and code on which the conclusions of the paper rely must be either included in your submission or deposited in publicly available repositories (where available and ethically appropriate), referencing such data using a unique identifier in the references and in the “Availability of Data and Materials” section of your manuscript.<br><br>Have you have met the above requirement as detailed in our Minimum Standards Reporting Checklist?	No
If not, please give reasons for any omissions below.<br><br>as follow-up to "**Availability of data and materials**<br><br>All datasets and code on which the conclusions of the paper rely must be either included in your submission or deposited in publicly available repositories (where available and ethically appropriate), referencing such data using a unique identifier in the references and in the “Availability of Data and Materials” section of your manuscript.<br><br>Have you have met the above requirement as detailed in our Minimum Standards Reporting Checklist?<br><br>"	I am also intending to upload the data to GigaDB titled "Supporting data for "Habitat suitability maps for Australian flora and fauna under CMIP6 climate scenarios". Therefore, I have not provided the link to the GigaDB in the Data Note, but I intend to add this when possible. I have included an excel file in the Supplementary Material including the files names of each file. The GitHub page is available, and a link is included in the manuscript.

Manuscript
Click here to access/download;Manuscript;GIGA-D-23-00183_Manuscript[80]_SE_v2.docx

Habitat suitability maps for Australian flora and fauna under CMIP6 climate scenarios

Carla L. Archibald[1*], David M. Summers[2], Erin M. Graham[3], Brett A. Bryan[1]

[1] School of Life & Environmental Science, Deakin University, Melbourne Burwood Campus, Victoria, Australia

[2] UniSA Business, The University of South Australia, Adelaide, Australia

[3] eResearch Centre, James Cook University, Townsville, Australia

* Corresponding author: c.archibald@deakin.edu.au

ORCID iDs:

Carla Leigh Archibald [0000-0003-1640-8396]; David M Summers [0000-0002-1872-5267]; Erin M Graham [0000-0002-8164-6975]; Brett A Bryan [0000-0003-4834-5641]

Abstract

Background: Spatial information about the location and suitability of areas for native plant and animal species under different climate futures is an important input to land use and conservation planning and management. Australia, renowned for its abundant species diversity and endemism, often relies on modelled data to assess species distributions due to the country's vast size and the challenges associated with conducting on-ground surveys on such a large scale. The objective of this paper is to develop habitat suitability maps for Australian flora and fauna under different climate futures. **Results**: Using MaxEnt, we produced Australia-wide habitat suitability maps under RCP2.6-SSP1, RCP4.5-SSP2, RCP7.0-SSP3 and RCP8.5-SSP5 climate futures for 1,382 terrestrial vertebrates and 9,251 vascular plants vascular plants at 5km^2 for open access. This represents 60% of all Australian mammal species, 77% of amphibian species, 50% of reptile species, 71% of bird species and 44% of vascular plant species. We also include tabular data which includes summaries of total quality-weighted habitat area of species under different climate scenarios and time periods. **Conclusions**: The spatial data supplied can help identify important and sensitive locations for species under various climate futures. Additionally, the supplied tabular data can provide insights into the impacts of climate change on biodiversity in Australia. These habitat suitability maps can be used as input data for landscape and conservation planning or species management, particularly under different climate change scenarios in Australia.

Data Description

Introduction

Rich spatial and temporal information about the effect of climatic and environmental change on species distributions is necessary to ensure robust species management and conservation policy more broadly [1–4]. Identifying areas where species occur now, as well as areas which may be suitable in the future, is a crucial aspect of decision making under uncertainty [5]. The availability of resources for conservation, including financial, staffing and land availability, is limited and exacerbates the challenge of conservation planning during climate change [3]. These constraints have sparked the need for more strategic landscape and conservation planning methods, such as spatial prioritization, to identify the most effective conservation solutions [6]. Spatial information on where species are now and where suitable areas may be in the future is the foundation of efficient planning for conservation action, particularly in areas where local conditions are more sensitive to climate change [5].

Australia is a hyper-diverse country with high levels of species endemism [7,8]. Unfortunately, Australia also has some of the highest recorded numbers of contemporary extinctions worldwide and more than 1900 species and ecological communities are under threat [9,10]. Given the extensive and severe range and population declines of many threatened species [10–12], many more species are also predicted to have a high risk of extinction in the future [13]. To ensure the conservation of Australia's unique biodiversity, identifying and protecting important areas for species such as climate refugia is key to planning for resilience and adaptive capacity [14]. To fulfill this task, underlying data on species location and the habitat suitability of areas for species under different climate futures is required.

There are many ways to assess suitable areas for species, and one popular approach is to use the maximum entropy method (henceforth, MaxEnt). MaxEnt is a niche-based general-purpose machine learning method with a simple and precise mathematical formulation which is particularly well-suited for species distribution modelling with presence-only data [15,16]. Generating MaxEnt models for individual species at continental scales presents challenges around the processing and storage of large volumes of data. Graham et al. (2019) developed a comprehensive spatial dataset of 1872 terrestrial and freshwater vertebrate species distributions using the Intergovernmental Panel on Climate Change's (IPCC) Coupled Model Intercomparison Project 3 (CMIP3) future climate projections [17] and made them freely available through a web-based portal known as 'CliMAS'. Although the CliMAS models led to many applied outcomes [18,19], the website was retired in 2020, in recognition of the fact that there have been two major updates by the IPCC and the current projections are based on CMIP6. For conservation planning to progress, an improved and enlarged suite of freely available spatial data, based on up-to-date climate projections and extended for a much broader range of species including vascular plants, is needed.

We developed habitat suitability maps for Australian flora and fauna under different climate futures using a MaxEnt approach. We produced freely accessible Australia-wide habitat suitability maps for 1,441 terrestrial vertebrates and 9,251 vascular plants. This represents 60%

of all Australian mammal species, 77% of amphibian species, 50% of reptile species, 71% of bird species and 44% of vascular plant species. We fit these models using 7 bioclimatic variables and 11 soil and landscape variables under 4 climate scenarios, 8 General Circulation Models (GCMs) and 1 ensemble average, and 5 time periods. These habitat suitability maps are best used as input data to represent species or biodiversity values for conservation planning and assessment, particularly under climate change in Australia.

Methods

The workflow for this study was adapted from the CliMAS project [20] (Figure 1). The first step involved compiling and collecting the input data which consisted of occurrence point data and environmental variables. We then used MaxEnt to fit models of habitat suitability using climate, soil and landscape variables. We used a variable selection procedure which considered the statistical and ecological importance of variables to refine the predictor variables as well as validating the models. We then used the lambda files produced in the model fitting step to project species habitat suitability under future climate scenarios.

[INSERT FIGURE 1 HERE]

Figure 1 *Workflow of the MaxEnt modelling procedure. Input data is represented as green, variable selection procedure is represented as purple, MaxEnt modelling procedure is represented as grey and the output files are represented as orange.*

Input data

Species occurrence points

Species occurrence records which were used to fit the historical climate models were sourced from the Australian Atlas of Living Australia (ALA) [21], the Queensland Museum, and CSIRO. Vascular plant occurrence point data were acquired through from the Queensland Museum. Vertebrate species occurrence were records acquired through ALA went through an additional data cleaning process prior to modelling [21]. We used the points originally applied in the CliMAS project as of 2012 for vertebrates, and the vascular plant points that were compiled for the CliMAS project but never modelled. Through these sources we obtained occurrence point data for 197 mammals (60% coverage), 523 birds (71% coverage), 530 reptiles (50%), 191 amphibians (77%) and 9,200 vascular plants (44% coverage). Across all species, the median number of occurrence points was 123 and the distribution of the number of occurrence points ranged based on the following quantiles: 0%=1, 25%= 43, 50%= 123, 75%= 410, 100%= 78,503 (Figure 2).

[INSERT FIGURE 2 HERE]

Figure 2 *Distribution of occurrence points (n) for species models.*

MaxEnt uses background sample points as pseudoabsences and recommends the use of target groups in sample selection to help overcome considered spatial biases [22,23]. To create the target group background files, we combined all occurrence points for all species within a taxonomic group and sampled the background points from this space. Each target group background file contained between 60,000 to 250,000 points depending on the taxonomic group, in which MaxEnt takes a subsample of 10,000 points.

Environmental variables

We used a combination of bioclimatic, soil and landscape variables as predictors to fit the MaxEnt models. For the climate variables, we downloaded spatial data at a $5km^2$ resolution on historical and future CMIP6 modelled bioclimatic variables through the WorldClim database[24]. Bioclimatic variables summarise monthly temperature and rainfall values into 19 more biologically meaningful variables (Table 1). Bioclimatic variables were downloaded for eight GCMs: BCC-CSM2-MR [25], CNRM-CM6-1 [26], CNRM-ESM2-1 [27], CanESM5 [28], GFDL-ESM4 [29], IPSL-CM6A-LR [30], MIROC-ES2L [31], MIROC6 [32], MRI-ESM2-0 [33], for four shared socioeconomic (SSP) [34] and representative concentration pathway (RCP) combinations: RCP2.6-SSP1, RCP4.5-SSP2, RCP7.0-SSP3 and RCP8.5-SSP5 and 5 time-periods (1990, 2030, 2050, 2070 and 2090). As we did not have access to the following two files: IPSL-CM6A-LR SSP2-4.5 2030 and MRI-ESM2-0 SSP5-8.5 2030, we linearly interpolated values. All climate scenarios, bioclimatic variables were clipped to the extent of Australia prior to modelling.

We downloaded 15 environmental variables from the Soil and Landscape Grid of Australia [35] to use as environmental predictors of habitat suitability. Additionally, we downloaded the Interim Biogeographic Regionalisation for Australia [36] (IBRA) as an indication of the inherent spatial differences in biome across Australia. Soil and landscape variables were clipped and masked to the extent of Australia and scaled to the same resolution as the bioclimatic data (Table 1).

MaxEnt modelling procedure

Model fitting

All habitat suitability models were fit in MaxEnt Version 3.4.1 using the command line. MaxEnt models were first run with 10 replicates (replicates=10) validated using a cross validation method to train the model and to compute model validation statistics. At this stage, habitat suitability values are calculated as values between 0 and 1 with no threshold applied and were later converted to values between 0 and 100. An example of the full MaxEnt model specification can be found in the GitHub or Zenodo repository affiliated with this paper [37]. Important outputs of the MaxEnt modelling procedure include a .csv file containing statistical information to inform variable selection and model validation as well as the ‘lambdas file’, which is a text file containing the regression coefficients or lambdas fit by MaxEnt during modelling.

Variable selection

The variables included in the final MaxEnt model runs were informed by analysing the variable contributions and importance percentages calculated using a full MaxEnt model run, information about variable complexity [38], as well as ecological knowledge based on several published models of terrestrial vertebrate and vascular plant climate and habitat suitability. The goal of variable selection was to reduce the number of predictor variables from the initial 35 variables chosen as potential environmental predictors to avoid overfitting. Although MaxEnt is robust to

multicollinearity among variables [39], including excessive numbers of predictors can affect the model's ability to make inferences outside of the training data.

We reviewed variables included within several Australian biodiversity modelling efforts of terrestrial vertebrates [20] and vascular plants [40,41]. We then performed a full MaxEnt model run which included the 35 variables described in the above section, for each species. We reviewed the importance of variables based on the average percent contribution and percent importance values across all species. The percent contribution is a measure of the contribution of each variable towards model fit after each iteration of the MaxEnt model, while the percent importance is a measure of the importance of each variable towards model fit for the final MaxEnt model. We also categorized bioclimatic variables based on complexity and favoured simple variables as they tended to be less correlated with one another [38].

This combined approach to variable selection resulted in 18 variables which moved through to the model fitting stage (Table 1): 7 bioclimatic variables and 11 soil and landscape variables. All bioclimatic variables selected for this study were included in CliMAS models [20] and similar modelling efforts for Australian plants [40], and all bioclimatic variables with the exception of BIO15 were considered to be simple climate variables [38] (Table 1). All bioclimatic variables except for BIO05 had high or moderate importance values in the full model. Similarly, we included additional soil and landscape variables [42] based on their use in recent biodiversity models [40], and we favoured soil and landscape variables that were simpler.

Table 1 *Summary of the bioclimatic, soil and landscape variable selected in the final MaxEnt model.*

[INSERT TABLE 1 HERE]

Model validation

Once variables were selected, models were re-run, and model performance was assessed based on the area under the curve (AUC, i.e., the area under the receiver operating curve (ROC) curve) and the Boyce Index. The AUC is a widely used model validation metric used within the MaxEnt literature [43]. The AUC metric measures the predictive accuracy of the model and represents the probability that a randomly selected occurrence point is ranked higher than a randomly selected background point. The Boyce Index is another method that can be used to evaluate model performance and does so assessing the magnitude in which the model predictions differ from random distribution of the observed presences across the prediction gradients [44,45]. The Boyce Index value is represented by the Spearman rank correlation coefficient which assesses the increase in the Prediction/Expected (P/E) plot [46].

The median AUC across all models was 0.97 and generally, AUC values of 0.7 or below indicates poor performance (Figure 3). We assess that 99.6% (n=10,566) of species have an AUC value above 0.7 AUC, and 0.4% (n=38) of species have an AUC value below the 0.7 threshold (33 birds, 4 vascular plants and 1 mammal). Boyce Index values can vary from −1 to 1 and we find that the median Boyce Index across all models in this study was 0.97 (Figure 3). A Boyce Index closer to 1 indicates that suitability predictions are consistent with the occurrence point distribution, and values of 0.5 or below generally indicate poor performance [44,45]. We

assess that 99.3% (n=10,509) of species have a value over 0.5, 0.65% (n=69) species had a value between 0.5 and 0 and 0.05% (n=5) species had a value below 0 (1 bird and 4 vascular plants).

We have also provided a scatter plot summary of AUC in relation to Boyce Index. Based on the 0.7 threshold for AUC and the 0.5 threshold for the Boyce Index, we find that 98.99% of species meet both thresholds. We find that 0.69% (n= 73) meet the AUC threshold but not the Boyce Index threshold, 0.32% (n= 34) species meet the Boyce Index threshold but not the AUC threshold and 1 species did not meet either threshold (Brown Falcon, *Falco berigora*). Prior to using species data, please ensure you check the AUC and the Boyce Index value which is contained within the species folder within the maxentResults.csv and the boyce_index_score.csv file.

[INSERT FIGURE 3 HERE]

Figure 3 *From left to right the plots are the distribution of AUC values, the distribution of Boyce Index (BI) values and a scatter plot between AUC and BI values for species models. The median AUC and Boyce Index value is represented by a dashed line. On the AUC plot the 0.7 threshold is presented using a solid vertical line. On the BI plot the 0.5 threshold is presented using a solid vertical line. These thresholds are also represented by solid lines on the scatter plot.*

Model projections

Using the best model selected in the model fitting procedure we projected species-level MaxEnt models under the future climate scenarios RCP2.6-SSP1, RCP4.5-SSP2, RCP7.0-SSP3 and RCP8.5-SSP5, 8 GCMs, for 1 historical time-period (1985) and 4 future time-periods (2030, 2050, 2070, 2090) using the lambda files produced in the model fitting step. Using the predicted habitat suitability data, we then calculated an ensemble average (mean), minimum and maximum habitat suitability (to capture model variance) across 8 GCMs for each species, climate scenario and time-period.

Geospatial calculations

To describe the patterns of habitat suitability across time in an accessible tabular format we calculated the total quality-weighted sum of habitat suitability for each species under different climate scenarios at each time period (Eq 1.). We first adjusted the resolution of the rasters to 1km^2, therefore the quality-weighted habitat area (*qwHA*) sum corresponds to the 'habitat area' in km^2. For example, if the probable habitat suitability in a cell is equal to 1, the cell is equivalent to 1km^2, whereas if the probable habitat suitability in a cell is equal to 0.3, the cell is equivalent to 0.3km^2. Noting that the quality-weighted habitat area is not equivalent to the realised area available for a species given ecological or land use constraints which can both influence habitat availability and suitability for species. The probability of habitat suitability (*p*) was summed across raster cells (*xy*), for each species (*j*), year (*y*) and climate scenario (*c*):

$$qwHA_{jyc} = \sum_{i=1}^{n} p_{jyc,xy}$$

(Eq 1.)

To describe how the patterns of habitat suitability may have changed across space under different climate scenarios or years, we summarised raster data for each species in multiple ways. For each taxonomic group (t) we calculated changes in habitat suitability (s) by subtracting future time periods and climate scenarios (yc) by historical climate niche (p^h). Where positive values indicate areas that increase in suitability in the future and negative values indicate areas that decrease in climate suitability in the future. We provide visual representation of this information in Figure 6, and included the absolute and proportional change in habitat area in the tabular summaries provided for species:

$$s_t^{yc} = p_t^h - p_t^{yc} \qquad \text{(Eq 2.)}$$

To spatially identify important areas of climate refugia which was done for Figure 6, we multiplied the historical habitat suitability matrix by the habitat suitability in each future climate scenario and year combination. For each the cell, the probability of habitat suitability values per cell (p), for each species (t), year (y) and climate scenario (c) were multiplied by the future habitat suitability. Cell values were then divided by 100, and the resulting cell value represents climate refugia (r) between 0 to 100.

$$r_t^{yc} = (p_t^h * p_t^{yc})/100 \qquad \text{(Eq 3.)}$$

Re-use potential

Code availability

For each species, MaxEnt models were run directly from the terminal using java and bash syntax and were ultimately executed using a 'Simple Linux Utility for Resource Management' (SLURM) workload manager on a high-performance Linux-based computer cluster. Additional modelling and geospatial analyses were processed using a shell file executed using SLURM on the computer cluster. The scripts used in to generate this data is available in the companion GitHub and Zonodo repository [37].

Dataset

Individual species' maps for historical and future minimum, mean and maximum ensembled habitat suitability, as well as the MaxEnt lambda file and summary reports produced in this study are publicly accessible for download on the open-access companion GigaDB database [47]. This dataset includes species-level historical (1970-2000 centered on 1990) and the future minimum, mean and maximum habitat suitability projections for 1,382 terrestrial vertebrates (182 amphibians, 487 birds, 178 mammals and 535 reptiles) and 9,251 vascular plants under 4 climate scenarios and 5 time periods, this data equates to 521,017 .tif raster files that are compressed using Lempel–Ziv–Welch (lzw) compression. Additionally, for each species we have included a .csv file which contains the total quality-weighted habitat areas (in km^2) for each species under each different climate scenario and time period. We have also consolidated these tables across all

species and included this tabular data. A complete list of the species for which habitat suitability maps were produced can be found in the companion GigaDB database.

Spatial resolution of data

This data is presented at 5km^2 resolution which is aligned with the climate data used as key inputs to the MaxEnt model. The data can be subsequently downscaled to finer resolutions, however assumptions will have to be made about how habitat suitability is distributed across cells. The current resolution of this data is best utilized to understand general trends across space and time. To demonstrate the resolution, we present the southern cassowary (*Casuarius casuarius*) which is known to occur in the Wet Tropics region of Queensland, Australia. Current suitable areas for the southern cassowary are predicted to occur between Townsville to Cooktown, with an isolated area around the Iron Range (Figure 4). Taking the most severe climate change scenario (RCP8.5 - SSP5), the environmental space for the southern cassowary is predicted to reduce over time around its central habitat in the Atherton Tablelands. The maps for the southern cassowary can be compared with [20] for reference.

[INSERT FIGURE 4 HERE]

Figure 4 *This habitat suitability distribution is for the Southern Cassowary (Casuarius casuarius) and presents its historical suitability projection historically, in 2030 and in 2090. The graph below represents the total amount of habitat suitability (km^2) available in each time period, green bars correspond to the maps presented (historical, 2030 and 2090).*

Species-level data summary

The dataset includes suitability maps for species under different climate scenarios and time periods using an ensemble average approach. Through the process of ensemble averaging, the minimum and maximum suitability maps were also produced. These maps can be compared to understand the bounds of how climate change may generally impact habitat suitability in the future. The importance of incorporating multiple GCM projections can be seen by the variation among the minimum, mean, and maximum suitability maps (Figure 5). For the common wallaroo (*Macropus robustus*), the differences between the minimum, mean, and maximum suitability maps are most apparent under worsening climate scenarios. Areas across the southern parts of Australia remain suitable across all three suitability maps, compared to areas in the central and northern parts of their range becoming progressively less suitable. These trends are consistent with other macropod modelling studies that also suggest suitability for the common wallaroo will track south as climate scenarios worsen [48]. The maps for the common wallaroo can also be compared with [20] for reference.

[INSERT FIGURE 5 HERE]

Figure 5 *These habitat suitability distributions are for the common wallaroo (Macropus robustus) for one historical projection, and four future emission scenarios in the year 2090. The habitat suitability distributions of each row of maps represent the minimum, mean, and maximum habitat suitability projections across GCMs. The line graphs represent the total habitat suitability (km^2) for four future emission scenarios over time. The uncertainty band represents the minimum and maximum amount of habitat suitability across GCMs.*

Spatial changes over time

Taking this a step further, geospatial calculations can also be applied to determine the differences between years or climate scenarios. This can be conducted to identify areas of refugia (Equation 3), or the location and magnitude of change between different time periods (Equation 2). To calculate spatial locations of refugia, historical and future suitability maps can be multiplied together to accentuate areas in space that are suitable in both time periods. To calculate spatial changes in habitat suitability through time, historical suitability maps can be subtracted from future suitability maps to spatially accentuate locations that have changed in habitat suitability (i.e., improved in suitability or declined in suitability) across time periods. Using the snow gum (*Eucalyptus pauciflora*) as an example, we find refugia in the alpine region of Australia is predicted to decline for the snow gum under worsening climate scenarios, with declines being most severe in the year 2090 (Figure 6, top). Across all climate scenarios habitat suitability is declining from all areas of the snow gum's range, and we did not identify areas of increases (Figure 6, bottom).

[INSERT FIGURE 6 HERE]

Figure 6 *Refugia and habitat suitability change maps for snow gum (Eucalyptus pauciflora). The Top Panel present climate change refugia for four future emission scenarios in the years 2050 and 2950. Dark green on the refugia maps represent areas that have high predictive suitability historically as well in future time periods. The Bottom Panel present changes in habitat suitability for four future emission scenarios in the years 2050 and 2090. Orange areas indicate placed that decrease in suitability compared to the previous time period, and green areas indicate areas that improve in suitability. White areas indicate no change in suitability.*

Changes in quality-weighted habitat area

The dataset also includes a tabular summary of quality-weighted habitat area in km^2 for each species under different climate scenarios and time periods (Equation 1 and Equation 2). The quality-weighted habitat area values can be analysed and plotted to understand how climate change may impact habitat area for single species or groups of species in the future (Figure 7). When this data is summarised across all species, we can show that in 2030 the distribution of change in habitat area are similar across the four climate scenarios. However, in 2090 the distribution of change in habitat area follows a different pattern across climate scenarios with progressively more species losing progressively more habitat area as climate change worsens (Figure 7).

[INSERT FIGURE 7 HERE]

Figure 7 *Histogram of the number of species and their relative change in quality weighted habitat area between 1990 and each future time period (2030, 2050, 2070, 2090).*

Discussion

Spatial data on the suitability of areas for species is an important input to guide conservation planning, policy and management. The objective of this paper was to develop habitat suitability maps for Australian flora and fauna under different climate futures using a MaxEnt approach. This data has been developed in a way that is consistent across species and enables users to analyze how different climate futures may impact the habitat suitability for biodiversity more generally across Australia. This data can also be used for species-level analysis and can be a starting point for additional analyses which utilize either geospatial information or tabular information that could take into consideration additional information like land use, conservation actions or species ecology.

Applications for landscape and species conservation

This spatial and tabular dataset is ideal for users that would like to understand how the habitat suitability of areas for species is predicted to change over time or under different climate scenarios. For example, at the landscape level, these habitat suitability maps can be combined into a general biodiversity layer to evaluate how habitat suitability more generally changes over time (Figure 7) or over space and time (Figures 4, 5 and 6) [49]. This data can then be utilized in applications such as spatial prioritisations using such tools as Zonation [50] or Marxan [51] to guide spatial conservation priorities in Australia [5,19]. Therefore, using this data for subsequent analysis can be useful to inform conservation (e.g., where to establish new protected areas), restoration or monitoring plans in areas which are suitable for biodiversity, or are predicted to lose or gain suitable areas for biodiversity.

At the species level, this dataset can be used to support conservation actions for species of interest (e.g., threatened species, iconic species, endemic species). The tabular data can be used to systematically identify species of interest based on the way climate change is anticipated to impact the species. Or could be used to inform processes such as threatened species listing [52].

Spatial information about species could also be useful to compare the long-term suitability of areas for threatened species under climate change to inform present day decision-making and species management [53,54]. Could be paired with other types of data to assess the impacts of climate change on species [55]. Or could inform boarder scale biodiversity conservation analyses [56].

Applications in sustainability and natural capital accounting

Biodiversity forms a foundation of broader sustainability ideals, therefore, to measure progress towards sustainability, conservation or corporate goals spatial data on biodiversity can serve as an important input information to the creation of metrics [57,58]. Biodiversity indicators like the species richness, or more complex indicators like the Species Threat Abatement and Restoration metric (STAR) [59] or the biodiversity intactness index (BII) [60] all draw from species layers as input data. Feeding the habitat suitability maps generated in this study into biodiversity layers and into broader sustainability models or assessments can improve the consideration of biodiversity against other environmental or social values. This may include initiatives such as land use planning, or land use change modelling [61,62].

Additionally, as many businesses are transitioning towards 'nature positive' the use of biodiversity to monitor business impacts and progress towards nature positive is necessary. The habitat suitability maps generated in this study can be used to represent key species or biodiversity within natural capital within frameworks such as the System of Environmental-Economic Accounts (SEEA) framework [63], within sustainability assessments such as 'foot printing' to enhance the biodiversity input data [64–66], or within nature-related impact or dependency assessments which inform frameworks like the Taskforce on Nature-Related Financial Disclosures (TNFD) [67].

Limitations and caveats with the data

When using and interpreting the data contained in this dataset it is important to consider the following limitations and considerations. This dataset presents the habitat suitability of areas for species under different climate scenarios and time periods using a correlative approach. These maps are not distribution maps, rather they present habitat suitability based on climate, soil and landscape characteristics. Due to its 5km^2 spatial resolution, the data is best for understanding broader spatial trends that can be integrated into spatial planning [19], rather than more local management such as identifying specific sites for translocation without additional finer detail [55].These maps have not been thresholded, nor do they consider dispersal [20], land use [68], biophysical capacity [69], or attributes that may be important for species of interest (e.g., NDVI, fire or vegetation structure e.g., [55]. There are a multitude of other methods to model suitability and species distributions that have their own use cases and limitations [70,71].

The occurrence points used for this analysis were those originally used for the CliMAS work, and the ALA data were passed through an additional rigorous cleaning process for terrestrial vertebrates only. This process helped reduce the spatial bias and noise in the occurrence points [23]; however, more broadly there are sampling biases that influence the distribution of occurrence points, such as land tenure. To improve on the models, an integrated pathway to ALA into the modelling procedure would be ideal as this would ensure up-to-date input data. However, this can also come with challenges as occurrence data is required to have the same

temporal resolution to the historical or current climate data (i.e., 1990 in this study). While we did use target background files to reduce spatial biases [22], there may still be limitations of this approach at the taxonomic group level, for example for small ranging species [72]. Taxonomic level grouping may still be too broad to adequately capture those species that are highly range restricted and require very specific micro-climate needs, therefore species-specific level grouping may help to overcome this. Background files that are too broad may adequately capture sampling biases or the true relationship between occurrence points and environmental predictors.

MaxEnt models are also prone to overfit but are also less influenced by collinearity than statistical models, we tried mitigating the impacts of overfitting the MaxEnt models by conducting variable selection. In relation to the variables used, we were primarily guided by past efforts that model the suitability of areas across Australia for many species [40,41], however this approach obviously overlooks some variables that can be import to model suitability. For example, we did not consider variables such as the normalized difference vegetation index (NDVI) [73], land use [74], weather [75], or detailed information about vegetation structure or extreme events like fire [55]. Thus, our recommendation is for the users of this data to consider whether the variables used to model habitat suitability in this study is compatible with the species of interest, or whether additional information is required. This will likely be the case if the user is interested in a more fine-scale application of the data, for example at the single species or local level, as this data is best suited for macro-level analyses and applications.

Finally, there is much contention around the best way to assess model performance of MaxEnt models beyond just the AUC, to approaches like the True Skill Statistic value (TSS), the kappa score and the Boyce Index [45,46,76,77]. We present the AUC and the Boyce Index and do not consider the thresholds for these indexes prior to creating the habitat suitability projections, therefore the user can assess the model performance for their species on interest when interpreting the data.

Conclusion

To spatially target conservation actions, spatial information about the location and suitability of areas for species is needed. This study provides a comprehensive dataset of predicted habitat suitability under 4 climate futures, while also incorporating the uncertainty across GCMs. We are providing a spatial and tabular data product at the Australian scale and at 5km^2 resolution that can be used to inform research and decision making at local, regional and national scales. This data can be applied within strategic conservation planning approaches and can be used to identify important areas for species consecration [6]. Spatial information about current and future suitable areas for species is a key component of conservation planning, particularly as the impact of climate change on species and biodiversity is uncertain.

Data availability

All spatial and tabular data are freely accessible in the companion GigaDB repository [78].

List of abbreviations

ALA	Atlas of Living Australia

AUC	Area Under the Curve
BCC-CSM2-MR	A high-resolution version of the Beijing Climate Centre Climate System Model
BI	Boyce Index
BII	Biodiversity Intactness Index
CanESM5	The Canadian Earth System Model version 5
CliMAS	CliMAS was a suite of tools providing interactive maps and generating regional reports to examine the future of species distributions and biodiversity
CMIP3	Coupled Model Intercomparison Project 3
CMIP6	Coupled Model Intercomparison Project 6
CNRM-CM6-1	Climate model developed by the National Centre for Meteorological Research, Météo-France (CNRM) of second generation as developed by the CNRM and the Centre Européen de Recherche et de Formation Avancée en Calcul Scientifique (CERFACS) modelling group.
CNRM-ESM2-1	Earth system model developed by the National Centre for Meteorological Research, Météo-France (CNRM) of second generation as developed by the CNRM and the Centre Européen de Recherche et de Formation Avancée en Calcul Scientifique (CERFACS) modelling group.
CSIRO	Commonwealth Scientific and Industrial Research Organisation
csv	Comma-separated values file
GCM	General Circulation Models
GFDL-ESM4	Geophysical Fluid Dynamics Laboratory Earth System Model 4
IBRA	Interim Biogeographic Regionalisation for Australia
IPCC	Intergovernmental Panel on Climate Change
IPSL-CM6A-LR	Institut Pierre-Simon Laplace climate model
km	Kilometre
lzw compression	Lempel–Ziv–Welch compression
MaxEnt	MaxEnt software for modeling species niches and distributions by applying a machine-learning technique called maximum entropy modeling
MIROC-ES2L	Model for Interdisciplinary Research on Climate, Earth System version 2 for Long-term simulations
MIROC6	Model for Interdisciplinary Research on Climate 6
MRI-ESM2-0	The Meteorological Research Institute Earth System Model
NDVI	Normalized Difference Vegetation Index
P/E plot	Prediction/Expected plot
qwHA	Quality-weighted Habitat Area in (in km2)
RCP	Representative Concentration Pathway
ROC	Area Under the Receiver Operating Curve
SEEA	System of Environmental-Economic Accounts framework
SLURM	Simple Linux Utility for Resource Management

SSP	Shared Socioeconomic Pathway
STAR	Species Threat Abatement and Restoration metric
tif	Tag image file format
TNFD	Taskforce on Nature-Related Financial Disclosures
TSS	True Skill Statistic value
WorldClim	Maps, graphs, tables, and data of the global climate.

Funding

This work was made possible by generous philanthropic support for Climateworks Centre's Land Use Futures program which supported BB and CA. CA was also supported by an Alfred Deakin Postdoctoral Research Fellowship 2023-2025. DS was partly funded by the 2021-2024 ARC Linkage Grant Innovation in agricultural sector Greenhouse Gas abatement in New South Wales, led by Prof Jeff Connor.

Acknowledgements

This work was made possible by generous philanthropic support for Climateworks Centre's Land Use Futures program which supported BB and CA. CA was also supported by an Alfred Deakin Postdoctoral Research Fellowship 2023-2025. DS was partly funded by the 2021-2024 ARC Linkage Grant Innovation in agricultural sector Greenhouse Gas abatement in New South Wales, led by Prof Jeff Connor. The authors would also like to acknowledge those originally involved with the CliMAS project.

Conflict of interest

The authors declare no conflicts of interest.

References

1. Bryan BA, Nolan M, Harwood TD, Connor JD, Navarro-Garcia J, King D, et al.. Supply of carbon sequestration and biodiversity services from Australia’s agricultural land under global change. *Global Environmental Change*. 2014; doi: 10.1016/j.gloenvcha.2014.06.013.

2. Leclère D, Obersteiner M, Barrett M, Butchart SHM, Chaudhary A, De Palma A, et al.. Bending the curve of terrestrial biodiversity needs an integrated strategy. *Nature*. 2020; doi: 10.1038/s41586-020-2705-y.

3. Hanson JO, Schuster R, Strimas-Mackey M, Bennett JR. Optimality in prioritizing conservation projects. *Methods Ecol Evol*. 2019; doi: 10.1111/2041-210X.13264.

4. Summers DM, Bryan BA, Crossman ND, Meyer WS. Species vulnerability to climate change: Impacts on spatial conservation priorities and species representation. *Glob Chang Biol*. 2012; doi: 10.1111/j.1365-2486.2012.02700.x.

5. Summers DM, Bryan BA, Crossman ND, Meyer WS. Species vulnerability to climate change: Impacts on spatial conservation priorities and species representation. *Glob Chang Biol*. 2012; doi: 10.1111/j.1365-2486.2012.02700.x.

6. Tulloch VJD, Tulloch AIT, Visconti P, Halpern BS, Watson JEM, Evans MC, et al.. Why do We map threats? Linking threat mapping with actions to make better conservation decisions. *Front Ecol Environ*. 2015; doi: 10.1890/140022.

7. Coleman S. Australia state of the environment 2016: built environment. independent report to the Australian Government Minister for the Environment and Energy. Canberra; 2016.

8. Chapman AD. Numbers of Living Species in Australia and the World. Toowoomba, Australia: Biodiversity Information Services; ISBN: 978 0 642 56861 8

9. Australian Government Department of Agriculture and the Environment: Species Profile and Threats Database (SPRAT). http://www.environment.gov.au/cgi-bin/sprat/public/sprat.pl (2021). Accessed 2021 Aug 18.

10. Woinarski J, Braby MF, Burbidge AA, Coates D, Garnett ST, Fensham RJ, et al.. Reading the black book: The number, timing, distribution and causes of listed extinctions in Australia. *Biol Conserv*. Elsevier; 2019; doi: 10.1016/j.biocon.2019.108261.

11. Bergstrom DM, Wienecke BC, van den Hoff J, Hughes L, Lindenmayer DB, Ainsworth TD, et al.. Combating ecosystem collapse from the tropics to the Antarctic. *Glob Chang Biol*. 2021; doi: 10.1111/gcb.15539.

12. Kearney SG, Cawardine J, Reside AE, Fisher DO, Maron M, Doherty TS, et al.. The threats to Australia’s imperilled species and implications for a national conservation response. *Pacific Conservation Biology*. 2018; doi: 10.1071/PC18024.

13. Garnett S, Hayward-Brown BK, Kopf RK, Woinarski JCZ, Cameron KA, Chapple DG, et al.. Australia’s most imperilled vertebrates. *Biol Conserv*. 2022; doi: 10.1016/j.biocon.2022.109561.

14. Reside AE, Welbergen JA, Phillips BL, Wardell-Johnson GW, Keppel G, Ferrier S, et al.. Characteristics of climate change refugia for Australian biodiversity. *Austral Ecol*. 2014; doi: 10.1111/aec.12146.

15. Phillips, Aneja VP, Kang D, Arya SP. Maximum entropy modeling of species geographic distributions. *Ecol Modell*. 2006; doi: 10.1016/j.ecolmodel.2005.03.026.

16. Elith J, H. Graham C, P. Anderson R, Dudík M, Ferrier S, Guisan A, et al.. Novel methods improve prediction of species’ distributions from occurrence data. *Ecography*. 2006; doi: 10.1111/j.2006.0906-7590.04596.x.

17. Meehl GA, Covey C, Delworth T, Latif M, Mcavaney B, Mitchell JFB, et al.. The WCRP CMIP3 Multi-model Dataset: A New Era in Climate Change Research. 2007.

18. Ward M, Tulloch A, Stewart R, Possingham HP, Legge S, Gallagher R V., et al.. Restoring habitat for fire-impacted species’ across degraded Australian landscapes. *Environmental Research Letters*. Institute of Physics; 2022; doi: 10.1088/1748-9326/ac83da.

19. Maxwell SL, Reside A, Trezise J, McAlpine CA, Watson JE. Retention and restoration priorities for climate adaptation in a multi-use landscape. *Glob Ecol Conserv*.; 2019; doi: 10.1016/j.gecco.2019.e00649.

20. Graham EM, Reside AE, Atkinson I, Baird D, Hodgson L, James CS, et al.. Climate change and biodiversity in Australia: a systematic modelling approach to nationwide species distributions. *Australasian Journal of Environmental Management*. 2019; doi: 10.1080/14486563.2019.1599742.

21. Atlas of Living Australia.: Species occurance records download. https://support.ala.org.au/ (2012).

22. Barber RA, Ball SG, Morris RKA, Gilbert F. Target-group backgrounds prove effective at correcting sampling bias in Maxent models. *Divers Distrib*. 2022; doi: 10.1111/ddi.13442.

23. Phillips, Dudík M, Elith J, Graham CH, Lehmann A, Leathwick J, et al.. Sample selection bias and presence-only distribution models: Implications for background and pseudo-absence data. *Ecological Applications*. 2009; doi: 10.1890/07-2153.1.

24. WorldClim: Bioclimatic variables. www.worldclim.org.

25. Wu T, Yu R, Lu Y, Jie W, Fang Y, Zhang J, et al.. BCC-CSM2-HR: A high-resolution version of the Beijing Climate Center Climate System Model. *Geosci Model Dev*. 2021; doi: 10.5194/gmd-14-2977-2021.

26. Voldoire A, Saint-Martin D, Sénési S, Decharme B, Alias A, Chevallier M, et al.. Evaluation of CMIP6 DECK Experiments With CNRM-CM6-1. *J Adv Model Earth Syst*. 2019; doi: 10.1029/2019MS001683.

27. Séférian R, Nabat P, Michou M, Saint-Martin D, Voldoire A, Colin J, et al.. Evaluation of CNRM Earth System Model, CNRM-ESM2-1: Role of Earth System Processes in Present-Day and Future Climate. *J Adv Model Earth Syst*. 2019; doi: 10.1029/2019MS001791.

28. Swart NC, Cole JNS, Kharin V V., Lazare M, Scinocca JF, Gillett NP, et al.. The Canadian Earth System Model version 5 (CanESM5.0.3). *Geosci Model Dev*. 2019; doi: 10.5194/gmd-12-4823-2019.

29. Krasting JP, John JG, Blanton C, McHugh C, Nikonov S, Radhakrishnan A, et al.. NOAA-GFDL GFDL-ESM4 model output prepared for CMIP6 CMIP. 2018 Sep. Earth System Grid Federation. https://doi.org/10.22033/ESGF/CMIP6.1407

30. Boucher O, Servonnat J, Albright AL, Aumont O, Balkanski Y, Bastrikov V, et al.. Presentation and Evaluation of the IPSL-CM6A-LR Climate Model. *J Adv Model Earth Syst*. 2020; doi: 10.1029/2019MS002010.

31. Hajima T, Watanabe M, Yamamoto A, Tatebe H, Noguchi MA, Abe M, et al.. Development of the MIROC-ES2L Earth system model and the evaluation of biogeochemical processes and feedbacks. *Geosci Model Dev*. 2020; doi: 10.5194/gmd-13-2197-2020.

32. Tatebe H, Ogura T, Nitta T, Komuro Y, Ogochi K, Takemura T, et al.. Description and basic evaluation of simulated mean state, internal variability, and climate sensitivity in MIROC6. *Geoscientific Model Development Discussions*. 2018; doi: 10.5194/gmd-2018-155.

33. Yukimoto S, Kawai H, Koshiro T, Oshima N, Yoshida K, Urakawa S, et al.. The meteorological research institute Earth system model version 2.0, MRI-ESM2.0: Description and basic evaluation of the physical component. *Journal of the Meteorological Society of Japan*. Meteorological Society of Japan; 2019; doi: 10.2151/jmsj.2019-051.

34. Riahi K, van Vuuren DP, Kriegler E, Edmonds J, O'Neill BC, Fujimori S, et al.. The Shared Socioeconomic Pathways and their energy, land use, and greenhouse gas emissions implications: An overview. *Global Environmental Change*. 2017; doi: 10.1016/j.gloenvcha.2016.05.009.

35. CSIRO: Soil and Landscape Grid of Australia. https://www.csiro.au/en/research/natural-environment/land/soil-and-landscape-grid-of-australia.

36. Department of Climate Change E the E and WAG: Interim Biogeographic Regionalisation for Australia (IBRA). https://www.dcceew.gov.au/environment/land/nrs/science/ibra.

37. Archibald C. CarlaBirdy/MaxEnt-habitat-models: MaxEnt-habitat-models (v1.0.2). Zenodo;

38. Low BW, Zeng Y, Tan HH, Yeo DCJ. Predictor complexity and feature selection affect Maxent model transferability: Evidence from global freshwater invasive species. *Divers Distrib*. 2021; doi: 10.1111/ddi.13211.

39. Feng X, Park DS, Liang Y, Pandey R, Papeş M. Collinearity in ecological niche modeling: Confusions and challenges. *Ecol Evol*. John Wiley and Sons Ltd; 2019; doi: 10.1002/ece3.5555.

40. Gallagher R V., Allen S, Wright IJ. Safety margins and adaptive capacity of vegetation to climate change. *Sci Rep*. 2019; doi: 10.1038/s41598-019-44483-x.

41. Butt N, Pollock LJ, Mcalpine CA. Eucalypts face increasing climate stress. *Ecol Evol*. 2013; doi: 10.1002/ece3.873.

42. Hageer Y, Esperón-Rodríguez M, Baumgartner JB, Beaumont LJ. Climate, soil or both? Which variables are better predictors of the distributions of Australian shrub species? *PeerJ*. 2017; doi: 10.7717/peerj.3446.
43. Merow C, Smith MJ, Silander JA. A practical guide to MaxEnt for modeling species' distributions: What it does, and why inputs and settings matter. *Ecography*. 2013; doi: 10.1111/j.1600-0587.2013.07872.x.
44. Boyce MS, Vernier PR, Nielsen SE, Schmiegelow FKA. Evaluating resource selection functions. *Ecol Modell*. 2002; doi: 10.1016/S0304-3800(02)00200-4.
45. Hirzel AH, Le Lay G, Helfer V, Randin C, Guisan A. Evaluating the ability of habitat suitability models to predict species presences. *Ecol Modell*. 2006; doi: 10.1016/j.ecolmodel.2006.05.017.
46. Jiménez L, Soberón J. Leaving the area under the receiving operating characteristic curve behind: An evaluation method for species distribution modelling applications based on presence-only data. *Methods Ecol Evol*. 2020; doi: 10.1111/2041-210X.13479.
47. Wilkinson MD, Dumontier M, Aalbersberg IjJ, Appleton G, Axton M, Baak A, et al.. Comment: The FAIR Guiding Principles for scientific data management and stewardship. *Sci Data*. 2016; doi: 10.1038/sdata.2016.18.
48. Ritchie EG, Bolitho EE. Australia's savanna herbivores: Bioclimatic distributions and an assessment of the potential impact of regional climate change. *Physiological and Biochemical Zoology*. 2008; doi: 10.1086/588171.
49. Hama AA, Khwarahm NR. Predictive mapping of two endemic oak tree species under climate change scenarios in a semiarid region: Range overlap and implications for conservation. *Ecol Inform*. 2023; doi: 10.1016/j.ecoinf.2022.101930.
50. Minin E, Veach V, Lehtomäki J, Pouzols FM, Moilanen A. A quick introduction to Zonation. Version 1 (for Zv4). User Manual. 1:1–302014;
51. Watts ME, Ball IR, Stewart RS, Klein CJ, Wilson K, Steinback C, et al.. Marxan with Zones: Software for optimal conservation based land- and sea-use zoning. *Environmental Modelling and Software*. 2009; doi: 10.1016/j.envsoft.2009.06.005.
52. IUCN: The IUCN Red List of Threatened Species (IUCN). p. Downloaded on 19 Feb 2022. http://www.iucnredlist.org (2022).
53. Harley D. Seven urgent actions to prevent the extinction of the critically endangered Leadbeater's possum (Gymnobelideus leadbeateri). *Pacific Conservation Biology*. 2023; doi: 10.1071/PC22021.
54. Hawke T, Bino G, Kingsford RT, Grant T, Griffiths J, Weeks A, et al.. A national assessment of the conservation status of the platypus. 2020. http://nla.gov.au/nla.obj-3163334619
55. Eyre AC, Briscoe NJ, Harley DKP, Lumsden LF, McComb LB, Lentini PE. Using species distribution models and decision tools to direct surveys and identify potential translocation sites for a critically endangered species. *Divers Distrib*. 2022; doi: 10.1111/ddi.13469.
56. Engert JE, Pressey RL, Adams VM. Threatened fauna protections compromised by agricultural interests in Australia. *Conserv Lett*. 2023; doi: 10.1111/conl.12975.
57. Lamb EG, Bayne E, Holloway G, Schieck J, Boutin S, Herbers J, et al.. Indices for monitoring biodiversity change: Are some more effective than others? *Ecol Indic*. 2009; doi: 10.1016/j.ecolind.2008.06.001.
58. Watermeyer KE, Guillera-Arroita G, Bal P, Burgass MJ, Bland LM, Collen B, et al.. Using decision science to evaluate global biodiversity indices. *Conservation Biology*. 2021; doi: 10.1111/cobi.13574.

59. Mair L, Bennun LA, Brooks TM, Butchart SHM, Bolam FC, Burgess ND, et al.. A metric for spatially explicit contributions to science-based species targets. *Nat Ecol Evol*. 2021; doi: 10.1038/s41559-021-01432-0.

60. Biggs R, Scholes RJ. A biodiversity intactness index. *Nature*. 434:45–92005;

61. Gao L, Bryan BA. Finding pathways to national-scale land-sector sustainability. *Nature*. 2017; doi: 10.1038/nature21694.

62. Connor JD, Bryan BA, Nolan M, Stock F, Gao L, Dunstall S, et al.. Modelling Australian land use competition and ecosystem services with food price feedbacks at high spatial resolution. *Environmental Modelling and Software*. 2015; doi: 10.1016/j.envsoft.2015.03.015.

63. UNEP, UNSD, CBD, NORAD. SEEA Experimental Ecosystem Accounting: Technical Recommendations. 2015. https://seea.un.org/content/technical-recommendations-support-seea-eea

64. Halpern BS, Frazier M, Verstaen J, Rayner PE, Clawson G, Blanchard JL, et al.. The environmental footprint of global food production. *Nat Sustain*. 2022; doi: 10.1038/s41893-022-00965-x.

65. Hoang NT, Taherzadeh O, Ohashi H, Yonekura Y, Nishijima S, Yamabe M, et al.. Mapping potential conflicts between global agriculture and terrestrial conservation. *Proc Natl Acad Sci U S A*. 2023; doi: 10.1073/pnas.2208376120.

66. Irwin A, Geschke A. A consumption-based analysis of extinction risk in Australia. *Conserv Lett*. 2023; doi: 10.1111/conl.12942.

67. TNFD. Recommendations of the Taskforce on Nature-related Financial Disclosures. 2023. https://tnfd.global/

68. Kapitza S, Van Ha P, Kompas T, Golding N, Cadenhead NCR, Bal P, et al.. Assessing biophysical and socio-economic impacts of climate change on regional avian biodiversity. *Sci Rep*. 2021; doi: 10.1038/s41598-021-82474-z.

69. Briscoe NJ, Morris SD, Mathewson PD, Buckley LB, Jusup M, Levy O, et al.. Mechanistic forecasts of species responses to climate change: The promise of biophysical ecology. Glob Chang Biol. 2023 Mar;29(6):1451-1470. doi: 10.1111/gcb.16557.

70. Elith J, Graham CH. Do they? How do they? WHY do they differ? on finding reasons for differing performances of species distribution models. *Ecography*. 2009; doi: 10.1111/j.1600-0587.2008.05505.x.

71. Briscoe NJ, Kearney MR, Taylor CA, Wintle BA. Unpacking the mechanisms captured by a correlative species distribution model to improve predictions of climate refugia. *Glob Chang Biol*. 2016; doi: 10.1111/gcb.13280.

72. Breiner FT, Guisan A, Bergamini A, Nobis MP. Overcoming limitations of modelling rare species by using ensembles of small models. *Methods Ecol Evol*. 2015; doi: 10.1111/2041-210X.12403.

73. Wen L, Saintilan N, Yang X, Hunter S, Mawer D. MODIS NDVI based metrics improve habitat suitability modelling in fragmented patchy floodplains. *Remote Sens Appl*. 2015; doi: 10.1016/j.rsase.2015.08.001.

74. Lentini PE, Wintle BA. Spatial conservation priorities are highly sensitive to choice of biodiversity surrogates and species distribution model type. *Ecography*. 2015; doi: 10.1111/ecog.01252.

75. Reside AE, Vanderwal JJ, Kutt AS, Perkins GC. Weather, Not Climate, Defines Distributions of Vagile Bird Species. *PLoS One*. 2010; doi: 10.1371/journal.pone.0013569.

76. Valavi R, Guillera-Arroita G, Lahoz-Monfort JJ, Elith J. Predictive performance of presence-only species distribution models: a benchmark study with reproducible code. *Ecol Monogr*. 2022; doi: 10.1002/ecm.1486.
77. Allouche O, Tsoar A, Kadmon R. Assessing the accuracy of species distribution models: prevalence, kappa and the true skill statistic (TSS). *Journal of Applied Ecology*. 2006; doi: 10.1111/j.1365-2664.2006.01214.x.
78. Archibald CL; Summers DM; Graham EM; Bryan BA. Supporting data for "Habitat suitability maps for Australian flora and fauna under CMIP6 climate scenarios" GigaScience Database 2023. http://dx.doi.org/10.5524/102491

 Click here to access/download;Table;Table_1.docx

***Table 1** Summary of the bioclimatic, soil and landscape variable selected in the final MaxEnt model.*

Code	Variable Name	Contribution[1]	Importance[2]	Ecological Rationale
Bioclimatic variables				
BIO1	Annual Mean Temperature	8.72	18.21	Influences thermal tolerances of species.
BIO5	Max Temperature of Warmest Month	6.33	9.92	Influences upper thermal tolerances of species through extreme temperatures.
BIO6	Min Temperature of Coldest Month	4.30	8.66	Influences lower thermal tolerances of species through extreme temperatures.
BIO12	Annual Precipitation	8.60	10.81	Average annual rainfall which influences water availability.
BIO13	Precipitation of Wettest Month	17.67	7.77	Maximum rainfall in the wettest month which influences maximum water availability.
BIO14	Precipitation of Driest Month	14.93	8.45	Minimum rainfall in the driest month which influences minimum water availability.
BIO15	Precipitation Seasonality	12.13	13.20	Standard deviation of rainfall in the annually which influences the variation in water availability.
Soil and landscape variables				
AWC	Available Water Capacity	0.94	0.68	The amount of water held by the soil for future use.
BDW	Bulk Density (Whole Earth)	0.89	1.17	Soil's ability to function for structural support, water and nutrient and microbial life movement, and soil aeration.
CLY	Clay	1.04	0.95	Promotes water retention and reduces air circulation in soil.
DES	Depth of Soil	2.00	1.29	Defines the root space and volume of soils available.
ECE	Electroconductivity	3.39	5.21	Movement of nutrients within the soil which influences the availability of soil nutrients.
elev	Elevation	2.37	1.57	Elevation influences soil properties and air pressure.
pHc	pH	5.43	4.30	Affects the amount of nutrients that are water soluble in soil.
slope	Slope Relief	1.81	1.00	Influences soil properties and creates varying microclimates.
SLT	Silt	2.63	2.10	Promotes water retention and creates relatively porous soil conditions.
SND	Sand	1.60	1.60	Promotes water drainage and air circulation in soil.
SOC	Organic Carbon	5.17	3.05	Promotes soil structure by providing a food source for micro-organisms.

[1] *Average (mean) percent contribution in the final models for each environmental variable across all species. A measure of the contribution of each variable towards model fit after each iteration of the MaxEnt model.*
[2] *Average (mean) percent importance in the final models for each environmental variable across all species. A measure of the importance of each variable measure depends the resulting decrease in training AUC on the final MaxEnt model.*

Graphical abstract

Click here to access/download;Figure;Graphical-Abstract.png

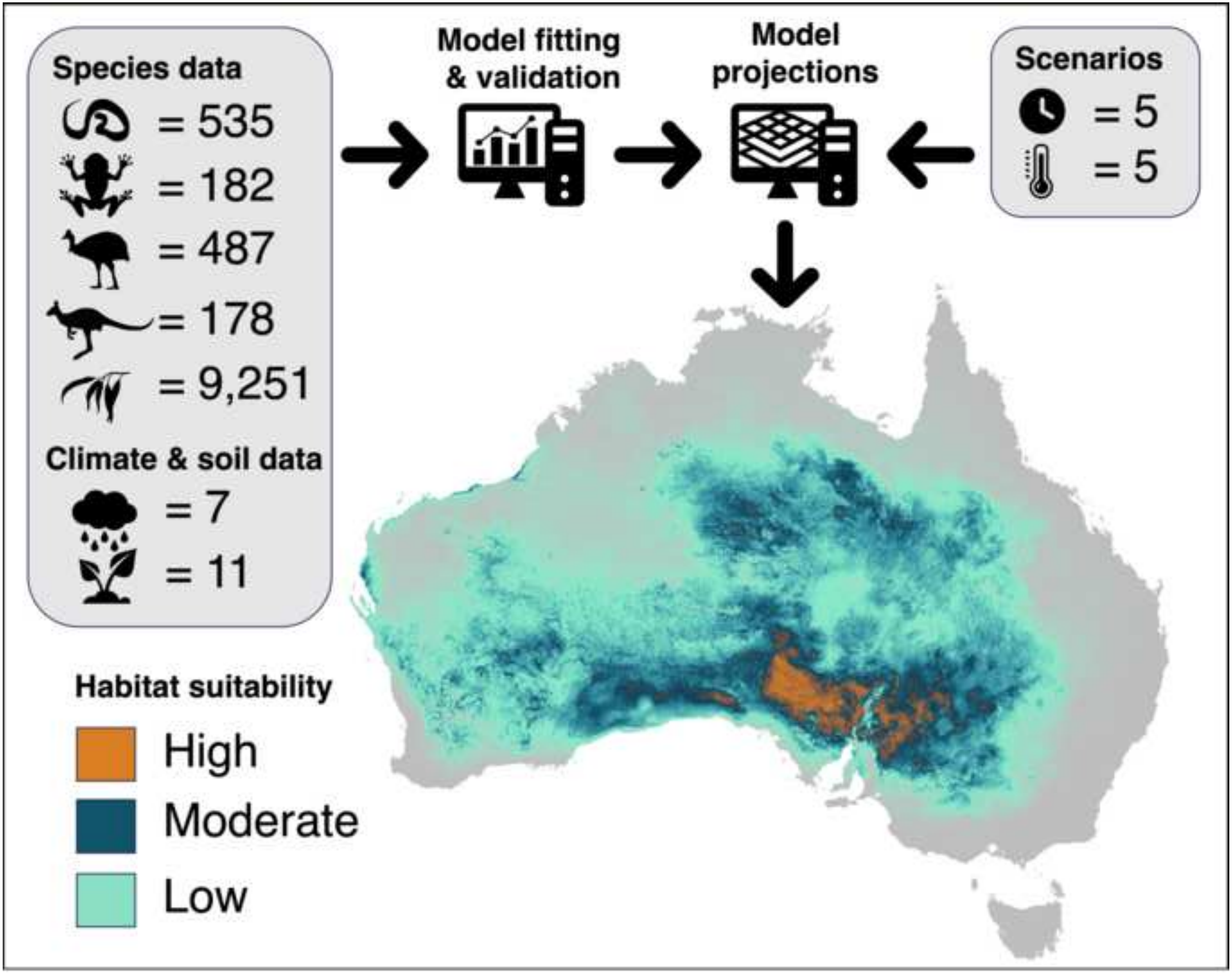

Figure 1: Workflow

Click here to access/download;Figure;Figure_1.png

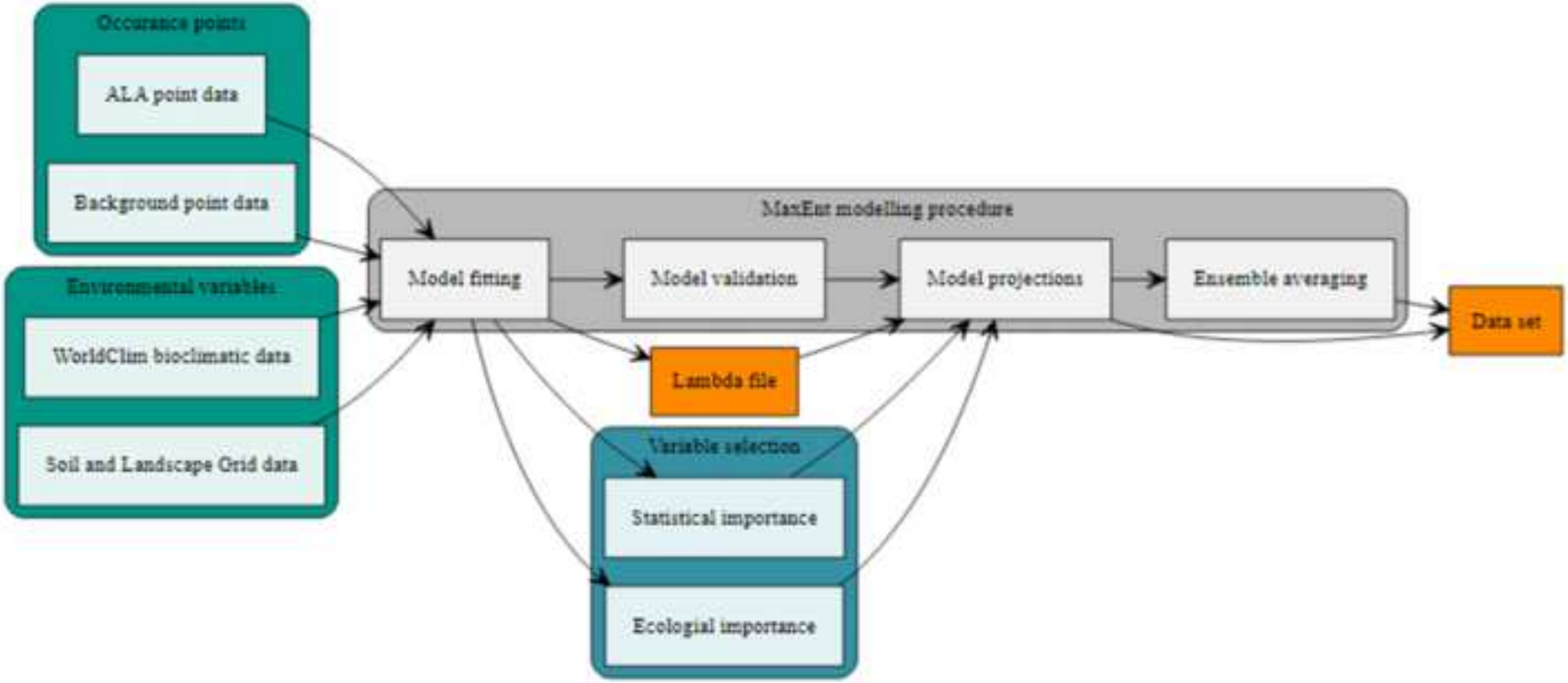

Figure 2: Occurance point histograpm

Click here to access/download;Figure;Figure_2.png

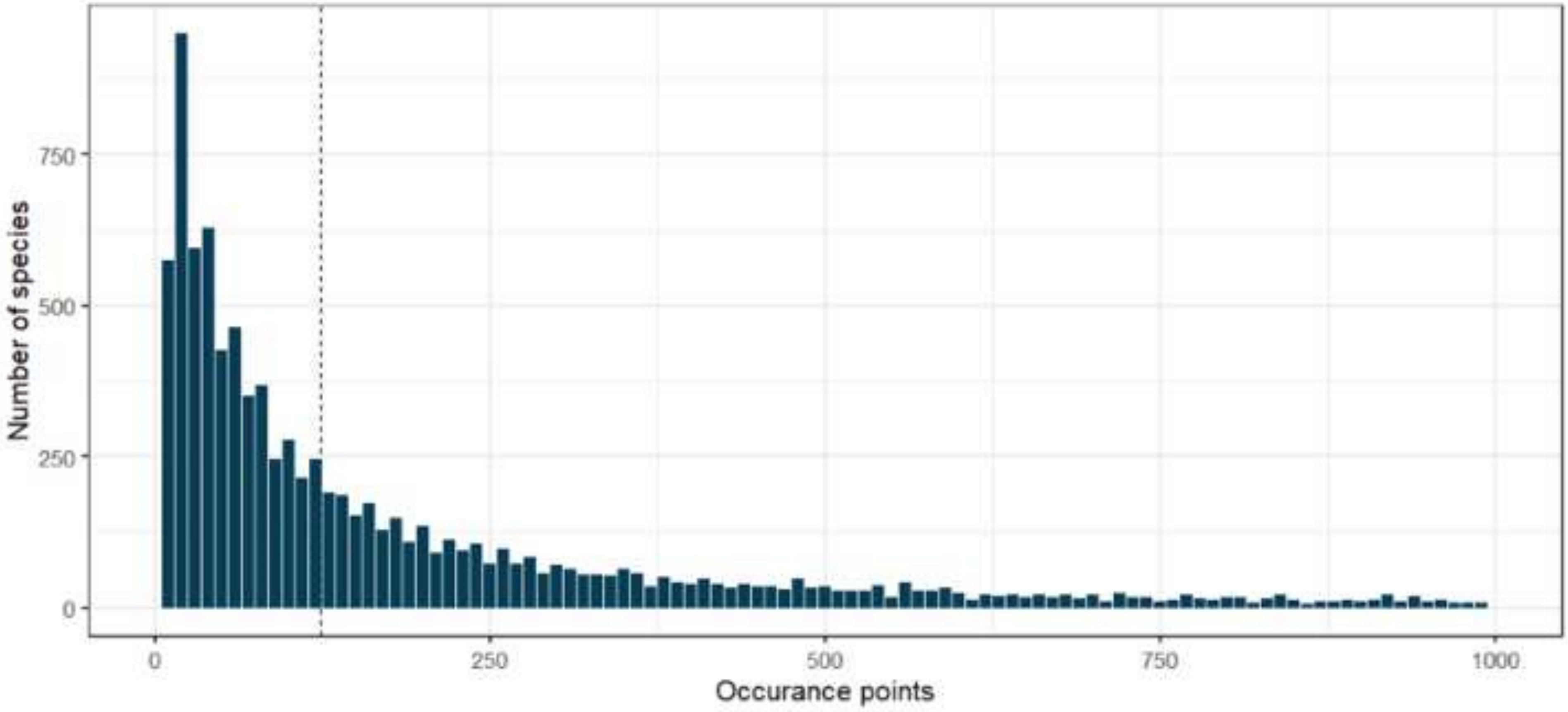

Figure 3: Model validation

Click here to access/download;Figure;Figure_3.png

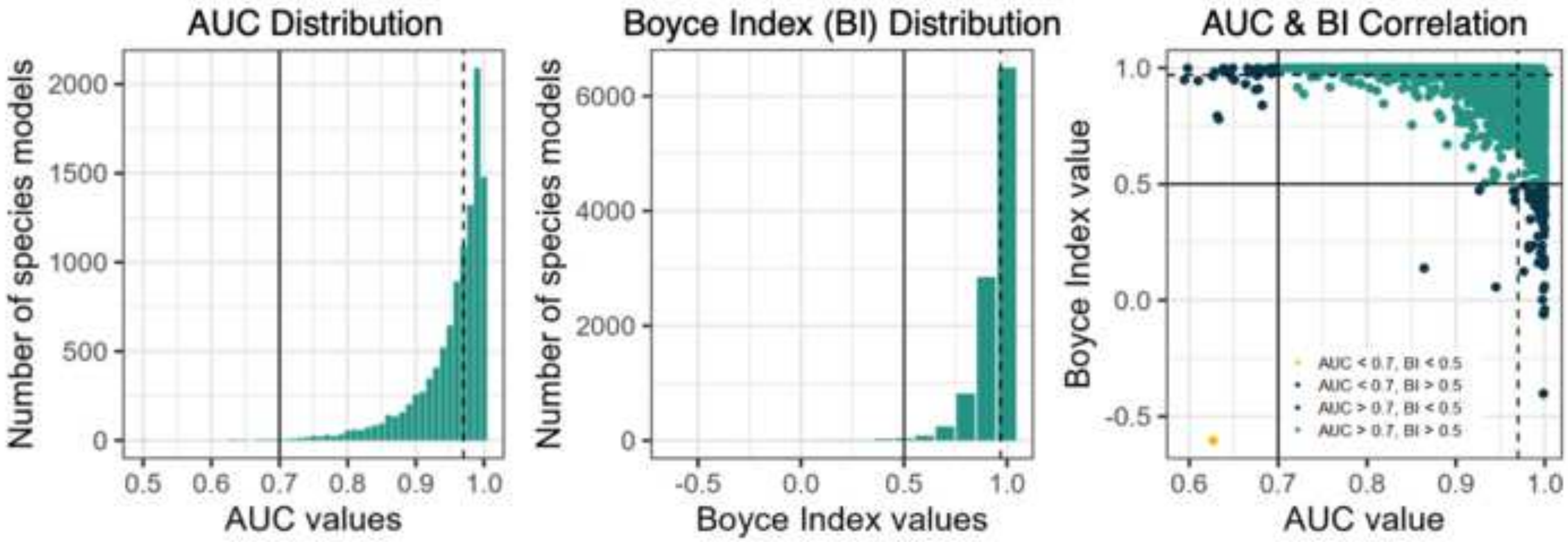

Figure 4: Spatial distribution over time

Click here to access/download;Figure;Figure_4.png

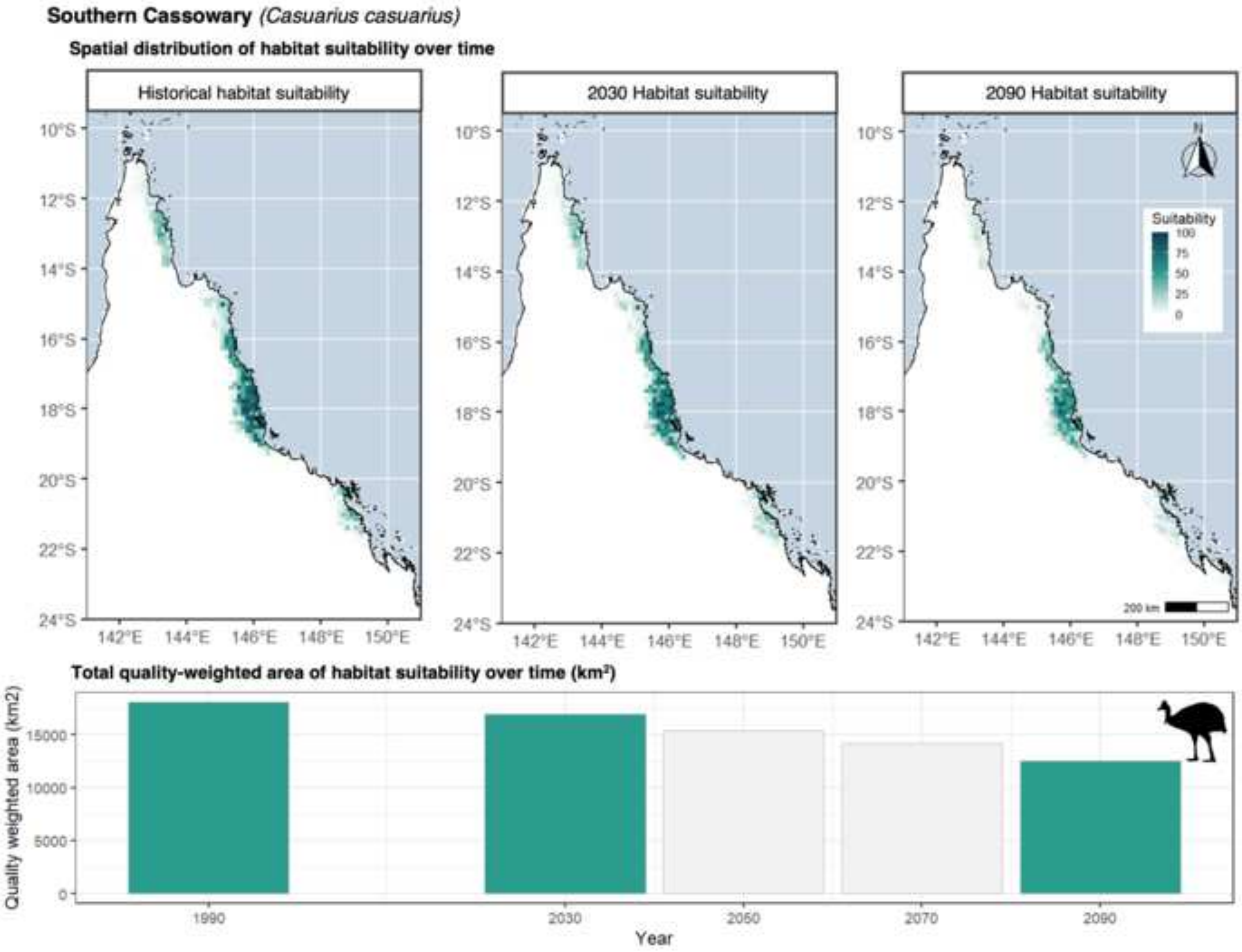

Figure 5: Variance in suitability projections

Click here to access/download;Figure;Figure_5.png

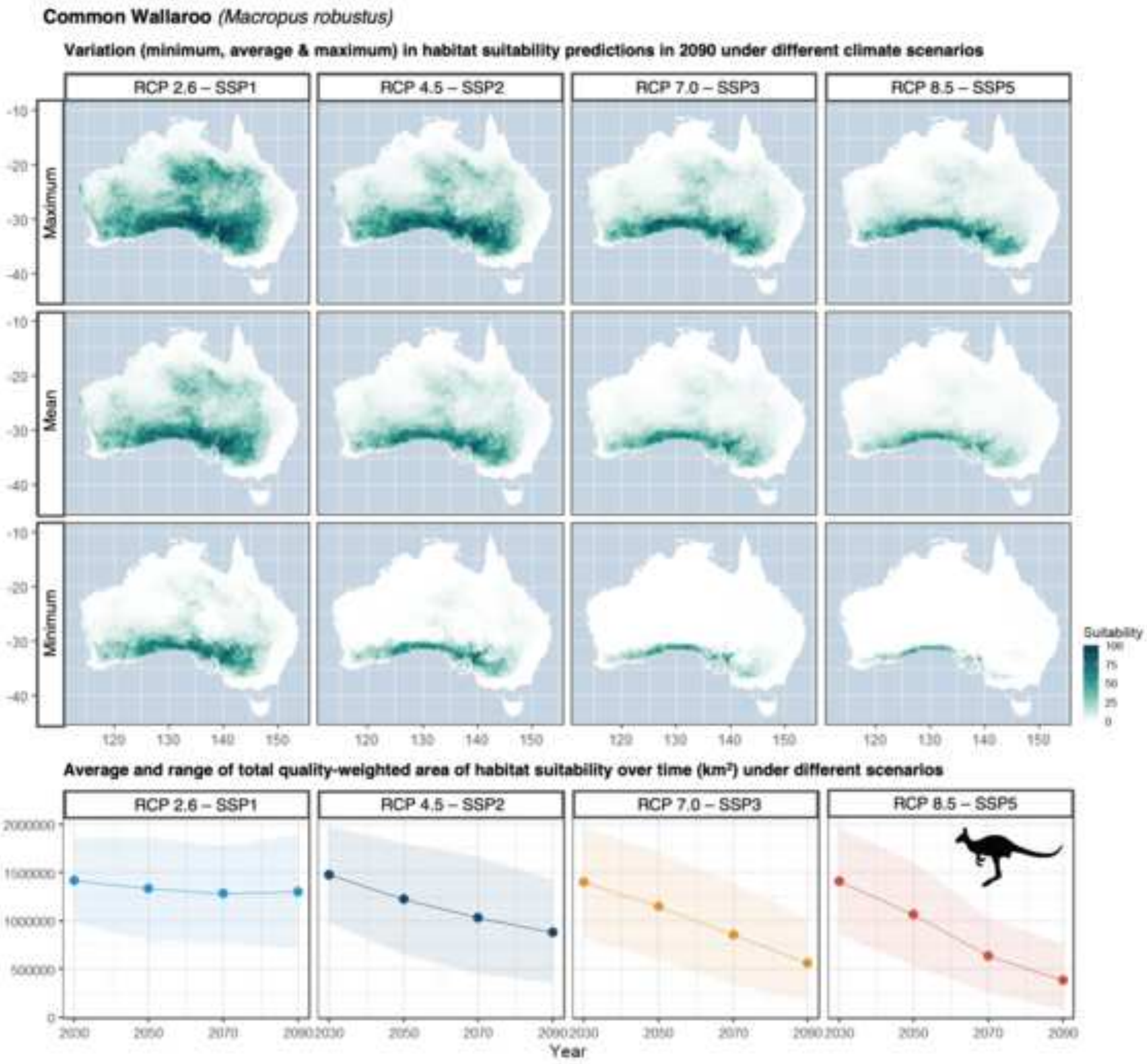

Figure 6: Refugia and change

Click here to access/download;Figure;Figure_6.png

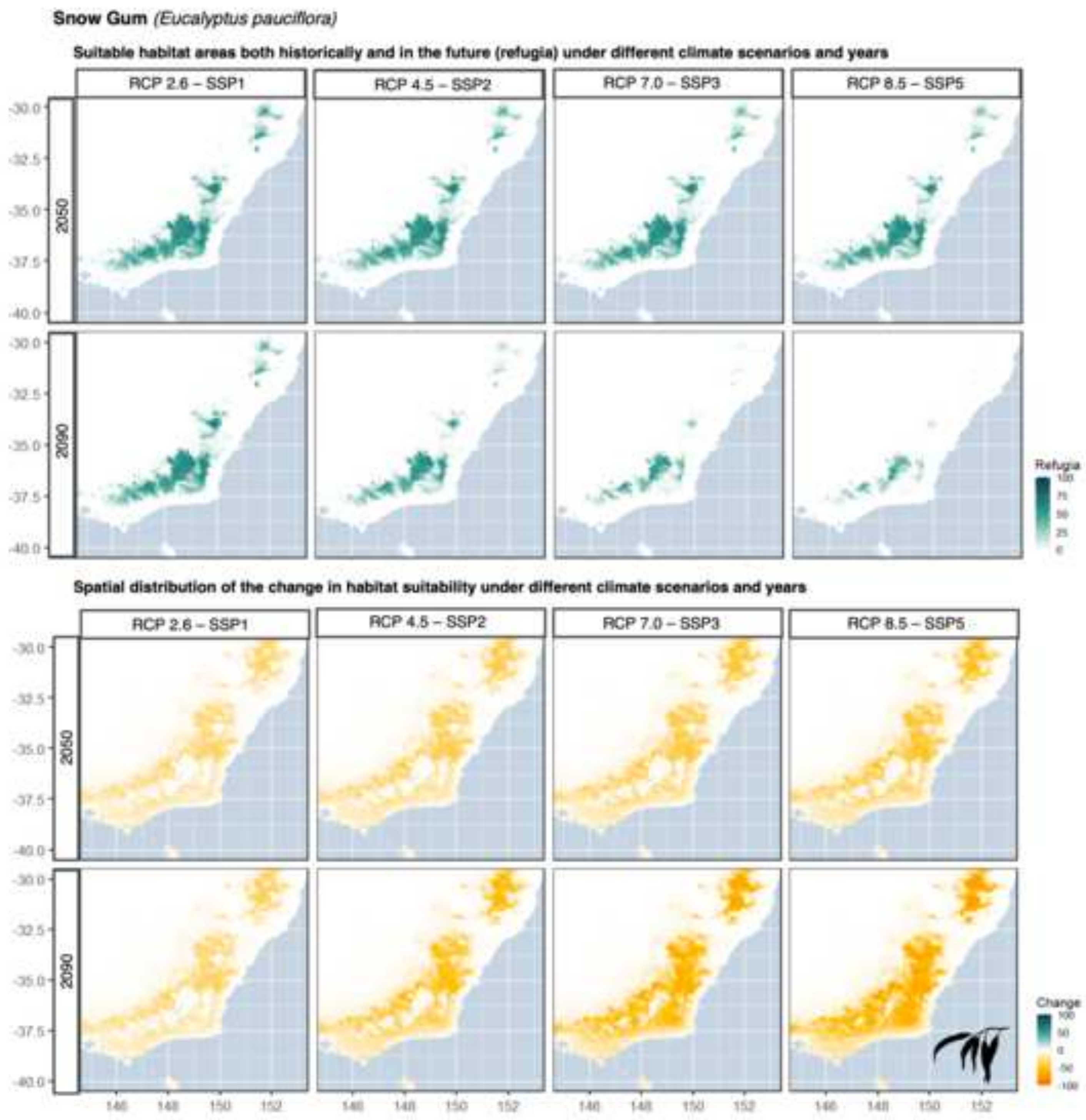

Figure 7: Suitability graphs over time and scenarios

Click here to access/download;Figure;Figure_7.png

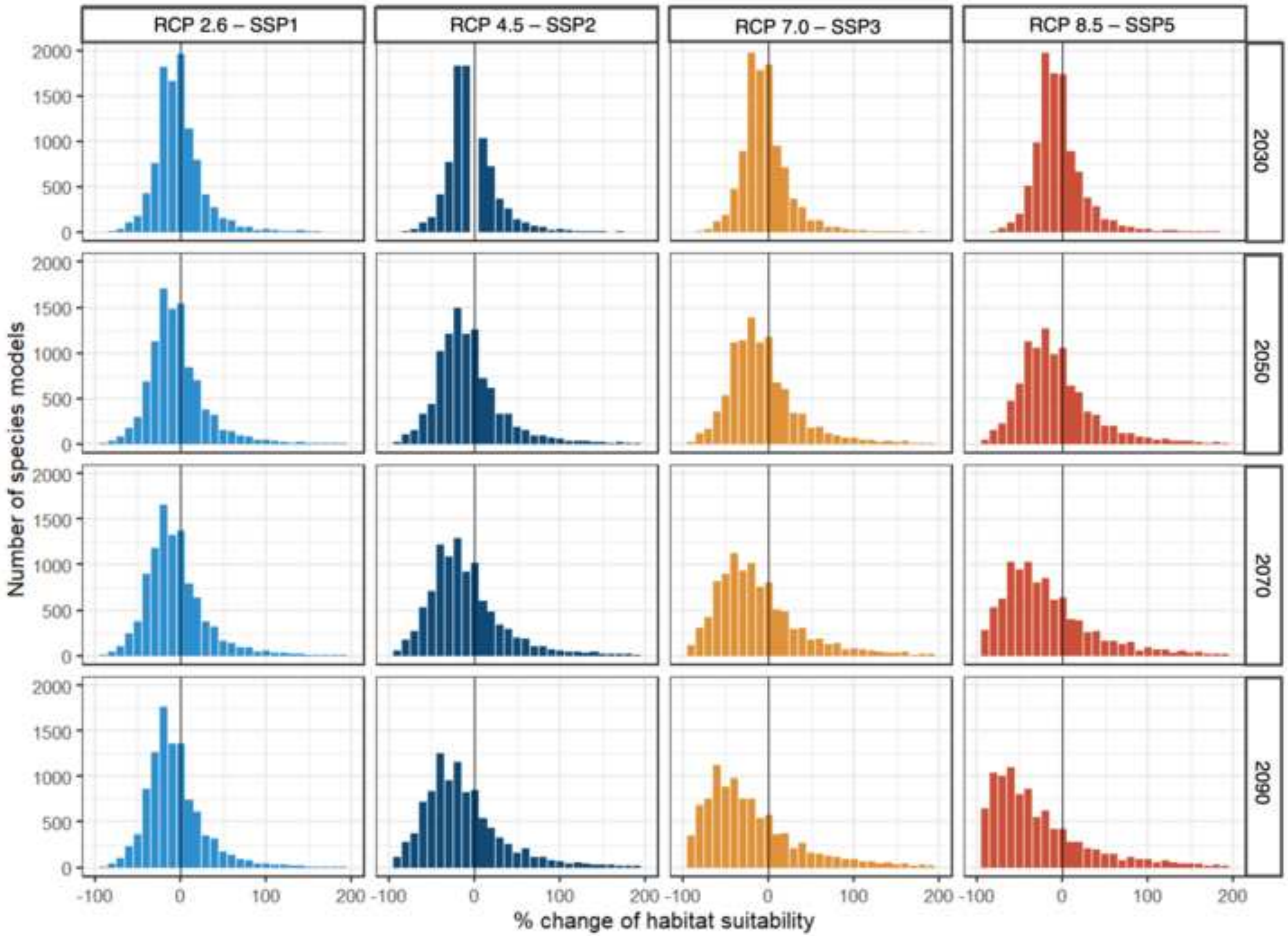

Click here to access/download
Supplementary Material
GIGA-D-23-00183_Supplementary-material_GigaDBUploadForm.xlsx

Personal Cover

Click here to access/download;Personal Cover;GIGA-D-23-00183_Reviewer-report.docx

Associate Editor

Dear Carla,

Your manuscript "Habitat suitability maps for Australian flora and fauna under CMIP6 climate scenarios" (GIGA-D-23-00183) has been assessed by our reviewers. Although it is of interest, we are unable to consider it for publication in its current form. The reviewers have raised a number of points which we believe would improve the manuscript and may allow a revised version to be published in GigaScience.

Their reports, together with any other comments, are below. Please also take a moment to check our website at https://aus01.safelinks.protection.outlook.com/?url=https%3A%2F%2Fwww.editorialmanager.com%2Fgiga%2F&data=05%7C01%7Cc.archibald%40deakin.edu.au%7Cb83f42e4d5064d41996308dbb018b752%7Cd02378ec168846d585401c28b5f470f6%7C0%7C0%7C638297393738149435%7CUnknown%7CTWFpbGZsb3d8eyJWIjoiMC4wLjAwMDAiLCJQIjoiV2luMzIiLCJBTiI6Ik1haWwiLCJXVCI6Mn0%3D%7C3000%7C%7C%7C&sdata=OVNQPHhHe9gvRwMTeo3zMAZJrUvc5C2G5yKxN9YUZlA%3D&reserved=0 for any additional comments that were saved as attachments.

In addition, please register any new software application in the bio.tools and SciCrunch.org databases to receive RRID (Research Resource Identification Initiative ID) and biotoolsID identifiers, and include these in your manuscript. Computational workflows should be registered in workflowhub.eu and the DOIs cited in the relevant places in the manuscript. These will facilitate tracking, reproducibility and re-use of your tool.

If you are able to fully address these points, we would encourage you to submit a revised manuscript to GigaScience. Once you have made the necessary corrections, please submit online at:

https://aus01.safelinks.protection.outlook.com/?url=https%3A%2F%2Fwww.editorialmanager.com%2Fgiga%2F&data=05%7C01%7Cc.archibald%40deakin.edu.au%7Cb83f42e4d5064d41996308dbb018b752%7Cd02378ec168846d585401c28b5f470f6%7C0%7C0%7C638297393738149435%7CUnknown%7CTWFpbGZsb3d8eyJWIjoiMC4wLjAwMDAiLCJQIjoiV2luMzIiLCJBTiI6Ik1haWwiLCJXVCI6Mn0%3D%7C3000%7C%7C%7C&sdata=OVNQPHhHe9gvRwMTeo3zMAZJrUvc5C2G5yKxN9YUZlA%3D&reserved=0

If you have forgotten your username or password please use the "Send Login Details" link to get your login information. For security reasons, your password will be reset.

Please include a point-by-point within the 'Response to Reviewers' box in the submission system. Please ensure you describe additional experiments that were carried out and include a detailed rebuttal of any criticisms or requested revisions that you disagreed with. Please also ensure that your revised manuscript conforms to the journal style, which can be found in the Instructions for Authors on the journal homepage. If the data and code has been modified in the revision process please be sure to update the public versions of this too.

The due date for submitting the revised version of your article is 06 Dec 2023.

I look forward to receiving your revised manuscript soon.

Best wishes,

Hongling Zhou
GigaScience
https://aus01.safelinks.protection.outlook.com/?url=http%3A%2F%2Fwww.gigasciencejournal.com%2F&data=05%7C01%7Cc.archibald%40deakin.edu.au%7Cb83f42e4d5064d41996308dbb018b752%7Cd02378ec168846d585401c28b5f470f6%7C0%7C0%7C638297393738149435%7CUnknown%7CTWFpbGZsb3d8eyJWIjoiMC4wLjAwMDAiLCJQIjoiV2luMzIiLCJBTiI6Ik1haWwiLCJXVCI6Mn0%3D%7C3000%7C%7C%7C&sdata=nq1%2BnH5afs0LilMaNqUnsiJLEIhZr4%2BlytT9lTJoYfQ%3D&reserved=0

Dear Hongling Zhou,

Thank you for these three constructive reviews and your feedback on this manuscript, "*Habitat suitability maps for Australian flora and fauna under CMIP6 climate scenarios*" (GIGA-D-23-00183). We note the 12 key points of feedback raised by the reviewers, and have addressed them line-by-line.

We have added additional information about the occurrence point data used, and have addressed the concerns about sample bias by describing the use of our target group background points in more detail. We have acknowledged the comments on model validation raised by reviewer 1 and reviewer two and have discussed the limitations of using AUC to evaluate model performance [*as well as added one additional indicator of model performance*]. Reviewer 2 also emphasised to value of adding additional information about the practical uses of the data, which we have addressed in the discussion by adding two new sections on "*Applications for landscape and species conservation*", and on "*Applications in sustainability and natural capital accounting*". Overall, we have also greatly expanded the limitations and caveats section to ensure that these elements are discussed in as much detail as possible.

We are confident that these changes to the manuscript make this study a much more robust and useful contribution to the literature, and we look forward to your response. Please find detailed line-by-line changes below.

Sincerely,
Carla Archibald

Reviewer reports:

Reviewer #1:

Title: Habitat suitability maps for Australian flora and fauna under CMIP6 climate scenarios
MS#: GIGA-D-23-00183 Article type: Data Note

The maximum entropy technique was used by the authors to study the potential geographic distribution of thousands of terrestrial vertebrate and plant species and estimated the potential effects of climate change. This data note contains a wealth of informative material. Our understanding of the complex interactions between Australian species and their surroundings is improved by this painstaking and analytical work, which also provides essential insights into ecological management and conservation. I also value the thoroughness of the analysis they conducted.

Without a doubt, this is a highly time-consuming and laborious task, therefore I'll just mention the minor changes below, which are specifically connected to the work limitations and which should be mentioned in the discussion section.

Thank you for your thoughtful and positive feedback about our analysis and data. Your recognition of the value of this research in enhancing our understanding of Australian species and its relevance to conservation is greatly appreciated. Regarding the minor changes and limitations that you've identified, we have acknowledged such aspects in the in the paper to ensure transparency and robustness which we have also included below.

These include;

1. No doubt, MaxEnt perform really good with least number of occurrence points as well, please convey the minimum and maximum number of utilized occurrence points of the considered species in the text.

Thank you for this comment, it's a good idea to include the range of occurrence points somewhere in the manuscript. So, what we have done is calculated a histogram of the total number of occurrence points across species. 95% of species have less than 1000 occurrence points, and there are 6 species (0.05%) that have less than 10 occurrence points. 580 species have more than 1000 occurrence points, and the species with the most occurrence points is the Willie wagtail *(Rhipidura leucophrys)* which has 78,503 occurrence points. The 25th quartile is 43 points, the median number of occurrence points is 123, and the 75th quartile is 410.

We have added the following text into the manuscript:

Line 92: "Across all species, the median number of occurrence points was 123 and the distribution of the number of occurrence points ranged based on the following quantiles: 0%=1, 25%= 43, 50%= 123, 75%= 410, 100%= 78,503 (Figure 2)."

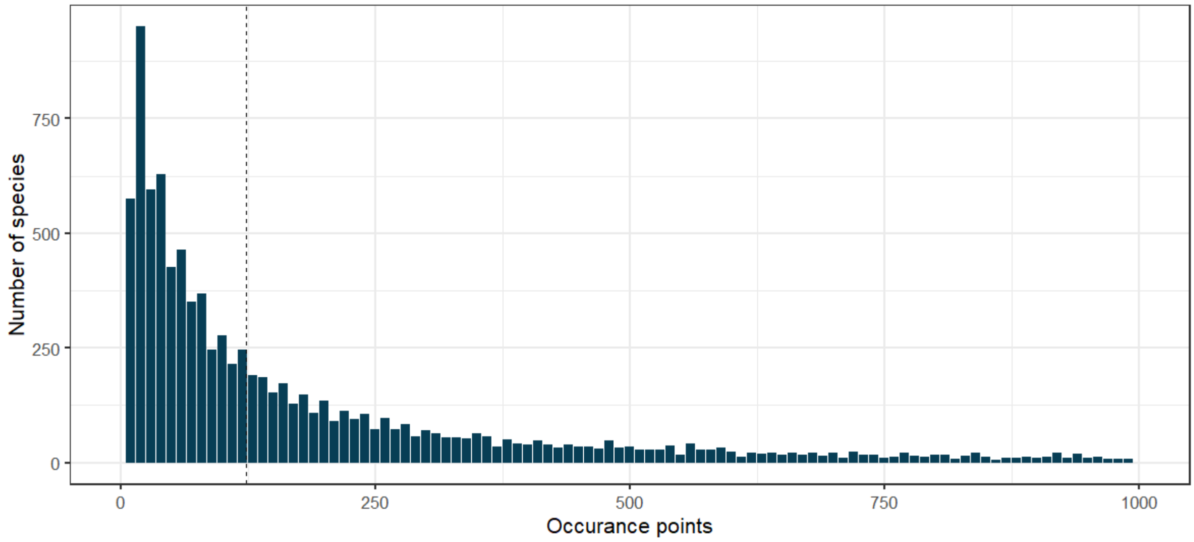

Figure 2 *Distribution of occurrence points (n) for species models.*

2. Line 72-74; and 86-87: Did you clip the environmental data to Australian extent and utilized to extract 10,000 background points for all species? Or clipped the environmental data with

different extent for each species based on minimum and maximum lat/long values of each species? Clarity is missing. Use of a large background especially for those species having least number of occurrence points growing in any unique microhabitat (and in a small geographic land area) influence AUC significantly.

Thank you for raising this point about how the background files were constructed, we recognise that we could have made this much clearer within our methods. To answer your point directly about whether we clipped the environmental data to Australian extent (based on your reference to Lines 86-87). We had provided this information about initial data manipulation and clipping already on:

Line 123: *"All climate scenarios, bioclimatic variables were clipped to the extent of Australia prior to modelling."*

To build on this, we will include more detail on the background file as you have suggested (based on your references to Line 72-74 above). To generate the background files, we did not clip the environmental data further, rather we sampled the background points from only a subset of pixels across Australia based on the occurrence point distribution across taxonomic groups. To create the target group background files, we combined all occurrence points for all species within a taxonomic group, and then sampled the background points from this space. We did this at a taxonomic group level rather than at a species level. This approach of using a target group background file is a way for us to consider spatial biases in Australia.

To address this comment within the manuscript, we have added additional details within the methods section:

Line 98: "*MaxEnt uses background sample points as pseudoabsences and recommends the use of target groups in sample selection to help overcome considered spatial biases* (Phillips et al., 2009). *To create the target group background files, we combined all occurrence points for all species within a taxonomic group and sampled the background points from this space. Each target group background file contained between 60,000 to 250,000 points depending on the taxonomic group, in which MaxEnt takes a subsample of 10,000 points."*

To address your final point about the use of a large background file for species with few occurrence points and this not being able to identify unique microhabitat in a small geographic land area we have added the following text into the methods and limitations section:

Line 473: "*While we did use target background files to reduce spatial biases* (Barber et al., 2022)*, there may still be limitations of this approach at the taxonomic group level, for example for small ranging species* (Breiner et al., 2015)*. Taxonomic level grouping may still be too broad to adequately capture those species that are highly range restricted and require very specific micro-climate needs, therefore species-specific level grouping may help to overcome this. Background files that are too broad may adequately capture sampling biases or the true relationship between occurrence points and environmental predictors."*

3. As you targeted the terrestrial biota mainly, why NDVI or related measures were not considered to be used as important discriminatory variables? Such measures are very important for species residing in dense forests.

We acknowledge that a range of variables like NDVI (Wen et al., 2015) and weather more broadly (Reside et al., 2010) can be important variables to define the distribution of species. However, many of the key studies we drew upon to inform variable selection for the models the habitat suitability of

biodiversity in Australia under climate change did not use NDVI as an explanatory variable (Butt et al., 2013; Gallagher et al., 2019; Graham et al., 2019). Additionally, one major aspect of our study was future projections, and while we used future bioclimatic scenario data from WorldClim, to incorporate NDVI into this analysis, we would have had to model future changes in NDVI, and then use these future projected values within the Maxent analysis. While we did not do this as this was out of the scope of our specific study, many of the bioclimatic variables that we did use in the Maxent models have been reported to be significantly correlated with NDVI (Schwager & Berg, 2021). For example, average temperature, annual precipitation and precipitation seasonality (Ma et al., 2022). To address why we didn't not use why NDVI up-front in the paper we have added the following text within the Limitations:

Line 482: "*In relation to the variables used, we were primarily guided by past efforts that model the suitability of areas across Australia for many species (Butt et al., 2013; Gallagher et al., 2019; Graham et al., 2019), however this approach obviously overlooks some variables that can be import to model suitability. For example, we did not consider variables such as the normalized difference vegetation index (NDVI) (Wen et al., 2015), land use (Lentini & Wintle, 2015), weather (Reside et al., 2010), or detailed information about vegetation structure or extreme events like fire (Eyre et al., 2022).Thus, our recommendation is for the users of this data to consider whether the variables used to model habitat suitability in this study is compatible with the species of interest, or whether additional information is required. This will likely be the case if the user is interested in a more fine-scale application of the data, for example at the single species or local level, as this data is best suited for macro-level analyses and applications.*"

4. To minimize the impact of sampling bias, a bias file (using species occurrence data and environment to estimate and develop a two-dimensional kernel density raster) for each species can be generated and used in MaxEnt distribution modelling. The inclusion of such bias files in the MaxEnt modelling effectively manipulate the background, and introduce the same spatial bias like that which exists in the presence data (Please see; https://aus01.safelinks.protection.outlook.com/?url=https%3A%2F%2Fdoi.org%2F10.3390%2Ff13050715&data=05%7C01%7Cc.archibald%40deakin.edu.au%7Cb83f42e4d5064d41996308dbb018b752%7Cd02378ec168846d585401c28b5f470f6%7C0%7C0%7C638297393738149435%7CUnknown%7CTWFpbGZsb3d8eyJWIjoiMC4wLjAwMDAiLCJQIjoiV2luMzIiLCJBTiI6Ik1haWwiLCJXVCI6Mn0%3D%7C3000%7C%7C%7C&sdata=HNgyy9z%2Fo8lLNLX%2B4wP%2B2SLaGniK5zB6X7kA8KmSwMs%3D&reserved=0).

Thank you for raising this point about spatial biases, we recognise that we could have made this much clearer within our methods. We considered spatial biases in this analysis though using a target group background files which we have addressed in your second comment about the target background files.

To address this comment within the manuscript, we have added additional details within the methods section:

Line 98: "*MaxEnt uses background sample points as pseudoabsences and recommends the use of target groups in sample selection to help overcome considered spatial biases (Philips et al. 2009). To create the target group background files, we combined all occurrence points for all species within a taxonomic group and sampled the background points from this space. Each target group background file contained between 60,000 to 250,000 points depending on the taxonomic group, in which MaxEnt takes a subsample of 10,000 points.*"

5. Though, overall variable importance is communicated, different species have different environmental requirements. I am unable to access the relevant additional/supplementary material at the moment, and want to suggest that please convey the variable importance for highly endangered species (if yet not included in the supplementary data).

The variable importance values for each variable are presented within the Maxent Report files for each species. Therefore, if readers would like to check the individual value for a variable for a species of interest this information on the variable importance is available for the user. Additionally, to your point about endangered species, these listings constantly change and update. So, in the table provided, rather than including the current listings, we have just included an up-dated scientific and common name column which can be joined to relevant listing databases when required by the user.

6. AUC is a widely used metric, but have several inherent flaws, especially for Gaussian point processes, such as MaxEnt. AUC and TSS are frequently criticized by the researchers:
   a. AUC: a misleading measure of the predictive distribution models;
   b. Without quality presence-absence data, discrimination metrics such as TSS can be misleading measures of model performance, hence, partial AUC-ROC might be relatively more appropriate measure.

   Please include all the possible limitations of the work as mentioned above in the discussion section.

Thank you for raising this point about the importance of model validation and raising the challenges of using AUC and TSS to assess model performance. To address the first point about the AUC being a misleading measure, we have better clarified what the AUC assesses within in the Methods section. This ensures the reader is refreshed on what the AUC actual measures. To address the second point, and the comment more generally, about assessing model performance and the role and/or suitability of the AUC as an indicator of this, we have added the presentation of the Boyce Index, as well as expanded section about model validation within the limitations.

Model validation, from Line 211: *"**Model validation***

Once variables were selected, models were re-run, and model performance was assessed based on the area under the curve (AUC, i.e., the area under the receiving operating curve (ROC) curve) and the Boyce index. The AUC is a widely used model validation metric used within the Maxent literature (Merow et al., 2013). The AUC metric measures the predictive accuracy of the model and represents the probability that a randomly selected occurrence point is ranked higher than a randomly selected background point. The Boyce index is another method that can be used to evaluate model performance and does so assessing the magnitude in which the model predictions differ from random distribution of the observed presences across the prediction gradients (Boyce et al., 2002; Hirzel et al., 2006a). The Boyce index value is represented by the Spearman rank correlation coefficient which assesses the increase in the Predicted/Expected (P/E) plot (Jiménez & Soberón, 2020).

The median AUC across all models was 0.97. AUC values of 0.7 or below indicates poor performance (Figure 2), 99.6% (n=10,566) of species have an AUC value above 0.7 AUC, and 0.4% (n=38) of species have an AUC value below the 0.7 threshold (33 birds, 4 vascular plants and 1 mammal). Boyce index values can vary from −1 to 1 and the median Boyce index across all models in this study was 0.97 (Figure 2). A Boyce index closer to 1 indicates that suitability predictions are consistent with the occurrence point distribution, and values of 0.5 or below generally indicate poor performance (Boyce et al., 2002; Hirzel et al., 2006a). 99.3% (n=10,509) of species had a value over 0.5, 0.65% (n=69) species had a value between 0.5 and 0 and 0.05% (n=5) species had a value below 0 (1 bird and 4 vascular plants).

We have also provided a scatter plot summary of AUC in relation to Boyce Index. Based on the 0.7 threshold for AUC and the 0.5 threshold for the Boyce index, we find that 98.99% of species meet both thresholds. We find that 0.69% (n= 73) meet the AUC threshold but not the Boyce Index threshold, 0.32% (n= 34) species meet the Boyce Index threshold but not the AUC threshold and 1 species did not meet either threshold (Brown Falcon, Falco berigora). Prior to using species data, please ensure you check the AUC and the Boyce index value which is contained within the species folder within the maxentResults.csv and the boyce_index_score.csv file.

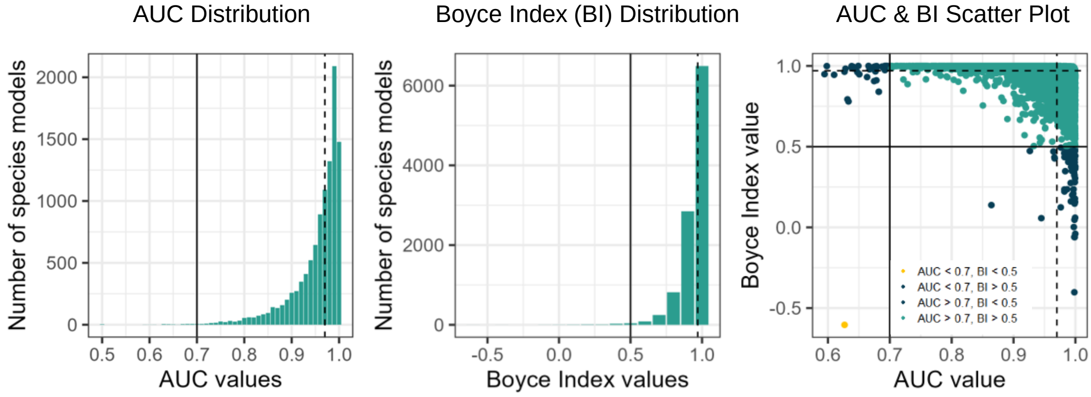

Figure 2 From left to right the plots are the distribution of AUC values, the distribution of Boyce Index (BI) values and a scatter plot between AUC and BI values for species models. The median AUC and Boyce Index value is represented by the dashed vertical line. On the AUC plot the 0.7 threshold is presented using a solid vertical line. On the BI plot the 0.5 threshold is presented using a solid vertical line. These thresholds are also represented by solid lines on the scatter plot."

Finally, in the Limitations section we have also raised this concern:

Limitations, Line 513: *"Finally, there is much contention around the best way to assess model performance of Maxent models beyond just the AUC, to approaches like the True Skill Statistic value (TSS), the kappa score and the Boyce Index* (Allouche et al., 2006; Hirzel et al., 2006; Jiménez & Soberón, 2020; Valavi et al., 2022)*. We present the AUC and the Boyce Index and do not consider the thresholds for these indexes prior to creating the habitat suitability projections, therefore the user can assess the model performance for their species on interest when interpreting the data."*

Reviewer #2:

The manuscript entitled "Habitat suitability maps for Australian flora and fauna under CMIP6 climate scenarios" uses a distribution modeling approach (i.e., Maxent) together with environmental variables to model and map current and future habitat suitability distributions of a large number of flora and fauna species. I enjoyed reading this work; it is nicely written and has proper structure. The drawn conclusions are coherent with the obtained results. This is an important paper that needs to be published to promote conservation and management actions in Australia. Although similar methodologies are common, the results of the study and the provided information could have useful implications for management and conservation actions.

Thank you very much for your positive feedback on our manuscript, "Habitat suitability maps for Australian flora and fauna under CMIP6 climate scenarios." We appreciate your kind words and recognition of the paper's structure, clarity, and the coherence of our conclusions with the obtained results. Your support for the publication of this work is greatly appreciated. We agree that while similar methodologies are prevalent, the unique results and information presented in our paper could

indeed offer valuable insights for practical management and conservation actions. We have addressed the minor changes and limitations that you've identified below.

Abstract

- Please outline the objective of the study clearly.

We have edited this sentence to now read:

Abstract, Line 6: "The objective of this paper is to develop habitat suitability maps for Australian flora and fauna under different climate futures."

- Please add some information on how suitable habitats would change by comparing current areas with future areas.

We have now added some information on how suitable habitats may change, this section now reads:

Abstract, Line 13: "Conclusions: The spatial data supplied can help identify important and sensitive locations for species under various climate futures. Additionally, the supplied tabular data can provide insights into the impacts of climate change on biodiversity in Australia. These habitat suitability maps can be used as input data for landscape and conservation planning or species management, particularly under different climate change scenarios in Australia."

Methods

Line 81-82: I suggest citing the GCMs.

Line 124: "We have added the references to each GCM, "Bioclimatic variables were downloaded for eight global climate models (GCMs): BCC-CSM2-MR (Wu et al., 2021)*, CNRM-CM6-1* (Voldoire et al., 2019)*, CNRM-ESM2-1* (Séférian et al., 2019)*, CanESM5* (Swart et al., 2019)*, GFDL-ESM4* (Krasting et al., 2018)*, IPSL-CM6A-LR* (Boucher et al., 2020)*, MIROC-ES2L* (Hajima et al., 2020)*, MIROC6* (Tatebe et al., 2018)*, MRI-ESM2-0* (Yukimoto et al., 2019)"

See the reference list at the end of this document for the full references.

Line 96: "Model fitting" Please outline the model setting of Maxent. Model parameterization is important, yet minimal information is provided.

Thank you for to this point about needing to clarify the model fitting section. To provide you with a clear understanding of the call here, we have presented the exact Maxent command line call below, which is also provided in the GitHub repository for this study.

Initial cross validation step:

```
java -mx2048m -jar /maxent.jar -e background.csv -s occurance.csv -o
/species_folder nothreshold nowarnings novisible allowpartialdata
replicates=10 nooutputgrids -r -a
```

Model fitting:

```
java -mx2048m -jar / maxent.jar -e /background.csv -s / occurance.csv -o
/species_folder nothreshold nowarnings novisible nowriteclampgrid
nowritemess allowpartialdata writeplotdata -P -J -r -a
```

To address this comment within the paper, we have edited the model fitting section to now read:

Line 154: "*All habitat suitability models were fit in MaxEnt Version 3.4.1 using the command line. Maxent models were first run with 10 replicates (replicates=10) validated using a cross validation method to train the model and to compute model validation statistics. At this stage, habitat suitability values are calculated as values between 0 and 1 with no threshold applied and were later converted to values between 0 and 100. An example of the full Maxent model specification can be found in the GitHub repository affiliated with this paper. Important outputs of the MaxEnt modelling procedure include a .csv file containing statistical information to inform variable selection and model validation as well as the 'lambdas file', which is a text file containing the regression coefficients or lambdas fit by MaxEnt during modelling.*"

Line 136: "AUC", AUC alone is sometimes not sufficient to evaluate the performance of the model; other metrics such as Kappa and TSS are also important.

Thank you for raising this point about the importance of model validation and raising the challenges of using AUC and TSS to assess model performance. To address the first point about the AUC being a misleading measure, we have better clarify what the AUC assess within in the Methods section. This ensures the reader is refreshed on what the AUC actual measures:

Methods, Line 214: "*Once variables were selected, models were re-run, and model performance was assessed based on the area under the curve (AUC, i.e., the area under the receiver operating curve (ROC) curve) and the Boyce index. The AUC is a widely used model validation metric used within the Maxent literature (Merow et al., 2013). The AUC metric measures the predictive accuracy of the model and represents the probability that a randomly selected occurrence point is ranked higher than a randomly selected background point.*"

To address the second point, and the comment more generally, about assessing model performance and the role and/or suitability of the AUC as an indicator of this, we have added the presentation of the Boyce Index, as well as expanded section about model validation within the limitations.

Model validation, from Line 211: "***Model validation***

Once variables were selected, models were re-run, and model performance was assessed based on the area under the curve (AUC, i.e., the area under the receiving operating curve (ROC) curve) and the Boyce index. The AUC is a widely used model validation metric used within the Maxent literature (Merow et al., 2013). The AUC metric measures the predictive accuracy of the model and represents the probability that a randomly selected occurrence point is ranked higher than a randomly selected background point. The Boyce index is another method that can be used to evaluate model performance and does so assessing the magnitude in which the model predictions differ from random distribution of the observed presences across the prediction gradients (Boyce et al., 2002; Hirzel et al., 2006a). The Boyce index value is represented by the Spearman rank correlation coefficient which assesses the increase in the Predicted/Expected (P/E) plot (Jiménez & Soberón, 2020).

The median AUC across all models was 0.97. AUC values of 0.7 or below indicates poor performance (Figure 2), 99.6% (n=10,566) of species have an AUC value above 0.7 AUC, and 0.4% (n=38) of species have an AUC value below the 0.7 threshold (33 birds, 4 vascular plants and 1 mammal). Boyce index values can vary from −1 to 1 and the median Boyce index across all models in this study was 0.97 (Figure 2). A Boyce index closer to 1 indicates that suitability predictions are consistent with the occurrence point distribution, and values of 0.5 or below generally indicate poor performance (Boyce et al., 2002; Hirzel et al., 2006a). 99.3% (n=10,509) of species had a value over 0.5, 0.65% (n=69) species had a value between 0.5 and 0 and 0.05% (n=5) species had a value below 0 (1 bird and 4 vascular plants).

We have also provided a scatter plot summary of AUC in relation to Boyce Index. Based on the 0.7 threshold for AUC and the 0.5 threshold for the Boyce index, we find that 98.99% of species meet both thresholds. We find that 0.69% (n= 73) meet the AUC threshold but not the Boyce Index threshold, 0.32% (n= 34) species meet the Boyce Index threshold but not the AUC threshold and 1 species did not meet either threshold (Brown Falcon, Falco berigora). Prior to using species data, please ensure you check the AUC and the Boyce index value which is contained within the species folder within the maxentResults.csv and the boyce_index_score.csv file.

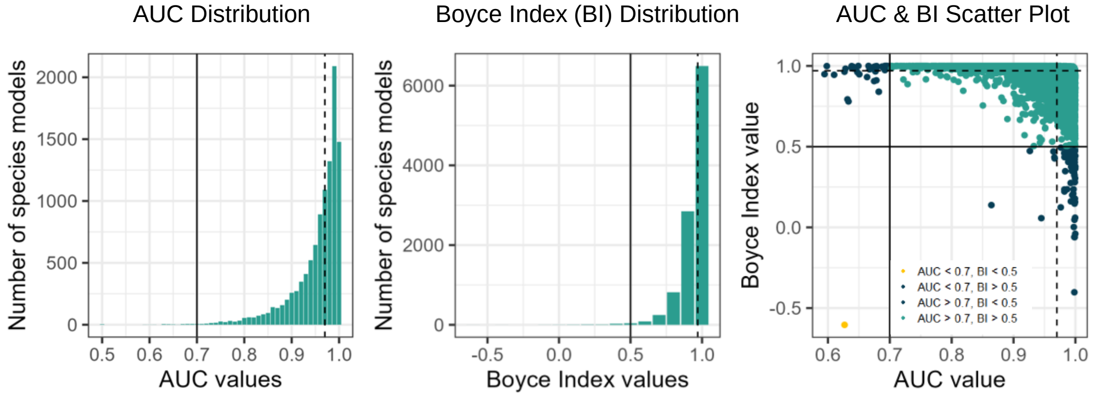

Figure 2 From left to right the plots are the distribution of AUC values, the distribution of Boyce Index (BI) values and a scatter plot between AUC and BI values for species models. The median AUC and Boyce Index value is represented by the dashed vertical line. On the AUC plot the 0.7 threshold is presented using a solid vertical line. On the BI plot the 0.5 threshold is presented using a solid vertical line. These thresholds are also represented by solid lines on the scatter plot."

Finally, in the Limitations section we have also raised this concern:

Limitations, Line 513: *"Finally, there is much contention around the best way to assess model performance of Maxent models beyond just the AUC, to approaches like the True Skill Statistic value (TSS), the kappa score and the Boyce Index* (Allouche et al., 2006; Hirzel et al., 2006; Jiménez & Soberón, 2020; Valavi et al., 2022)*. We present the AUC and the Boyce Index and do not consider the thresholds for these indexes prior to creating the habitat suitability projections, therefore the user can assess the model performance for their species on interest when interpreting the data."*

Line 238: "subtracted from one another to accentuate areas". Reporting some information on how the suitability areas would change is suggested.

Thank you for this point, we will clarify where we have presented information on how suitability areas would change over time. We have slightly clarified the wording to ensure clarity, and have changed to:

Lines 354: *"To calculate spatial changes in habitat suitability through time, historical suitability maps can be subtracted from future suitability maps to spatially accentuate locations that have changed in habitat suitability (i.e., improved in suitability or declined in suitability) across time periods."*

Discussion
A brief paragraph on the implications of the applied technique and the huge data could be useful. In addition, how the applied technique and provided data aid in establishing priority zones for conservation action is suggested. https://doi.org/10.1016/j.ecoinf.2022.101930

Thank you for raising the need to better emphasise the use potential in the context of additional applied techniques to work with the data. To address this comment, we firstly wanted to point to the purpose of the "Re-use potential" potential sections and the role of the "Spatial resolution of data", "Species-level data summary" and the "Spatial changes over time" sections to describe the initial reuse potential of the data. To extend these sections and provide additional recommendations on reuse potential we will better emphasise the use and various potential applied techniques to work with the data in the Discussion.

Discussion, from Line 392:

"Applications for landscape and species conservation

This spatial and tabular dataset is ideal for users that would like to understand how the habitat suitability of areas for species is predicted to change over time or under different climate scenarios. For example, at the landscape level, these habitat suitability maps can be combined into a general biodiversity layer to evaluate how habitat suitability more generally changes over time (Figure 6) or over space and time (Figure 5) (Hama & Khwarahm, 2023). This data can then be utilized in applications such as spatial prioritizations using such tools as Zonation (Minin et al., 2014) or Marxan (Watts et al., 2009) to guide spatial conservation priorities in Australia (Maxwell et al., 2019; Summers et al., 2012b). Therefore, can be useful to inform conservation (e.g., where to establish new protected areas), restoration or monitoring plans in areas which are suitable for biodiversity, or are predicted to lose or gain suitable areas for biodiversity.

At the species level, this data set can be used to support conservation actions for species of interest (e.g., threatened species, iconic species, endemic species). The tabular data can be used to systematically identify species of interest based on the way climate change is anticipated to impact the species. Or could be used to inform processes such as threatened species listing (IUCN, 2022). Spatial information about species could also be useful to compare the long-term suitability of areas for threatened species under climate change to inform present day decision-making and species management (Harley, 2023; Hawke et al., 2020). Could be paired with other types of data to assess the impacts of climate change on species (Eyre et al., 2022). Or could inform boarder scale biodiversity conservation analyses (Engert et al., 2023).

Applications in sustainability and natural capital accounting

Biodiversity forms a foundation of broader sustainability ideals, therefore, to measure progress towards sustainability, conservation or corporate goals spatial data on biodiversity can serve as an important input infomration to the creation of metrics (Lamb et al., 2009; Watermeyer et al., 2021). Biodiversity indicators like the species richness, or more complex indicators like the Species Threat Abatement and Restoration metric (STAR) (Mair et al., 2021) or the biodiversity intactness index (BII) (Biggs & Scholes, 2005) all draw from species layers as input data. Feeding the habitat suitability maps generated in this study into biodiversity layers and into broader sustainability models or assessments can improve the consideration of biodiversity against other environmental or social values. This may include initiatives such as land use planning, or land use change modelling (Gao & Bryan, 2017; Stock et al., 2013).

Additionally, as many businesses are transitioning towards 'nature positive' the use of biodiversity to monitor business impacts and progress towards nature positive is necessary. The habitat suitability maps generated in this study can be used to represent key species or biodiversity within natural capital within frameworks such as the System of Environmental-Economic Accounting (SEEA) framework (UNEP et al., 2015), or could be used within sustainability assessments such as 'foot printing' to updates the biodiversity input data (Halpern et al., 2022; Hoang et al., 2023; Irwin & Geschke, 2023), or could be used to help assess organisational impacts or dependencies to

biodiversity or to specific species within frameworks like the Taskforce on Nature-Related Financial Disclosures (TNFD) (TNFD, 2023)."

Reviewer #3:

This manuscript presents habitat suitability of Australian flora and fauna under future climate change. The authors produced habitat suitability maps under RCP2.6-SSP1, RCP4.5-SSP2, RCP7.0-SSP3 and RCP8.5-SSP5 climate futures for 1,382 terrestrial vertebrates and 9,251 vascular Plants. The research can be used for landscape and conservation planning or species management, particularly under different climate change scenarios in Australia contribute towards evidence-based decision-making regarding demarcation of protected areas in Iran. Overall, the manuscript is well written with sufficient presentation of research aims, methodologies, key results and outcomes. I recommend this manuscript for publication in GigaScience.

Thank you for this overwhelmingly supportive review. We appreciate your shared vision of the potential importance and role of this data in real-world applications of landscape and conservation planning or species management in Australia.

Reference List

Allouche, O., Tsoar, A., & Kadmon, R. (2006). Assessing the accuracy of species distribution models: prevalence, kappa and the true skill statistic (TSS). *Journal of Applied Ecology*, *43*(6), 1223–1232. https://doi.org/10.1111/j.1365-2664.2006.01214.x

Barber, R. A., Ball, S. G., Morris, R. K. A., & Gilbert, F. (2022). Target-group backgrounds prove effective at correcting sampling bias in Maxent models. *Diversity and Distributions*, *28*(1), 128–141. https://doi.org/10.1111/ddi.13442

Biggs, R., & Scholes, R. J. (2005). A biodiversity intactness index. *Nature*, *434*(7029), 45–49. http://go.galegroup.com/ps/i.do?id=GALE%7CA185471773&v=2.1&u=ntu&it=r&p=AONE&sw=w

Boucher, O., Servonnat, J., Albright, A. L., Aumont, O., Balkanski, Y., Bastrikov, V., Bekki, S., Bonnet, R., Bony, S., Bopp, L., Braconnot, P., Brockmann, P., Cadule, P., Caubel, A., Cheruy, F., Codron, F., Cozic, A., Cugnet, D., D'Andrea, F., … Vuichard, N. (2020). Presentation and Evaluation of the IPSL-CM6A-LR Climate Model. *Journal of Advances in Modeling Earth Systems*, *12*(7), 1–52. https://doi.org/10.1029/2019MS002010

Breiner, F. T., Guisan, A., Bergamini, A., & Nobis, M. P. (2015). Overcoming limitations of modelling rare species by using ensembles of small models. *Methods in Ecology and Evolution*, *6*(10), 1210–1218. https://doi.org/10.1111/2041-210X.12403

Butt, N., Pollock, L. J., & Mcalpine, C. A. (2013). Eucalypts face increasing climate stress. *Ecology and Evolution*, *3*(15), 5011–5022. https://doi.org/10.1002/ece3.873

Engert, J. E., Pressey, R. L., & Adams, V. M. (2023). Threatened fauna protections compromised by agricultural interests in Australia. *Conservation Letters*. https://doi.org/10.1111/conl.12975

Eyre, A. C., Briscoe, N. J., Harley, D. K. P., Lumsden, L. F., McComb, L. B., & Lentini, P. E. (2022). Using species distribution models and decision tools to direct surveys and identify potential translocation sites for a critically endangered species. *Diversity and Distributions*, *28*(4), 700–711. https://doi.org/10.1111/ddi.13469

Gallagher, R. V., Allen, S., & Wright, I. J. (2019). Safety margins and adaptive capacity of vegetation to climate change. *Scientific Reports*, *9*(1), 1–11. https://doi.org/10.1038/s41598-019-44483-x

Gao, L., & Bryan, B. A. (2017). Finding pathways to national-scale land-sector sustainability. *Nature*, *544*(7649), 217–222. https://doi.org/10.1038/nature21694

Graham, E. M., Reside, A. E., Atkinson, I., Baird, D., Hodgson, L., James, C. S., & VanDerWal, J. J. (2019). Climate change and biodiversity in Australia: a systematic modelling approach to

nationwide species distributions. *Australasian Journal of Environmental Management*, *26*(2), 112–123. https://doi.org/10.1080/14486563.2019.1599742

Hajima, T., Watanabe, M., Yamamoto, A., Tatebe, H., Noguchi, M. A., Abe, M., Ohgaito, R., Ito, A., Yamazaki, D., Okajima, H., Ito, A., Takata, K., Ogochi, K., Watanabe, S., & Kawamiya, M. (2020). Development of the MIROC-ES2L Earth system model and the evaluation of biogeochemical processes and feedbacks. *Geoscientific Model Development*, *13*(5), 2197–2244. https://doi.org/10.5194/gmd-13-2197-2020

Halpern, B. S., Frazier, M., Verstaen, J., Rayner, P. E., Clawson, G., Blanchard, J. L., Cottrell, R. S., Froehlich, H. E., Gephart, J. A., Jacobsen, N. S., Kuempel, C. D., McIntyre, P. B., Metian, M., Moran, D., Nash, K. L., Többen, J., & Williams, D. R. (2022). The environmental footprint of global food production. *Nature Sustainability*, *5*(12), 1027–1039. https://doi.org/10.1038/s41893-022-00965-x

Hama, A. A., & Khwarahm, N. R. (2023). Predictive mapping of two endemic oak tree species under climate change scenarios in a semiarid region: Range overlap and implications for conservation. *Ecological Informatics*, *73*, 101930. https://doi.org/10.1016/j.ecoinf.2022.101930

Harley, D. (2023). Seven urgent actions to prevent the extinction of the critically endangered Leadbeater's possum (Gymnobelideus leadbeateri). *Pacific Conservation Biology*, *29*(5), 387–395. https://doi.org/10.1071/PC22021

Hawke, T., Bino, G., Kingsford, R. T., Grant, T., Griffiths, J., Weeks, A., Tingley, R., Mccoll-Gausden, E., Serena, M., Williams, G., Brunt, T., Mijangos, L., Sherwin, W., & Noonan, J. (2020). *A national assessment of the conservation status of the platypus*.

Hirzel, A. H., Le Lay, G., Helfer, V., Randin, C., & Guisan, A. (2006). Evaluating the ability of habitat suitability models to predict species presences. *Ecological Modelling*, *199*(2), 142–152. https://doi.org/10.1016/j.ecolmodel.2006.05.017

Hoang, N. T., Taherzadeh, O., Ohashi, H., Yonekura, Y., Nishijima, S., Yamabe, M., Matsui, T., Matsuda, H., Moran, D., & Kanemoto, K. (2023). Mapping potential conflicts between global agriculture and terrestrial conservation. *Proceedings of the National Academy of Sciences of the United States of America*, *120*(23). https://doi.org/10.1073/pnas.2208376120

Irwin, A., & Geschke, A. (2023). A consumption-based analysis of extinction risk in Australia. *Conservation Letters*, *16*(3). https://doi.org/10.1111/conl.12942

IUCN. (2022). *The IUCN Red List of Threatened Species (IUCN)*. http://www.iucnredlist.org

Jiménez, L., & Soberón, J. (2020). Leaving the area under the receiving operating characteristic curve behind: An evaluation method for species distribution modelling applications based on presence-only data. *Methods in Ecology and Evolution*, *11*(12), 1571–1586. https://doi.org/10.1111/2041-210X.13479

Krasting, J. P., John, J. G., Blanton, C., McHugh, C., Nikonov, S., Radhakrishnan, A., Rand, K., Zadeh, N. T., Balaji, V., Durachta, J., Dupuis, C., Menzel, R., Robinson, T., Underwood, S., Vahlenkamp, H., Dunne, K. A., Gauthier, P. P., Ginoux, P., Griffies, S. M., … Zhao, M. (2018). *NOAA-GFDL GFDL-ESM4 model output prepared for CMIP6 CMIP*.

Lamb, E. G., Bayne, E., Holloway, G., Schieck, J., Boutin, S., Herbers, J., & Haughland, D. L. (2009). Indices for monitoring biodiversity change: Are some more effective than others? *Ecological Indicators*, *9*(3), 432–444. https://doi.org/10.1016/j.ecolind.2008.06.001

Lentini, P. E., & Wintle, B. A. (2015). Spatial conservation priorities are highly sensitive to choice of biodiversity surrogates and species distribution model type. *Ecography*, *38*(11), 1101–1111. https://doi.org/10.1111/ecog.01252

Ma, B., Zeng, W., Hu, G., Cao, R., Cui, D., & Zhang, T. (2022). Normalized difference vegetation index prediction based on the delta downscaling method and back-propagation artificial neural network under climate change in the Sanjiangyuan region, China. *Ecological Informatics*, *72*, 101883. https://doi.org/10.1016/j.ecoinf.2022.101883

Mair, L., Bennun, L. A., Brooks, T. M., Butchart, S. H. M., Bolam, F. C., Burgess, N. D., Ekstrom, J. M. M., Milner-Gulland, E. J., Hoffmann, M., Ma, K., Macfarlane, N. B. W., Raimondo, D. C.,

Rodrigues, A. S. L., Shen, X., Strassburg, B. B. N., Beatty, C. R., Gómez-Creutzberg, C., Iribarrem, A., Irmadhiany, M., … McGowan, P. J. K. (2021). A metric for spatially explicit contributions to science-based species targets. *Nature Ecology and Evolution*, *5*(6), 836–844. https://doi.org/10.1038/s41559-021-01432-0

Maxwell, S. L., Reside, A., Trezise, J., McAlpine, C. A., & Watson, J. E. (2019). Retention and restoration priorities for climate adaptation in a multi-use landscape. *Global Ecology and Conservation*, *18*, e00649. https://doi.org/10.1016/j.gecco.2019.e00649

Minin, E., Veach, V., Lehtomäki, J., Pouzols, F. M., & Moilanen, A. (2014). *A quick introduction to Zonation. Version 1 (for Zv4). User Manual. 1*, 1–30. http://cbig.it.helsinki.fi/files/zonation/Z_quick_intro_manual_B5_final_3.pdf

Phillips, S. J., Dudík, M., Elith, J., Graham, C. H., Lehmann, A., Leathwick, J., & Ferrier, S. (2009). Sample selection bias and presence-only distribution models: Implications for background and pseudo-absence data. *Ecological Applications*, *19*(1), 181–197. https://doi.org/10.1890/07-2153.1

Reside, A. E., Vanderwal, J. J., Kutt, A. S., & Perkins, G. C. (2010). Weather, Not Climate, Defines Distributions of Vagile Bird Species. *PLoS ONE*, *5*(10), 1–9. https://doi.org/10.1371/journal.pone.0013569

Schwager, P., & Berg, C. (2021). Remote sensing variables improve species distribution models for alpine plant species. *Basic and Applied Ecology*, *54*, 1–13. https://doi.org/10.1016/j.baae.2021.04.002

Séférian, R., Nabat, P., Michou, M., Saint-Martin, D., Voldoire, A., Colin, J., Decharme, B., Delire, C., Berthet, S., Chevallier, M., Sénési, S., Franchisteguy, L., Vial, J., Mallet, M., Joetzjer, E., Geoffroy, O., Guérémy, J. F., Moine, M. P., Msadek, R., … Madec, G. (2019). Evaluation of CNRM Earth System Model, CNRM-ESM2-1: Role of Earth System Processes in Present-Day and Future Climate. *Journal of Advances in Modeling Earth Systems*, *11*(12), 4182–4227. https://doi.org/10.1029/2019MS001791

Stock, F., Bryan, B., Nolan, M., Graham, P., Connor, J., Dunstall, S., Ernst, A., Stock, F., Nolan, M., Bryan, B., Graham, P., Connor, J., Dunstall, S., & Ernst, A. (2013). *LUTO - Land Use Trade-Offs National modeling of land use choices and pathways*.

Swart, N. C., Cole, J. N. S., Kharin, V. V., Lazare, M., Scinocca, J. F., Gillett, N. P., Anstey, J., Arora, V., Christian, J. R., Hanna, S., Jiao, Y., Lee, W. G., Majaess, F., Saenko, O. A., Seiler, C., Seinen, C., Shao, A., Sigmond, M., Solheim, L., … Winter, B. (2019). The Canadian Earth System Model version 5 (CanESM5.0.3). *Geoscientific Model Development*, *12*(11), 4823–4873. https://doi.org/10.5194/gmd-12-4823-2019

Tatebe, H., Ogura, T., Nitta, T., Komuro, Y., Ogochi, K., Takemura, T., Sudo, K., Sekiguchi, M., Abe, M., Saito, F., Chikira, M., Watanabe, S., Mori, M., Hirota, N., Kawatani, Y., Mochizuki, T., Yoshimura, K., Takata, K., O'ishi, R., … Kimoto, M. (2018). Description and basic evaluation of simulated mean state, internal variability, and climate sensitivity in MIROC6. *Geoscientific Model Development Discussions*, 1–92. https://doi.org/10.5194/gmd-2018-155

TNFD. (2023). *Recommendations of the Taskforce on Nature-related Financial Disclosures*.

UNEP, UNSD, CBD, & NORAD. (2015). *SEEA Experimental Ecosystem Accounting: Technical Recommendations* (Issue December).

Valavi, R., Guillera-Arroita, G., Lahoz-Monfort, J. J., & Elith, J. (2022). Predictive performance of presence-only species distribution models: a benchmark study with reproducible code. *Ecological Monographs*, *92*(1). https://doi.org/10.1002/ecm.1486

Voldoire, A., Saint-Martin, D., Sénési, S., Decharme, B., Alias, A., Chevallier, M., Colin, J., Guérémy, J. F., Michou, M., Moine, M. P., Nabat, P., Roehrig, R., Salas y Mélia, D., Séférian, R., Valcke, S., Beau, I., Belamari, S., Berthet, S., Cassou, C., … Waldman, R. (2019). Evaluation of CMIP6 DECK Experiments With CNRM-CM6-1. *Journal of Advances in Modeling Earth Systems*, *11*(7), 2177–2213. https://doi.org/10.1029/2019MS001683

Watermeyer, K. E., Guillera-Arroita, G., Bal, P., Burgass, M. J., Bland, L. M., Collen, B., Hallam, C., Kelly, L. T., McCarthy, M. A., Regan, T. J., Stevenson, S., Wintle, B. A., & Nicholson, E. (2021). Using decision science to evaluate global biodiversity indices. *Conservation Biology*, *35*(2), 492–501. https://doi.org/10.1111/cobi.13574

Watts, M. E., Ball, I. R., Stewart, R. S., Klein, C. J., Wilson, K., Steinback, C., Lourival, R., Kircher, L., & Possingham, H. P. (2009). Marxan with Zones: Software for optimal conservation based land- and sea-use zoning. *Environmental Modelling and Software*, *24*(12), 1513–1521. https://doi.org/10.1016/j.envsoft.2009.06.005

Wen, L., Saintilan, N., Yang, X., Hunter, S., & Mawer, D. (2015). MODIS NDVI based metrics improve habitat suitability modelling in fragmented patchy floodplains. *Remote Sensing Applications: Society and Environment*, *1*, 85–97. https://doi.org/10.1016/j.rsase.2015.08.001

Wu, T., Yu, R., Lu, Y., Jie, W., Fang, Y., Zhang, J., Zhang, L., Xin, X., Li, L., Wang, Z., Liu, Y., Zhang, F., Wu, F., Chu, M., Li, J., Li, W., Zhang, Y., Shi, X., Zhou, W., … Hu, A. (2021). BCC-CSM2-HR: A high-resolution version of the Beijing Climate Center Climate System Model. *Geoscientific Model Development*, *14*(5), 2977–3006. https://doi.org/10.5194/gmd-14-2977-2021

Yukimoto, S., Kawai, H., Koshiro, T., Oshima, N., Yoshida, K., Urakawa, S., Tsujino, H., Deushi, M., Tanaka, T., Hosaka, M., Yabu, S., Yoshimura, H., Shindo, E., Mizuta, R., Obata, A., Adachi, Y., & Ishii, M. (2019). The meteorological research institute Earth system model version 2.0, MRI-ESM2.0: Description and basic evaluation of the physical component. *Journal of the Meteorological Society of Japan*, *97*(5), 931–965. https://doi.org/10.2151/jmsj.2019-051

Cover letter

Click here to access/download;Personal Cover;Cover_Letter.pdf

Deakin University
School of Life and Environmental Sciences

DEAKIN UNIVERSITY

Dear Hongling Zhou, Dr Laurie Goodman and Dr Scott Edmunds Nov 2023
GigaScience

Thank you for three constructive reviews and your feedback on this manuscript '**Habitat suitability maps for Australian flora and fauna under CMIP6 climate scenarios**' (GIGA-D-23-00183). We note the 12 key points of feedback raised by the reviewers and have addressed them line-by-line in the attached response document. We are confident that the improvements that we have made to the manuscript make this study a much more robust and useful contribution to the literature, and we look forward to your response. I wish to resubmit this Data Note which compliments the dataset 'Supporting data for "Habitat suitability maps for Australian flora and fauna under CMIP6 climate scenarios"' (to be hosted on *GigaDB)*, on behalf of my co-authors for consideration for publication in the journal *GigaScience.*

Summary of the manuscript

The aim of this data compilation effort was to quantify the habitat suitability of Australian flora and fauna species under climate change and over time and provide the data on an open-access platform, such as *GigaScience*. This Data Note outlines the significance, methodology and reuse potential for this exceptional dataset in the field of conservation science in a manner to encourage reuse:

- **Background:** Australia is a global biodiversity hotspot, however, developing continental-scale data for numerous species poses challenges due to the computational resources requirements as well as requires the handling and storing large datasets.
- **Dataset size:** The data set we present to is approximately 60GB in size and is comprised mainly of compressed raster images, as well as tabular and text file data. This dataset includes species-level historical and future, minimum, mean and maximum habitat suitability projections for 1,382 terrestrial vertebrates and 9,251 vascular plants under 4 climate scenarios and 5 time periods, this data equates to 521,017 rasters.
- **FAIR (Findable, Accessible, Interoperable and Reusable) principles**: This project strives to be 'findable' and 'accessible' by being published in an open sources database such as *GigaScience* and *GigaDB*. We also stive for the data to be 'interoperable' through the publication of this data note as well as by providing a GitHub repository for the affiliated scripts. Finally, we aim that this data is 'reusable' as the resuse potential has been described in the paper, and we are hosting the data on GigaDB.
- **Novelty of data**: Currently there is no spatial data product available open access that provides information about habitat suitability for Australian species under climate change. In 2017, Graham et al. (2019) published a comprehensive spatial dataset of species distributions known as the CliMAS, however, the data portal was retired in 2020 due to updates in IPCC projections (CMIP6). This has left a gap in the biodiversity conservation data landscape in Australia.
- **Reuse potential:** The data are well documented in the Data Note, and we have included extensive metadata in the files sheet in the GigaDB repository. We provide unique information allowing novel uses of data which include spatial and temporal analysis of species as well as Australian. Additionally, the original CliMAS dataset, available through the web portal CliMAS, yielded 7,559 unique pageviews over a four-year period and many practical outcomes published in academic journals. Therefore, we are confident the data we are presented to *GigaScience* will have similar academic and practical application.

How we updated the manuscript based on the Reviewer Feedback

We have greatly improved this manuscript based on the three constructive reviews we received from *GigaScience*. Overall, we appreciate the overwhelmingly supportive reviews and the shared vision of

Wilkinson, M. D., et al. (2016). The FAIR Guiding Principles for scientific data management and stewardship. Scientific Data, 3

Graham, E. M., et al. (2019). Climate change and biodiversity in Australia: a systematic modelling approach to nationwide species distributions. *Australasian Journal of Environmental Management*, *26*(2), 112–123

the potential importance and role of this data in real-world applications of landscape and conservation planning or species management in Australia. We have addressed all minor and major feedback outlined by the reviewers, which is presented in the Reviewer Report with line-by-line changes we have made.

Major feedback which was noted by both Reviewer 1 and Reviewer 2 was to *improve the model validation* section of the Data Note. To do this we calculated an additional indicator of model performance known as the Boyce Index. We also provided additional figures to display the range of AUC and Boyce Index values present in the dataset which will help the users of the data assess model performance.

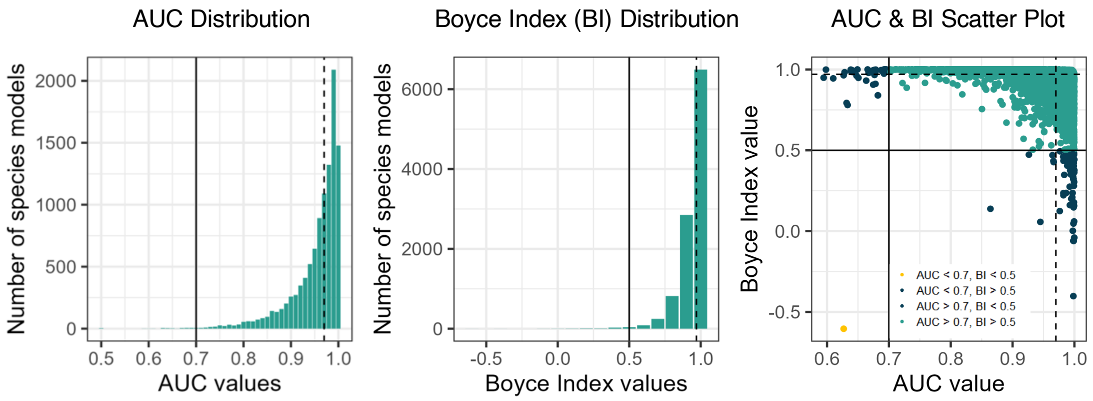

Figure 3 in the Manuscript: From left to right the plots are the distribution of AUC values, the distribution of Boyce Index (BI) values and a scatter plot between AUC and BI values for species models. The median AUC and Boyce Index value is represented by a dashed line. On the AUC plot the 0.7 threshold is presented using a solid vertical line. On the BI plot the 0.5 threshold is presented using a solid vertical line. These thresholds are also represented by solid lines on the scatter plot.

An additional major comment was to add more detail on how the data can be applied and used in practice. To address this comment, we have added two sections to the discussion (Discussion, from Line 392) titled "*Applications for landscape and species conservation*" and "*Applications in sustainability and natural capital accounting*" to clearly outline various use cases for the data.

All other minor comments related to clarifying the distribution of occurrence points, better explaining the acronyms, clarifying how some aspects of the methods and the calculations are described and improving the abstract have been addressed.

We confirm that this paper has not in whole or in part been published previously and is not currently under consideration for publication elsewhere. We look forward to your response.

Dr Carla Archibald, on behalf of all co-authors.
School of Life and Environmental Sciences
Deakin University
c.archibald@deakin.edu.au
