# Supplementary material for: Habitat suitability maps for Australian flora and fauna under CMIP6 climate scenarios
Source: Gigascience. 2024 Mar 5;13:giae002. doi: 10.1093/gigascience/giae002 (PMC10939329; doi:10.1093/gigascience/giae002)
Supplement: giae002_Response_to_Reviewer_Comments_Original_Submission [file giae002_response_to_reviewer_comments_original_submission.pdf]

Author's Response To Reviewer Comments

Hello, we have provided our Reviewer Report as a word document, please refer to this document as it contains updated figure

---

Associate Editor

Dear Carla,

Your manuscript "Habitat suitability maps for Australian flora and fauna under CMIP6 climate scenarios" (GIGA-D-23-00183)
revised version to be published in GigaScience.

Their reports, together with any other comments, are below. Please also take a moment to check our website at
https://aus01.safelinks.protection.outlook.com/?url=https%3A%2F%2Fwww.editorialmanager.com%2Fgiga%2F&data=05%7
CJBTiI6Ik1haWwiLCJXVCI6Mn0%3D%7C3000%7C%7C%7C&sdata=OVNQPHhHe9gvRwMTeo3zMAZJrUvc5C2G5yKxN9YUZlA%

In addition, please register any new software application in the bio.tools and SciCrunch.org databases to receive RRID (Resea
facilitate tracking, reproducibility and re-use of your tool.

If you are able to fully address these points, we would encourage you to submit a revised manuscript to GigaScience. Once y

https://aus01.safelinks.protection.outlook.com/?url=https%3A%2F%2Fwww.editorialmanager.com%2Fgiga%2F&data=05%7
CJBTiI6Ik1haWwiLCJXVCI6Mn0%3D%7C3000%7C%7C%7C&sdata=OVNQPHhHe9gvRwMTeo3zMAZJrUvc5C2G5yKxN9YUZlA%

If you have forgotten your username or password please use the "Send Login Details" link to get your login information. For s

Please include a point-by-point within the 'Response to Reviewers' box in the submission system. Please ensure you describe
the Instructions for Authors on the journal homepage. If the data and code has been modified in the revision process please l

The due date for submitting the revised version of your article is 06 Dec 2023.

I look forward to receiving your revised manuscript soon.

Best wishes,

Hongling Zhou
GigaScience
https://aus01.safelinks.protection.outlook.com/?url=http%3A%2F%2Fwww.gigasciencejournal.com%2F&data=05%7C01%7
Ik1haWwiLCJXVCI6Mn0%3D%7C3000%7C%7C%7C&sdata=nq1%2BnH5afs0LilMaNqUnsiJLEIhZr4%2BIytT9lTJoYfQ%3D&res

Dear Hongling Zhou,

Thank you for these three constructive reviews and your feedback on this manuscript, "Habitat suitability maps for Australian

We have added additional information about the occurrence point data used, and have addressed the concerns about sample
model performance [as well as added one additional indicator of model performance]. Reviewer 2 also emphasised to value o
natural capital accounting". Overall, we have also greatly expanded the limitations and caveats section to ensure that these e

We are confident that these changes to the manuscript make this study a much more robust and useful contribution to the lit

Sincerely,
Carla Archibald

Reviewer reports:

Reviewer #1:

Title: Habitat suitability maps for Australian flora and fauna under CMIP6 climate scenarios
MS#: GIGA-D-23-00183 Article type: Data Note

The maximum entropy technique was used by the authors to study the potential geographic distribution of thousands of terre
surroundings is improved by this painstaking and analytical work, which also provides essential insights into ecological manag

Without a doubt, this is a highly time-consuming and laborious task, therefore I'll just mention the minor changes below, whi

Thank you for your thoughtful and positive feedback about our analysis and data. Your recognition of the value of this researc
ensure transparency and robustness which we have also included below.

These include;

1. No doubt, MaxEnt perform really good with least number of occurrence points as well, please convey the minimum and ma

Thank you for this comment, it's a good idea to include the range of occurrence points somewhere in the manuscript. So, wha
have more than 1000 occurrence points, and the species with the most occurrence points is the Willie wagtail (Rhipidura leuc

We have added the following text into the manuscript:

Line 92: "Across all species, the median number of occurrence points was 123 and the distribution of the number of occurren

Figure 2 Distribution of occurrence points (n) for species models.

2. Line 72-74; and 86-87: Did you clip the environmental data to Australian extent and utilized to extract 10,000 background
having least number of occurrence points growing in any unique microhabitat (and in a small geographic land area) influence

Thank you for raising this point about how the background files were constructed, we recognise that we could have made this
manipulation and clipping already on:

Line 123: "All climate scenarios, bioclimatic variables were clipped to the extent of Australia prior to modelling."

To build on this, we will include more detail on the background file as you have suggested (based on your references to Line
taxonomic groups. To create the target group background files, we combined all occurrence points for all species within a tax
Australia.

To address this comment within the manuscript, we have added additional details within the methods section:

Line 98: "MaxEnt uses background sample points as pseudoabsences and recommends the use of target groups in sample sel
space. Each target group background file contained between 60,000 to 250,000 points depending on the taxonomic group, in

To address your final point about the use of a large background file for species with few occurrence points and this not being

Line 473: "While we did use target background files to reduce spatial biases (Barber et al., 2022), there may still be limitation
very specific micro-climate needs, therefore species-specific level grouping may help to overcome this. Background files that

3. As you targeted the terrestrial biota mainly, why NDVI or related measures were not considered to be used as important d

We acknowledge that a range of variables like NDVI (Wen et al., 2015) and weather more broadly (Reside et al., 2010) can b
an explanatory variable (Butt et al., 2013; Gallagher et al., 2019; Graham et al., 2019). Additionally, one major aspect of ou
the Maxent analysis. While we did not do this as this was out of the scope of our specific study, many of the bioclimatic varial
address why we didn't not use why NDVI up-front in the paper we have added the following text within the Limitations:

Line 482: “In relation to the variables used, we were primarily guided by past efforts that model the suitability of areas acros
as the normalized difference vegetation index (NDVI) (Wen et al., 2015), land use (Lentini & Wintle, 2015), weather (Reside
study is compatible with the species of interest, or whether additional information is required. This will likely be the case if th

4. To minimize the impact of sampling bias, a bias file (using species occurrence data and environment to estimate and devel
spatial bias like that which exists in the presence data (Please see;
https://aus01.safelinks.protection.outlook.com/?url=https%3A%2F%2Fdoi.org%2F10.3390%2Ff13050715&data=05%7C01%
I6Ik1haWwiLCJXVCI6Mn0%3D%7C3000%7C%7C%7C&sdata=HNgyy9z%2Fo8lLNLX%2B4wP%2B2SLaGniK5zB6X7kA8KmSw

Thank you for raising this point about spatial biases, we recognise that we could have made this much clearer within our met

To address this comment within the manuscript, we have added additional details within the methods section:

Line 98: “MaxEnt uses background sample points as pseudoabsences and recommends the use of target groups in sample sel
space. Each target group background file contained between 60,000 to 250,000 points depending on the taxonomic group, in

5. Though, overall variable importance is communicated, different species have different environmental requirements. I am u

The variable importance values for each variable are presented within the Maxent Report files for each species. Therefore, if r
and update. So, in the table provided, rather than including the current listings, we have just included an up-dated scientific a

6. AUC is a widely used metric, but have several inherent flaws, especially for Gaussian point processes, such as MaxEnt. AUC
a. AUC: a misleading measure of the predictive distribution models;
b. Without quality presence-absence data, discrimination metrics such as TSS can be misleading measures of model performa
Please include all the possible limitations of the work as mentioned above in the discussion section.

Thank you for raising this point about the importance of model validation and raising the challenges of using AUC and TSS to
measures. To address the second point, and the comment more generally, about assessing model performance and the role a

Model validation, from Line 211: “Model validation

Once variables were selected, models were re-run, and model performance was assessed based on the area under the curve
accuracy of the model and represents the probability that a randomly selected occurrence point is ranked higher than a rando
presences across the prediction gradients (Boyce et al., 2002; Hirzel et al., 2006a). The Boyce index value is represented by

The median AUC across all models was 0.97. AUC values of 0.7 or below indicates poor performance (Figure 2), 99.6% (n=10
across all models in this study was 0.97 (Figure 2). A Boyce index closer to 1 indicates that suitability predictions are consiste
between 0.5 and 0 and 0.05% (n=5) species had a value below 0 (1 bird and 4 vascular plants).

We have also provided a scatter plot summary of AUC in relation to Boyce Index. Based on the 0.7 threshold for AUC and the
not the AUC threshold and 1 species did not meet either threshold (Brown Falcon, Falco berigora). Prior to using species data

Figure 2 From left to right the plots are the distribution of AUC values, the distribution of Boyce Index (BI) values and a scatt

threshold is presented using a solid vertical line. These thresholds are also represented by solid lines on the scatter plot."

Finally, in the Limitations section we have also raised this concern:

Limitations, Line 513: "Finally, there is much contention around the best way to assess model performance of Maxent models
Index and do not consider the thresholds for these indexes prior to creating the habitat suitability projections, therefore the u

Reviewer #2:

The manuscript entitled "Habitat suitability maps for Australian flora and fauna under CMIP6 climate scenarios" uses a distrib
and has proper structure. The drawn conclusions are coherent with the obtained results. This is an important paper that need
conservation actions.

Thank you very much for your positive feedback on our manuscript, "Habitat suitability maps for Australian flora and fauna u
appreciated. We agree that while similar methodologies are prevalent, the unique results and information presented in our pa

Abstract
- Please outline the objective of the study clearly.
We have edited this sentence to now read:

Abstract, Line 6: "The objective of this paper is to develop habitat suitability maps for Australian flora and fauna under differe

- Please add some information on how suitable habitats would change by comparing current areas with future areas.
We have now added some information on how suitable habitats may change, this section now reads:

Abstract, Line 13: "Conclusions: The spatial data supplied can help identify important and sensitive locations for species unde
planning or species management, particularly under different climate change scenarios in Australia."

Methods

Line 81-82: I suggest citing the GCMs.

Line 124: "We have added the references to each GCM, "Bioclimatic variables were downloaded for eight global climate mode
(Hajima et al., 2020), MIROC6 (Tatebe et al., 2018), MRI-ESM2-0 (Yukimoto et al., 2019)"

See the reference list at the end of this document for the full references.

Line 96: "Model fitting" Please outline the model setting of Maxent. Model parameterization is important, yet minimal informa

Thank you for to this point about needing to clarify the model fitting section. To provide you with a clear understanding of the

Initial cross validation step:
java -mx2048m -jar /maxent.jar -e background.csv -s occurance.csv -o /species_folder nothreshold nowarnings novisible allo

Model fitting:
java -mx2048m -jar / maxent.jar -e /background.csv -s / occurance.csv -o /species_folder nothreshold nowarnings novisible

To address this comment within the paper, we have edited the model fitting section to now read:

Line 154: "All habitat suitability models were fit in MaxEnt Version 3.4.1 using the command line. Maxent models were first ru
applied and were later converted to values between 0 and 100. An example of the full Maxent model specification can be foun
is a text file containing the regression coefficients or lambdas fit by MaxEnt during modelling."

Line 136: "AUC", AUC alone is sometimes not sufficient to evaluate the performance of the model; other metrics such as Kap

Thank you for raising this point about the importance of model validation and raising the challenges of using AUC and TSS to
measures:

Methods, Line 214: "Once variables were selected, models were re-run, and model performance was assessed based on the a
measures the predictive accuracy of the model and represents the probability that a randomly selected occurrence point is ra

To address the second point, and the comment more generally, about assessing model performance and the role and/or suita

Model validation, from Line 211: "Model validation

Once variables were selected, models were re-run, and model performance was assessed based on the area under the curve
accuracy of the model and represents the probability that a randomly selected occurrence point is ranked higher than a rando
presences across the prediction gradients (Boyce et al., 2002; Hirzel et al., 2006a). The Boyce index value is represented by

The median AUC across all models was 0.97. AUC values of 0.7 or below indicates poor performance (Figure 2), 99.6% (n=1[illegible]
across all models in this study was 0.97 (Figure 2). A Boyce index closer to 1 indicates that suitability predictions are consiste
between 0.5 and 0 and 0.05% (n=5) species had a value below 0 (1 bird and 4 vascular plants).

We have also provided a scatter plot summary of AUC in relation to Boyce Index. Based on the 0.7 threshold for AUC and the
not the AUC threshold and 1 species did not meet either threshold (Brown Falcon, Falco berigora). Prior to using species data

Figure 2 From left to right the plots are the distribution of AUC values, the distribution of Boyce Index (BI) values and a scatt
threshold is presented using a solid vertical line. These thresholds are also represented by solid lines on the scatter plot."

Finally, in the Limitations section we have also raised this concern:

Limitations, Line 513: "Finally, there is much contention around the best way to assess model performance of Maxent models
Index and do not consider the thresholds for these indexes prior to creating the habitat suitability projections, therefore the u

Line 238: "subtracted from one another to accentuate areas". Reporting some information on how the suitability areas would

Thank you for this point, we will clarify where we have presented information on how suitability areas would change over tim

Lines 354: "To calculate spatial changes in habitat suitability through time, historical suitability maps can be subtracted from

Discussion
A brief paragraph on the implications of the applied technique and the huge data could be useful. In addition, how the applie

Thank you for raising the need to better emphasise the use potential in the context of additional applied techniques to work w
sections to describe the initial reuse potential of the data. To extend these sections and provide additional recommendations
Discussion, from Line 392:
"Applications for landscape and species conservation
This spatial and tabular dataset is ideal for users that would like to understand how the habitat suitability of areas for species
changes over time (Figure 6) or over space and time (Figure 5) (Hama & Khwarahm, 2023). This data can then be utilized in
useful to inform conservation (e.g., where to establish new protected areas), restoration or monitoring plans in areas which a

At the species level, this data set can be used to support conservation actions for species of interest (e.g., threatened species
species listing (IUCN, 2022). Spatial information about species could also be useful to compare the long-term suitability of ar
species (Eyre et al., 2022). Or could inform boarder scale biodiversity conservation analyses (Engert et al., 2023).
Applications in sustainability and natural capital accounting
Biodiversity forms a foundation of broader sustainability ideals, therefore, to measure progress towards sustainability, conser
indicators like the Species Threat Abatement and Restoration metric (STAR) (Mair et al., 2021) or the biodiversity intactness
consideration of biodiversity against other environmental or social values. This may include initiatives such as land use planni
Additionally, as many businesses are transitioning towards 'nature positive' the use of biodiversity to monitor business impac
Economic Accounting (SEEA) framework (UNEP et al., 2015), or could be used within sustainability assessments such as 'foot
like the Taskforce on Nature-Related Financial Disclosures (TNFD) (TNFD, 2023)."

Reviewer #3:

This manuscript presents habitat suitability of Australian flora and fauna under future climate change. The authors produced
species management, particularly under different climate change scenarios in Australia contribute towards evidence-based de
GigaScience.

Thank you for this overwhelmingly supportive review. We appreciate your shared vision of the potential importance and role

Reference List

Allouche, O., Tsoar, A., & Kadmon, R. (2006). Assessing the accuracy of species distribution models: prevalence, kappa and t
Barber, R. A., Ball, S. G., Morris, R. K. A., & Gilbert, F. (2022). Target-group backgrounds prove effective at correcting sampl
Biggs, R., & Scholes, R. J. (2005). A biodiversity intactness index. Nature, 434(7029), 45–49. http://go.galegroup.com/ps/i.d
Boucher, O., Servonnat, J., Albright, A. L., Aumont, O., Balkanski, Y., Bastrikov, V., Bekki, S., Bonnet, R., Bony, S., Bopp, L.,
Systems, 12(7), 1–52. https://doi.org/10.1029/2019MS002010
Breiner, F. T., Guisan, A., Bergamini, A., & Nobis, M. P. (2015). Overcoming limitations of modelling rare species by using en
Butt, N., Pollock, L. J., & Mcalpine, C. A. (2013). Eucalypts face increasing climate stress. Ecology and Evolution, 3(15), 5011
Engert, J. E., Pressey, R. L., & Adams, V. M. (2023). Threatened fauna protections compromised by agricultural interests in A
Eyre, A. C., Briscoe, N. J., Harley, D. K. P., Lumsden, L. F., McComb, L. B., & Lentini, P. E. (2022). Using species distribution
Gallagher, R. V., Allen, S., & Wright, I. J. (2019). Safety margins and adaptive capacity of vegetation to climate change. Scie
Gao, L., & Bryan, B. A. (2017). Finding pathways to national-scale land-sector sustainability. Nature, 544(7649), 217–222. h
Graham, E. M., Reside, A. E., Atkinson, I., Baird, D., Hodgson, L., James, C. S., & VanDerWal, J. J. (2019). Climate change a
Hajima, T., Watanabe, M., Yamamoto, A., Tatebe, H., Noguchi, M. A., Abe, M., Ohgaito, R., Ito, A., Yamazaki, D., Okajima, H
2244. https://doi.org/10.5194/gmd-13-2197-2020
Halpern, B. S., Frazier, M., Verstaen, J., Rayner, P. E., Clawson, G., Blanchard, J. L., Cottrell, R. S., Froehlich, H. E., Gephart
https://doi.org/10.1038/s41893-022-00965-x
Hama, A. A., & Khwarahm, N. R. (2023). Predictive mapping of two endemic oak tree species under climate change scenarios
Harley, D. (2023). Seven urgent actions to prevent the extinction of the critically endangered Leadbeater's possum (Gymnob
Hawke, T., Bino, G., Kingsford, R. T., Grant, T., Griffiths, J., Weeks, A., Tingley, R., Mccoll-Gausden, E., Serena, M., Williams
Hirzel, A. H., Le Lay, G., Helfer, V., Randin, C., & Guisan, A. (2006). Evaluating the ability of habitat suitability models to pre
Hoang, N. T., Taherzadeh, O., Ohashi, H., Yonekura, Y., Nishijima, S., Yamabe, M., Matsui, T., Matsuda, H., Moran, D., & Kar
Irwin, A., & Geschke, A. (2023). A consumption-based analysis of extinction risk in Australia. Conservation Letters, 16(3). ht
IUCN. (2022). The IUCN Red List of Threatened Species (IUCN). http://www.iucnredlist.org
Jiménez, L., & Soberón, J. (2020). Leaving the area under the receiving operating characteristic curve behind: An evaluation
Krasting, J. P., John, J. G., Blanton, C., McHugh, C., Nikonov, S., Radhakrishnan, A., Rand, K., Zadeh, N. T., Balaji, V., Durac
Lamb, E. G., Bayne, E., Holloway, G., Schieck, J., Boutin, S., Herbers, J., & Haughland, D. L. (2009). Indices for monitoring b
Lentini, P. E., & Wintle, B. A. (2015). Spatial conservation priorities are highly sensitive to choice of biodiversity surrogates ar
Ma, B., Zeng, W., Hu, G., Cao, R., Cui, D., & Zhang, T. (2022). Normalized difference vegetation index prediction based on th
Mair, L., Bennun, L. A., Brooks, T. M., Butchart, S. H. M., Bolam, F. C., Burgess, N. D., Ekstrom, J. M. M., Milner-Gulland, E.
explicit contributions to science-based species targets. Nature Ecology and Evolution, 5(6), 836–844. https://doi.org/10.1038
Maxwell, S. L., Reside, A., Trezise, J., McAlpine, C. A., & Watson, J. E. (2019). Retention and restoration priorities for climate
Minin, E., Veach, V., Lehtomäki, J., Pouzols, F. M., & Moilanen, A. (2014). A quick introduction to Zonation. Version 1 (for Zv
Phillips, S. J., Dudík, M., Elith, J., Graham, C. H., Lehmann, A., Leathwick, J., & Ferrier, S. (2009). Sample selection bias and
Reside, A. E., Vanderwal, J. J., Kutt, A. S., & Perkins, G. C. (2010). Weather, Not Climate, Defines Distributions of Vagile Bird
Schwager, P., & Berg, C. (2021). Remote sensing variables improve species distribution models for alpine plant species. Basic
Séférian, R., Nabat, P., Michou, M., Saint-Martin, D., Voldoire, A., Colin, J., Decharme, B., Delire, C., Berthet, S., Chevallier,

Present-Day and Future Climate. Journal of Advances in Modeling Earth Systems, 11(12), 4182–4227. https://doi.org/10.102[illegible]
Stock, F., Bryan, B., Nolan, M., Graham, P., Connor, J., Dunstall, S., Ernst, A., Stock, F., Nolan, M., Bryan, B., Graham, P., C[illegible]
Swart, N. C., Cole, J. N. S., Kharin, V. V., Lazare, M., Scinocca, J. F., Gillett, N. P., Anstey, J., Arora, V., Christian, J. R., Hanı[illegible]
4823–4873. https://doi.org/10.5194/gmd-12-4823-2019
Tatebe, H., Ogura, T., Nitta, T., Komuro, Y., Ogochi, K., Takemura, T., Sudo, K., Sekiguchi, M., Abe, M., Saito, F., Chikira, M.[illegible]
MIROC6. Geoscientific Model Development Discussions, 1–92. https://doi.org/10.5194/gmd-2018-155
TNFD. (2023). Recommendations of the Taskforce on Nature-related Financial Disclosures.
UNEP, UNSD, CBD, & NORAD. (2015). SEEA Experimental Ecosystem Accounting: Technical Recommendations (Issue Decem[illegible]
Valavi, R., Guillera-Arroita, G., Lahoz-Monfort, J. J., & Elith, J. (2022). Predictive performance of presence-only species distrib[illegible]
Voldoire, A., Saint-Martin, D., Sénési, S., Decharme, B., Alias, A., Chevallier, M., Colin, J., Guérémy, J. F., Michou, M., Moine[illegible]
Earth Systems, 11(7), 2177–2213. https://doi.org/10.1029/2019MS001683
Watermeyer, K. E., Guillera-Arroita, G., Bal, P., Burgass, M. J., Bland, L. M., Collen, B., Hallam, C., Kelly, L. T., McCarthy, M.[illegible]
Watts, M. E., Ball, I. R., Stewart, R. S., Klein, C. J., Wilson, K., Steinback, C., Lourival, R., Kircher, L., & Possingham, H. P. (2[illegible]
Wen, L., Saintilan, N., Yang, X., Hunter, S., & Mawer, D. (2015). MODIS NDVI based metrics improve habitat suitability mode[illegible]
Wu, T., Yu, R., Lu, Y., Jie, W., Fang, Y., Zhang, J., Zhang, L., Xin, X., Li, L., Wang, Z., Liu, Y., Zhang, F., Wu, F., Chu, M., Li,[illegible]
2977-2021
Yukimoto, S., Kawai, H., Koshiro, T., Oshima, N., Yoshida, K., Urakawa, S., Tsujino, H., Deushi, M., Tanaka, T., Hosaka, M., [illegible]
of the Meteorological Society of Japan, 97(5), 931–965. https://doi.org/10.2151/jmsj.2019-051
