# Supplementary material for: Habitat suitability maps for Australian flora and fauna under CMIP6 climate scenarios
Source: Gigascience. 2024 Mar 5;13:giae002. doi: 10.1093/gigascience/giae002 (PMC10939329; doi:10.1093/gigascience/giae002)
Supplement: giae002_Reviewer_1_Report_Original_Submission — Arshad Mahmood Khan, Ph.D. -- 8/13/2023 [file giae002_reviewer_1_report_original_submission.pdf]

Reviewer Report

Title: Habitat suitability maps for Australian flora and fauna under CMIP6 climate scenarios

Version: Original Submission **Date:** 8/13/2023

Reviewer name: Arshad Mahmood Khan, Ph.D.

Reviewer Comments to Author:

Title: Habitat suitability maps for Australian flora and fauna under CMIP6 climate scenariosMS#: GIGA-D-23-00183 Article type: Data NoteThe maximum entropy technique was used by the authors to study the potential geographic distribution of thousands of terrestrial vertebrate and plant species and estimated the potential effects of climate change. This data note contains a wealth of informative material. Our understanding of the complex interactions between Australian species and their surroundings is improved by this painstaking and analytical work, which also provides essential insights into ecological management and conservation. I also value the thoroughness of the analysis they conducted.Without a doubt, this is a highly time-consuming and laborious task, therefore I'll just mention the minor changes below, which are specifically connected to the work limitations and which should be mentioned in the discussion section.These includes;1. No doubt, MaxEnt perform really good with least number of occurrence points as well, please convey the minimum and maximum number of utilized occurrence points of the considered species in the text.2. Line 72-74; and 86-87: Did you clip the environmental data to Australian extent and utilized to extract 10,000 background points for all species? Or clipped the environmental data with different extent for each species based on minimum and maximum lat/long values of each species? Clarity is missing. Use of a large background especially for those species having least number of occurrence points growing in any unique microhabitat (and in a small geographic land area) influence AUC significantly.3. As you targeted the terrestrial biota mainly, why NDVI or related measures were not considered to be used as important discriminatory variables? Such measures are very important for species residing in dense forests.4. To minimize the impact of sampling bias, a bias file (using species occurrence data and environment to estimate and develop a two-dimensional kernel density raster) for each species can be generated and used in MaxEnt distribution modelling. The inclusion of such bias files in the MaxEnt modelling effectively manipulate the background, and introduce the same spatial bias like that which exists in the presence data (Please see; https://doi.org/10.3390/f13050715).5. Though, overall variable importance is communicated, different species have different environmental requirements. I am unable to access the relevant additional/supplementary material at the moment, and want to suggest that please convey the variable importance for highly endangered species (if yet not included in the supplementary data).6. AUC is a widely used metric, but have several inherent flaws, especially for Gaussian point processes, such as MaxEnt. AUC and TSS are frequently criticized by the researchers (1. AUC: a misleading measure of the predictive distribution models; 2. Without quality presence-absence data, discrimination metrics such as TSS can be misleading measures of model performance, hence, partial AUC-ROC might be relatively more appropriate measure). Please include all the possible limitations of the work as mentioned above in the discussion section.

Level of Interest

Please indicate how interesting you found the manuscript: Choose an item.

Quality of Written English

Please indicate the quality of language in the manuscript: Choose an item.

Declaration of Competing Interests

Please complete a declaration of competing interests, considering the following questions:

- Have you in the past five years received reimbursements, fees, funding, or salary from an organisation that may in any way gain or lose financially from the publication of this manuscript, either now or in the future?
- Do you hold any stocks or shares in an organisation that may in any way gain or lose financially from the publication of this manuscript, either now or in the future?
- Do you hold or are you currently applying for any patents relating to the content of the manuscript?
- Have you received reimbursements, fees, funding, or salary from an organization that holds or has applied for patents relating to the content of the manuscript?
- Do you have any other financial competing interests?
- Do you have any non-financial competing interests in relation to this paper?

If you can answer no to all of the above, write 'I declare that I have no competing interests' below. If your reply is yes to any, please give details below.

I declare that I have no competing interests.

I agree to the open peer review policy of the journal. I understand that my name will be included on my report to the authors and, if the manuscript is accepted for publication, my named report including any attachments I upload will be posted on the website along with the authors' responses. I agree for my report to be made available under an Open Access Creative Commons CC-BY license (http://creativecommons.org/licenses/by/4.0/). I understand that any comments which I do not wish to be included in my named report can be included as confidential comments to the editors, which will not be published.

Choose an item.

To further support our reviewers, we have joined with Publons, where you can gain additional credit to further highlight your hard work (see: https://publons.com/journal/530/gigascience). On publication of

this paper, your review will be automatically added to Publons, you can then choose whether or not to claim your Publons credit. I understand this statement.

Yes Choose an item.
