# Supplementary material for: Habitat suitability maps for Australian flora and fauna under CMIP6 climate scenarios
Source: Gigascience. 2024 Mar 5;13:giae002. doi: 10.1093/gigascience/giae002 (PMC10939329; doi:10.1093/gigascience/giae002)
Supplement: giae002_Reviewer_3_Report_Original_Submission — Masoud Yousefi -- 9/7/2023 [file giae002_reviewer_3_report_original_submission.pdf]

Reviewer Report

Title: Habitat suitability maps for Australian flora and fauna under CMIP6 climate scenarios

Version: Original Submission **Date:** 9/7/2023

Reviewer name: Masoud Yousefi

Reviewer Comments to Author:

This manuscript presents habitat suitability of Australian flora and fauna under future climate change. The authors produced habitat suitability maps under RCP2.6-SSP1, RCP4.5-SSP2, RCP7.0-SSP3 and RCP8.5-SSP5 climate futures for 1,382 terrestrial vertebrates and 9,251 vascular Plants. The research can be used for landscape and conservation planning or species management, particularly under different climate change scenarios in Australia contribute towards evidence-based decision-making regarding demarcation of protected areas in Iran. Overall, the manuscript is well written with sufficient presentation of research aims, methodologies, key results and outcomes. I recommend this manuscript for publication in GigaScience.

Level of Interest

Please indicate how interesting you found the manuscript: Choose an item.

Quality of Written English

Please indicate the quality of language in the manuscript: Choose an item.

Declaration of Competing Interests

Please complete a declaration of competing interests, considering the following questions:

- Have you in the past five years received reimbursements, fees, funding, or salary from an organisation that may in any way gain or lose financially from the publication of this manuscript, either now or in the future?
- Do you hold any stocks or shares in an organisation that may in any way gain or lose financially from the publication of this manuscript, either now or in the future?
- Do you hold or are you currently applying for any patents relating to the content of the manuscript?
- Have you received reimbursements, fees, funding, or salary from an organization that holds or has applied for patents relating to the content of the manuscript?
- Do you have any other financial competing interests?
- Do you have any non-financial competing interests in relation to this paper?

If you can answer no to all of the above, write 'I declare that I have no competing interests' below. If your reply is yes to any, please give details below.

I declare that I have no competing interests.

I agree to the open peer review policy of the journal. I understand that my name will be included on my report to the authors and, if the manuscript is accepted for publication, my named report including any attachments I upload will be posted on the website along with the authors' responses. I agree for my report to be made available under an Open Access Creative Commons CC-BY license (http://creativecommons.org/licenses/by/4.0/). I understand that any comments which I do not wish to be included in my named report can be included as confidential comments to the editors, which will not be published.

Choose an item.

To further support our reviewers, we have joined with Publons, where you can gain additional credit to further highlight your hard work (see: https://publons.com/journal/530/gigascience). On publication of this paper, your review will be automatically added to Publons, you can then choose whether or not to claim your Publons credit. I understand this statement.

Yes Choose an item.
